# Tracing the evolutionary histories of ultra-rare variants using variational dating of large ancestral recombination graphs

**DOI:** 10.64898/2026.01.07.698223

**Published:** 2026-01-12

**Authors:** Nathaniel S. Pope, Sam Tallman, Ben Jeffery, Duncan Robertson, Yan Wong, Savita Karthikeyan, Peter L. Ralph, Jerome Kelleher

**Affiliations:** 1 Institute of Ecology and Evolution, University of Oregon, Eugene OR 97402, USA; 2 Genomics England; 3 Big Data Institute, Li Ka Shing Centre for Health Information and Discovery, University of Oxford, OX3 7LF, UK; 4 Department of Data Science, University of Oregon, Eugene OR 97402, USA

## Abstract

Ultra-rare variants dominate whole-genome sequencing datasets, yet their interpretation is limited by allele frequency, which provides little information at very low counts and is highly sensitive to uneven ancestry representation. Allele age offers an ancestry-agnostic alternative but existing methods do not scale to biobank-sized cohorts. Here we present a scalable variational algorithm for dating Ancestral Recombination Graphs (ARGs), implemented in tsdate, together with new distributed methods enabling practical biobank-scale ARG inference using tsinfer. Applied to 47,535 genomes from the Genomics England 100,000 Genomes Project, we infer contiguous ARGs spanning 206 Mb and estimate ages for 23.2 million variants, including 11.8 million singletons. ARG-based allele ages remain accurate under extreme sampling imbalance and, in real data, reveal signatures of purifying selection and clinically relevant heterogeneity among variants with identical observed frequencies. Estimates for recent mutations are precise only at large sample sizes, highlighting the information accessible in the haplotype structure of large datasets. Biobank-scale ARGs therefore enable robust, ancestry-agnostic age estimation for ultra-rare variation with broad utility for statistical and clinical genomics.

## Introduction

Allele frequency is one of the most widely used descriptors of genetic variation, informing applications that range from clinical variant interpretation^1^ and association testing^2^ to evolutionary inference and models of natural selection.^3–5^ However, the interpretation of allele frequency is fundamentally constrained by how datasets are assembled. In contemporary human datasets, frequency depends not only on evolutionary processes but also on sample size and the geographic, cultural, or clinical contexts in which individuals are recruited, and there are fundamental issues with stratification by ancestry grouping.^6^ Classical population-genetic guidance typically assumes discrete, well-defined populations, an assumption increasingly inconsistent with the continuum of human genetic diversity.^7–9^ Many individuals cannot be unambiguously assigned to a single ancestry group^10^, and large cohorts are usually sampled in highly unbalanced ways.^11^ As a result, allele frequency remains informative for some questions, but for the vast majority of variants (which are ultra rare) its dependence on dataset composition and arbitrary or unstable group definitions makes interpretation difficult and often misleading.^12^ Furthermore, tools designed for discrete population groups often exclude individuals whose ancestry falls into a mixture of standard population labels.

Allele age offers a complementary and, in key respects, more direct summary of evolutionary history. Defined through genealogical relationships rather than population labels, the age of a mutation does not depend on which individuals happen to be included in a particular dataset, and is in principle comparable across cohorts with very different sampling schemes. Classical theory links age, frequency and selection, showing that strongly deleterious alleles are much less likely to be old than neutral alleles.^13, 14^ Early empirical work estimated ages for individual disease-causing mutations using locus-specific data.^15^ More recent approaches have produced tens of millions of genome-wide age estimates,^16–18^ but cannot scale to the billions of overwhelmingly ultra-rare variants now present in modern whole-genome sequencing (WGS) datasets.^19–21^ To obtain informative age estimates across the full allele frequency spectrum—especially for the ultra-rare variants that dominate WGS and are enriched for functional and clinical effects—we need methods that aggregate information along the genome, explicitly model local genealogical relationships, and remain computationally feasible at biobank scale.

Ancestral recombination graphs (ARGs) provide a principled framework for estimating allele ages because they place each mutation in its full genealogical context. For a given set of sampled DNA sequences, an ARG describes the haplotypes and inheritance relationships of all genetic ancestors of the sample, and consequently defines the genealogical trees for those samples at each position along the genome.^22, 23^ Mutation ages correspond to node times within these trees, so ARGs link neighbouring sites through shared ancestry and integrate information over long genomic regions rather than treating variants independently. This property is especially important for rare variation, where haplotype sharing carries most of the available signal. Recent methodological advances have made genome-wide ARG inference increasingly practical, fueling a rapid expansion of applications in population and statistical genetics.^24–28^ Broadly, inference methods fall into two classes. Model-based approaches^29–31^ sample from an explicit population-genetic model and can be highly accurate on simulation benchmarks,^32–35^ but are limited to at most a few hundred genomes and typically require specification of demographic parameters.^36^ Heuristic approaches^37–40^ scale to tens or hundreds of thousands of samples without strong parametric assumptions and separate ARG topology inference from dating. A key feature of these large-scale heuristic methods is that they are ancestry-agnostic: latent population structure is expressed in the inferred ARG without requiring predefined population labels or assumptions about group boundaries.

However, dating ultra-rare alleles requires ultra-large, highly contiguous ARGs, at a scale that has not yet been produced. To obtain accurate ages for very recent mutations, ARG inference must simultaneously encompass large sample sizes and long, continuous genomic regions. Close relatives share extended haplotypes that are identical by descent, manifesting as recent ancestors in the ARG, and artificially truncating these haplotypes by inferring in short chunks (e.g., to distribute computation across a cluster) removes much of the information that constrains their ages and biases date estimates upwards. Similarly, existing general-purpose dating methods,^41, 42^ cannot scale to ultra-large ARGs with tens of millions of nodes and mutations. As a result, there is currently no practical way to obtain accurate, uncertainty-aware ages for the billions of mostly ultra-rare variants in contemporary WGS cohorts.

Here we address this gap by developing a scalable variational ARG-dating algorithm, implemented in tsdate, together with new distributed methods in tsinfer that enable practical biobank-scale ARG inference. The variational algorithm uses expectation propagation to approximate posterior distributions for node times in large ARGs, providing principled, uncertainty-aware age estimates with substantially improved accuracy and efficiency over previous implementations. The distributed tsinfer pipeline infers ARG topologies over long, contiguous genomic segments for tens of thousands of high-coverage genomes. Applied to 47,535 genomes from the Genomics England 100,000 Genomes Project,^19^ we use these methods to infer ARGs spanning 206 Mb and to estimate ages for 23.2 million variants, including 11.8 million singletons. We show that these allele ages remain accurate under extreme sampling imbalance, reveal genealogical structure that is invisible to allele frequency alone, capture signatures of purifying selection from individual variants to regional constraint, and stratify clinically classified variants even at identical observed frequencies. Together, these results demonstrate that scalable ARG inference and dating can now deliver robust, ancestry-agnostic ages for ultra-rare human variation at biobank scale, providing a temporal framework that links evolutionary history, functional impact, and clinical interpretation.

## Results

### Variational Gamma ARG dating

**Figure 1:**
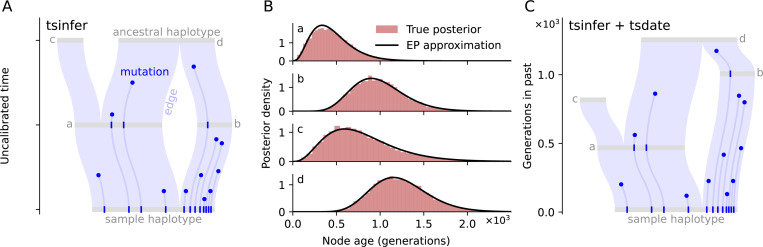
Tsinfer and tsdate use scalable approximations to infer variant ages from biobank-scale sequence data. From left to right: (A) ARGs consists of nodes (ancestral haplotypes) connected by edges (patterns of inheritance) that are localized in time (y-axis) and along the reference sequence (x-axis). Tsinfer infers an ARG topology that is consistent with observed genetic variation in contemporary samples. (B) The density of mutations along edges in the inferred ARG is used by tsdate to estimate (variational) posterior distributions for the ages of nodes. The variational posteriors closely approximate the true posteriors, calculated here by rejection sampling for the ARG shown in (A). (C) The posterior means are used to time-calibrate the ages of nodes in the ARG. Posteriors for mutation ages are derived from those of node ages by assuming that the age of a mutation is equally likely over the length of its edge, and integrating over the ages of the parent and child.

We developed an efficient and accurate method to estimate the age of each ancestral node in an inferred ARG based on a Variational Gamma (VG) approximation of their joint posterior distribution, that is implemented in tsdate version 0.2 (Fig. 1). This implementation uses a Bayesian message-passing algorithm called Expectation Propagation^43^ (EP) to rapidly estimate variational posteriors for both nodes and mutations (Methods §1). These approximate posteriors are compact and capture uncertainty in a principled manner. Posteriors are calibrated by rescaling the ARG to match the average mutation count per sample (Methods §2), and we regularise the ages of unconstrained roots via an exponential prior to avoid unrealistically deep node ages (Methods §3). The method also incorporates a principled approach to coestimating the phase and age of singletons (Methods §4), facilitating the analysis of this large and phenotypically important class of variant.^44^

**Figure 2:**
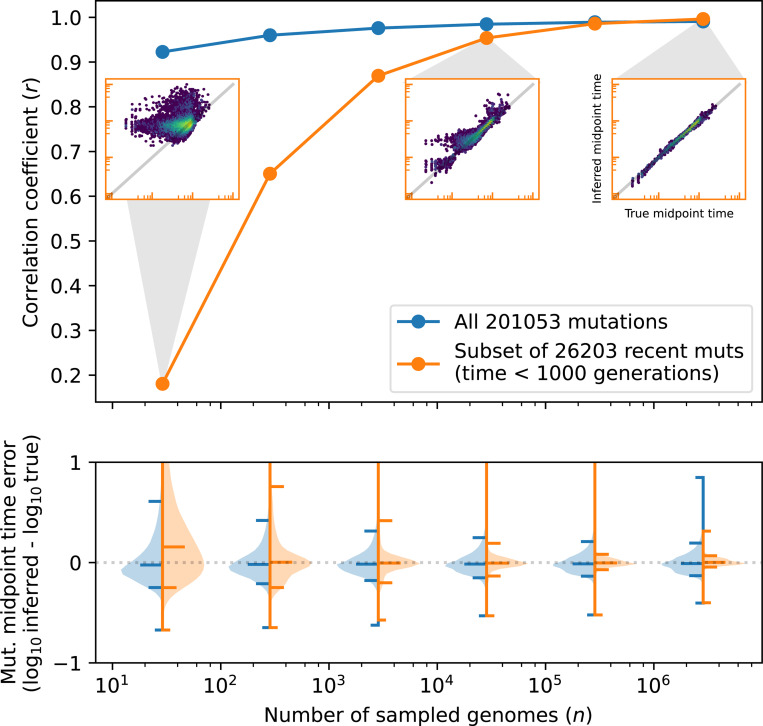
Accuracy of tsdate inference on a pedigree-based simulated tree sequence of 2.85 million samples of human chromosome 17.^45^ Successively smaller subsamples were extracted by simplification, resulting in a smallest tree sequence of 29 samples (0.001% of the original) and 201053 mutations, and tsdate was run on each. In both plots, results are shown for either those 201053 mutations found in all subsamples (blue), or on the subset of these with true time more recent than 1000 generations (orange). Upper: correlation between true and inferred mutation times as a function of sample size (distributions on which r is based shown as inset scatterplots); true times are taken as midpoints of node dates above and below the mutation. Lower: error distributions, with bars showing median, 5% and 95% quantiles for errors in log_10_ dates.

The new VG algorithm is a major improvement over the “inside-outside” (IO) method implemented in tsdate version 0.1^41^ in terms of both accuracy (Fig. S1, Tab. S1, Methods §5) and scalability (Fig. S2). Implemented in Python, leveraging the efficient tskit data model^46, 47^ and Numba acceleration,^48^ the VG algorithm can be applied to the largest available ARGs, requiring only 15 minutes and 7.7GB of RAM to date a simulated ARG of 1 million human chromosomes^45^ on a single thread of an AMD EPYC 7742 CPU. In addition to this scalability, Fig. 2 shows that the accuracy of the VG algorithm improves as sample size grows (in contrast to IO; Fig. S2). Accuracy is comparable to a recently-developed Markov chain Monte Carlo (MCMC) algorithm^42^ that requires orders of magnitude longer runtime for large sample sizes (Fig. S2, Methods §6). Benchmarking indicates that the expectation propagation scheme used by the VG algorithm converges within a small number of iterations (regardless of sample size, Fig. S3) to approximate posteriors that provide well-calibrated uncertainty estimates (Fig. S4).

To evaluate performance on real data, we ran the combination of tsinfer and the VG algorithm (tsinfer+tsdate, hereafter) on WGS data from the 1000 Genomes Project^49^ (1KGP, Methods §7) and validated against previous results providing estimates of the ages of young, intermediate and ancient variants. We found that inferred ages of > 99% of recent variants were consistent with their first appearance in the ancient DNA record^50^ (Fig. S5, Methods §8); strong agreement with a recent estimate^51^ of the time and magnitude of the Out-of-Africa bottleneck (Fig. S6, Methods §9); and that age estimates of an ancient inversion on chromosome 17q closely correspond to previous work^34^ (Fig. S7, Methods §10). We also evaluated the dependence of mutation age on sample size, showing that mutation ages inferred from subsets of the 1KGP cohort correlate well (Fig. S8). To validate singleton dating, we dated 106,863 singletons in a subset of 1,500 individuals using their estimated phase^49^ and found that the resulting ages correlate more strongly with those from the phase-agnostic method (r=0.807) than with ages obtained under random phasing (r=0.550; Fig. S9). Finally, we compared four published allele age estimates (CoalNN,^18^ GEVA,^17^ Relate,^37^ and SINGER^31^) for 1KGP with tsinfer+tsdate (Fig. S10, Methods §11). Overall, there is considerable variation between the methods, with correlation coefficients ranging from 0.683 (CoalNN/SINGER) to 0.835 (Relate/tsinfer+tsdate), consistent with previous comparisons.^52^

Crucially, tsinfer+tsdate are robust to the biased sampling common in contemporary datasets. To illustrate this we performed a large realistic forwards-time population genetic simulation incorporating negative selection (Methods §12). After running tsinfer and tsdate on the simulated data, the allele ages estimated are accurate and unbiased, even under the highly unbalanced sampling used to mimic the GEL dataset discussed in later sections (Fig. S11). The simulation also illustrates a more fundamental point. It has been shown that allele age offers little information about selection advantage above allele frequency with uniform sampling.^53^ However, the simulation shows that biased sampling induces complex and geographically structured distortions in allele frequency, while allele age remains straightforwardly interpretable. Additionally, in this simulation the combination of allele age and frequency has significantly more power to predict deleteriousness than frequency alone (SI §9).

### Inferring large contiguous ARGs for GEL

Although ARGs have been inferred for hundreds of thousands of samples using genotype array^38, 39^ and imputed^40^ data, there is a considerable gap to the variant densities required for chromosome-scale WGS data. The most recent method, Threads, was applied to approximately 10 million imputed variants genome wide for 487,409 samples, split into 136 chunks,^40^ each of size 30cM (an average of ∼74,000 variants per chunk). Current biobank-scale WGS datasets, however, consist of around 1 *billion* variants.^21, 54^ In addition to the challenge posed by the number of variants, dating recent, rare alleles requires ARG inference to be performed on long, contiguous regions. For example, close relatives will share long haplotypes that are identical by descent and manifest as recent ancestors (nodes) in the ARG. Artificially truncating the mutational area beneath these recent ancestors will bias their estimated ages upwards (Fig. S12). Thus, to infer accurate dates for recent mutations, we must have both large sample sizes *and* large sections of genome.

To enable such large-scale contiguous ARG inference we added the ability to perform inference distributed over a cluster in tsinfer version 0.4 (Methods §13). The approach distributes work in a deterministic manner, yielding the same results as a single-machine run. We applied this new pipeline to high coverage WGS data for 47,535 individuals in the Genomics England (GEL) 100,000 Genomes Project aggregated (aggV2) dataset^54^ (Methods §14). A central element of tsinfer’s algorithm infers ancestral haplotypes using heuristics. This approach does not perform well in variant-poor regions of the genome, often resulting in ancient haplotypes artefactually spanning such regions. To avoid this, we split chromosome arms into segments based on a site-density filter, yielding 15 segments, ranging from 1.36 to 33Mb, over chromosomes 16–22 (Tab. S2). Totalling 206Mb (around 14% of the ungapped genome), these regions have a somewhat higher recombination rate and gene density than the genome-wide average because of the bias towards short chromosomes, but are typical in terms of previous allele age estimates (Tab. S2, Fig. S13, SI §8). We performed ARG inference over a total of 11,445,983 non-singleton sites, which required about 16 CPU years (Tab. S3). We then dated the ARGs and phased and dated an additional 11,778,316 singletons, resulting a total of 16.8 million nodes, 141 million edges, 23 million mutations and 2.5GB file size (Tab. S4). We now focus on analysing the final dataset of 23,224,276 allele ages, and in particular on chromosome 17 for several analyses as the largest and most complete chromosome (Methods §14).

### Allele ages in GEL

The ages of the 23 million alleles studied span from very recent to deeply ancient, with 60.8% (*n* = 14, 043, 512) estimated to have occurred within the past 100 generations and 2.3% (*n* = 540, 318) exceeding 10,000 generations (Fig. S14). The frequency spectrum is dominated by ultra-rare variants (Fig. S15), with 94.8% (*n* = 21, 886, 991) having derived allele frequency (DAF) < 0.1% and 41.4% (*n* = 9, 617, 785) absent from gnomAD v4.1.^55^ Allele age correlates positively with DAF (Spearman’s ρ=0.594, *p* < 10^−300^), increasing from a geometric mean of ∼51 generations for singletons to ∼20,931 generations for common variants (DAF > 10%) (Figs. S16, S17).

As in previous studies,^17, 56^ we find that allele age also reflects functional consequence (Methods §15; Figs. S18, S19). Missense mutations (*n* = 336,127, geometric mean age 81 generations) are younger than non-coding (*n* = 22,517,141, 100 generations) and synonymous variants (*n* = 180,982, 104 generations), whilst the youngest consequence classes are protein-truncating (*n* = 9,994, 63 generations) and splice-altering mutations (*n* = 5,332, 58 generations), consistent with their typically higher functional impact and stronger selective constraint.

### Allele age and ancestral diversity

**Figure 3:**
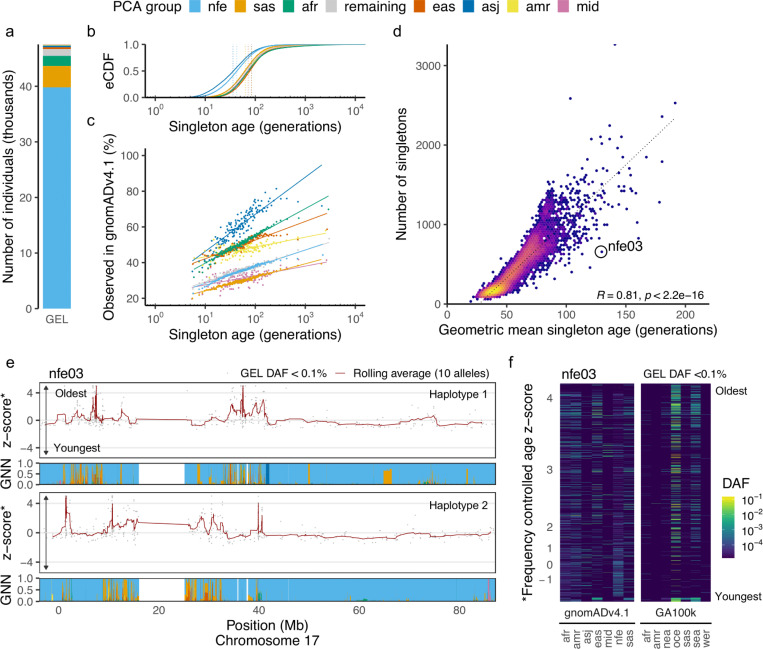
Mutation ages capture ancestral diversity in the 100,000 Genomes Project. (a) Representation of continental “PCA groups” assigned using a classifier trained on gnomADv4.1; see text for abbreviations. (b) Distribution of tsdate inferred ages of singleton alleles (GEL DAC = 1) per PCA group. Dotted lines show the geometric mean of each distribution. (c) Per PCA group, the proportion of singleton mutations at a given mutation age that were observed in gnomADv4.1 (100 quantiles per PCA group). (d) Relationship between number and geometric mean age of singleton variants observed per individual. A participant, nfe03 (ID anonymised for privacy), is highlighted as an example of a member of the NFE PCA group with an excess of older singletons compared to the majority of other members. (e) Frequency-controlled mutation age z-scores (normalised per discrete DAC bin) for ultra-rare (GEL AF < 0.1%) alleles along the two chromosome 17 haplotypes of nfe03. Local GNN proportions colored by PCA group are also shown. We note that the apparent mosaic of divergent lineages inherited from both parents is indicative of switch errors that occurred during phasing. (f) Heatmap depicting external group-specific frequencies of ultra-rare variants observed in nfe03 across gnomADv4.1 (genomes) and GenomeAsia 100k. The y-axis is arranged by age z-score from lowest (bottom) highest (top).

The GEL aggV2 dataset is a clinically recruited cohort of rare-disease and cancer patients^57^ derived from the 100,000 Genomes Project.^19^ The dataset broadly reflects the UK patient population, with participants predominantly of European descent.^58, 59^ For downstream descriptive analyses, we grouped participants using a PCA-based classifier trained on gnomAD v4.1 (Methods §16) which indicates a strong imbalance of geographic ancestries: 83.7% of individuals (*n* = 39, 806) were assigned to the “non-Finnish European” (NFE) label with high confidence (classification probability > 0.8; Fig. 3a). These groupings are used only for interpretation, and do not enter the ARG inference or dating procedures. We refer to these as “PCA groups” throughout.

The unequal representation of geographical ancestries in the dataset is reflected in the genealogical relationships in the inferred ARGs and the distribution of mutation ages within discrete derived allele count (DAC) bins. Individuals assigned to the NFE PCA group exhibit a greater density of recent coalescence events (Methods §17, Fig. S20). In contrast, participants assigned to less well-represented PCA groups—including South Asian (SAS, *n* = 3, 837), African (AFR, *n* = 1, 827), and East Asian (EAS, *n* = 335)—show fewer recent coalescence events, consistent with older average times to shared ancestry within the sample. As expected, given their closer genealogical proximity on average to others in the dataset, the NFE PCA group carry singletons (Methods §18) that are on average 28 generations younger than those carried across the remaining individuals (geometric mean age: 43 generations vs. 71 generations for NFE and non-NFE, respectively; Fig.3b). Similar patterns occur in higher DAC bins (Fig. S21).

Inferred mutation age reveals additional biological signal within frequency bins. For example, among singleton variants, we observe a strong positive relationship between the estimated age of a mutation and its probability of being observed in gnomADv4.1 (Fig 3c). This aligns with expectations: older mutations, having had more time to accumulate across more distantly related individuals, are more likely to be shared with external cohorts.

We also analysed how per-individual aggregated singleton ages varied across the cohort. We find that the number of singletons per individual is strongly correlated with the geometric mean age of those singletons (Spearman’s ρ=0.81, *R* = 0.81, *p* < 10^−16^; Fig. 3d), consistent with the expectation that deeper genealogies accumulate more mutations on average. Notably, this relationship is not only apparent across the full dataset but also persists within PCA groups (Fig. S22), highlighting the continuous nature of ancestral relationships apparent within these groups.

We next asked whether these signals could reveal finer-scale ancestry structure across genomic regions within individual genomes. To extend our analysis beyond discrete allele counts and capture variation among all ultra-rare (DAF < 0.1%) mutations, we normalized log10-transformed mutation ages by z-scoring within discrete DAC bins (Methods §19), thereby controlling for the inherent relationship between allele frequency and age. This normalization enabled the identification of genomic regions where mutation ages deviated substantially from expectations conditioned on frequency. We find that these deviations correlate with tracts of differentiated ancestries in an individual, as inferred using the genealogical nearest neighbours (GNN)^38^ proportions. A notable example (Fig. 3e) highlights an individual assigned to the NFE PCA group with local ancestry tracts enriched for mutations with large positive age z-scores. Many of these variants are common polymorphisms (derived allele frequency > 1%) in the Oceanian subset of GenomeAsia 100K^60^ (Fig. 3f). Individuals of Oceanian descent have extremely limited representation in the 100,000 Genomes Project cohort. Consequently, older variants that are common in Oceania are likely to appear superficially rare due to sampling gaps.^61^ Indeed, across the full dataset, the 48,010 DAF < 0.1% variants common in Oceania are 12.9-fold enriched among the most ancient age-frequency outliers (z>4; logistic regression *p* < 10^−300^). This illustrative example shows that genealogically informed metrics such as mutation age can expose informative patterns of rare variant history that may otherwise be obscured when assessing allele frequencies alone. Importantly, however, these signals are dataset-relative: the same genome analysed in a different cohort could exhibit substantially different patterns, reflecting the interaction between allele frequency and genetic ancestries.

### Allele age and patterns of negative selection

**Figure 4:**
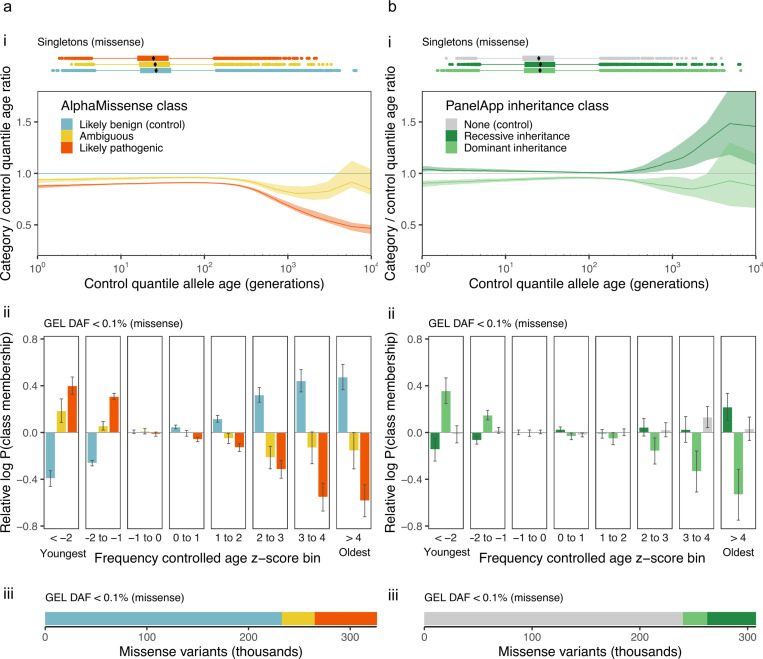
Allele age patterns indicate negative selection within coding regions. **(A) AlphaMissense.** (i) Quantile–quantile analysis of singleton missense variants (DAC=1): lines show the ratio of age quantiles for each AlphaMissense category relative to the corresponding quantiles for *likely benign*, with bootstrapped 95% confidence bands. For instance, the median age of *likely pathogenic* missense singletons is about 90% of the median age of *likely benign* missense singletons (which is around 15 generations ago). (ii) For ultra-rare missense variants (GEL DAF < 0.1%), the relative probability (odds ratios) of a variant being classified as *likely pathogenic* or *ambiguous* across frequency-controlled allele-age z-score bins (youngest < −2 to oldest > 4), estimated via logistic regression with 95% confidence intervals. (iii) Counts of ultra-rare missense variants by AlphaMissense class. **(B) PanelApp inheritance.** (i) Quantile–quantile analysis of singleton missense variants contrasting genes annotated as *dominant* or *recessive* against genes with *no disease annotation* (control), with bootstrapped 95% confidence bands. (ii) For ultra-rare missense variants (GEL DAF < 0.1%), the relative probability (odds ratios) of a variant falling in *dominant* or *recessive* disease genes across allele-age z-score bins (youngest < −2 to oldest > 4), from logistic regression with 95% confidence intervals. (iii) Counts of ultra-rare missense variants stratified by PanelApp inheritance class.

Deleterious variants are expected to have younger mutation ages compared to benign variants at the same frequency.^62–64^ To explore whether mutation age could provide information regarding deleterious variant effects, we began by evaluating the 326,121 ultra-rare (DAF < 0.1%) missense variants in our study dataset (Methods §15). Of these, 18.8% (*n* = 61, 402) are classified as likely pathogenic, 71.2% (*n* = 232, 346) as likely benign, and 10% (*n* = 32, 373) as ambiguous using AlphaMissense^65^ with default classification thresholds. In line with expectations of negative selection against functionally disruptive variants, likely pathogenic variants are significantly younger on average (geometric mean: 60 generations) than those classified as likely benign (80 generations; permutation test *p* < 10^−4^). The strongest effect of negative selection is expected to be a deficit of old deleterious alleles. Indeed, the quantiles of the age distribution of likely pathogenic alleles are lower than those of likely benign alleles (Methods §20, Fig. 4a.i), particularly at the ancient tail of the distribution. Frequency-controlled mutation age z-score distributions (Fig. 4a.ii, Methods §21) show that this shift is observed not just in singletons, but across the ultra-rare allele frequency spectrum. Here, we find that likely pathogenic missense variants are depleted for mutations that are older than expected given their frequency (positive z-score bins) and enriched for those that are younger than expected (negative z-score bins). Consistent results are observed when using PrimateAI-3D^66^ to classify variant deleteriousness (Fig. S23).

Rare recessive deleterious alleles are largely masked from the effects of selection, while dominant alleles are more strongly affected. So, we next investigated whether stratification by clinically annotated modes of inheritance shows similar patterns (Methods §22). Specifically, we compared the distributions of ultra-rare (DAF < 0.1%) missense mutation ages within exons of Mendelian disease-associated genes, stratified into dominant (*n* = 22, 542 variants, 6.9%), recessive (*n* = 45, 502 variants, 14.8%), and non-Mendelian (unannotated; *n* = 239, 638 variants, 73.5%) classes based on expert assessed PanelApp^67^ annotations (Fig. 4b; Methods §15). As predicted, dominant disease genes are depleted for older-than-expected mutations and enriched for younger-than-expected mutations relative to overall missense background (Fig. 4b.i), while recessive disease genes show the opposite pattern.

**Figure 5:**
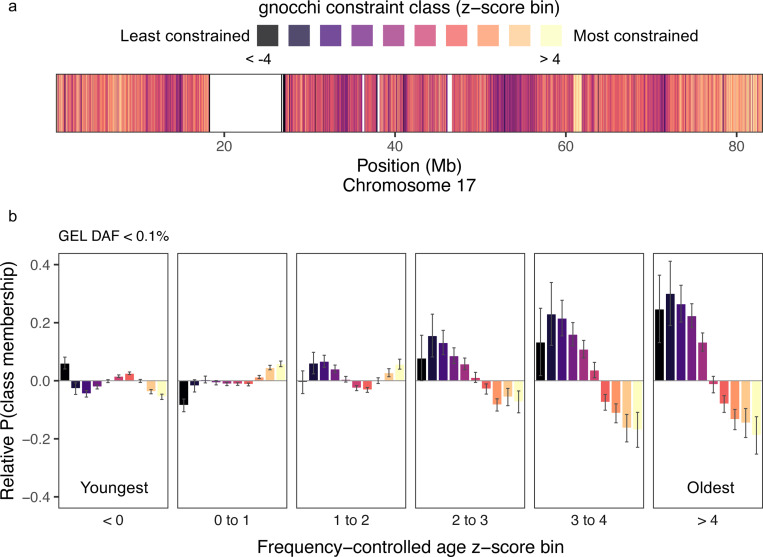
Allele age distributions reflects chromosome-wide patterns of selective constraint. (A) 10kb regions across chromosome 17 coloured by gnocchi constraint z-score bin (< −4 to > 4). (B) For all ultra-rare (DAF < 0.1%) variants across chromosome 17, the relative probability of a variant appearing within a given constraint z-score bin (10kb region) per frequency-controlled mutation age z-score bin (< 0 to > 4). Relative probabilities (odds ratios) were calculated using logistic regression, with error bars showing 95% confidence intervals.

Extending our analysis beyond missense variants, we examined all coding and non-coding ultrarare variants (DAF < 0.1%; *n* = 7, 552, 186) to assess how mutation age distributions relate to regional selective constraint. Using gnocchi^55^ constraint metrics derived from gnomADv3.1 mapped to 10 kb windows reveals a clear pattern (Fig. 5a). Mutations older than expected for their frequency are significantly enriched in the least constrained regions (gnocchi z < −4) and depleted in the most constrained regions (gnocchi z>4) (Fig. 5b).

Collectively, these analyses demonstrate that mutation age distributions capture biologically meaningful signatures of purifying selection across scales—from individual coding variants to broader patterns of regional constraint.

### Age of clinically classified variants

We analysed clinically classified missense or loss-of-function (LoF) variants in the GEL dataset (Methods §22). Among singletons, pathogenic and likely pathogenic (P/LP) variants were significantly younger than both benign/likely benign (B/LB) variants and variants of uncertain significance (VUS) (permutation test all *p* < 0.0001; Tab. S5), consistent with strong purifying selection acting on pathogenic alleles. Notably, P/LP singletons reported exclusively within the 100,000 Genomes Project are younger than those also reported in ClinVar (*p* = 0.0052), supporting the idea that older pathogenic variants are more likely to be shared across independent clinical datasets. We observed the same pattern among clinically classified doubletons: after removing likely recurrent mutations using a genealogical consistency filter (Methods §23, Fig. S24), P/LP variants again exhibit significantly younger ages than both B/LB variants (*p* = 0.0012) and VUS (*p* = 0.0066; Tab. S5).

**Figure 6:**
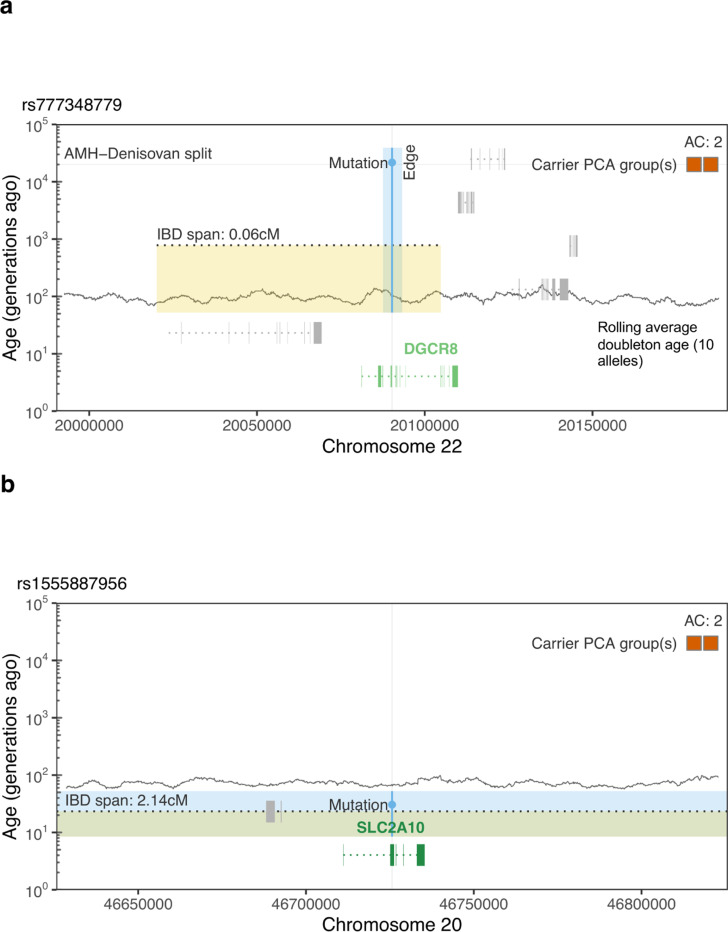
Distinct genealogical contexts underlying two variants of unknown clinical significance (VUS) at the same frequency. (a) Example of EAS doubleton (DAC = 2) VUS with deep inferred age. Allele age is contextualised using approximate dates of the AMH (anatomically modern human) Denisovan split (20,000 generations). (b) Example of EAS doubleton VUS with recent inferred age. Blue shaded areas represent edges upon which the mutations reside, with coordinates bounded by the edge span (x) and the node ages above and below the mutation (y). Orange shaded areas represent IBD haplotypes, defined here as the span where the node below the mutation represents the most recent common ancestor (MRCA) of both carriers, with y-coordinates bounded by the estimated node age and the recombination-based haplotype age (dotted black line). cM: centiMorgans, estimated using HapMapII recombination maps.

We next asked whether integrating allele age could identify variants with different evolutionary histories despite being matched by allele frequency, PCA group, and clinical classification (as above, PCA groups are used only for descriptive stratification; age estimation itself is label-free). For this, we focussed on non-recurrent doubletons from the four largest PCA groups in the GEL dataset (NFE, AFR, SAS, AFR; Fig. S25). Within this set, the oldest P/LP variant is estimated to be 339 generations old (95% CI: 49–900). In contrast, we identified six VUS in dominant genes with ages more than an order of magnitude older (4,827–35,340 generations; Tab. S6), which is likely inconsistent with the expectation of strong purifying selection on pathogenic Mendelian variants. One notable example was a missense mutation in *DCGR8*, a gene associated with early-onset nervous system tumours, estimated to be ∼21,100 generations old (95% CI: 4,900–59,300 generations; Fig. 6a). This variant is present in Denisovans but absent from Neanderthals and lies on a 46 kb (0.06 cM), identity-by-descent (IBD) haplotype shared among two EAS carriers, consistent with proposed dates of Denisovan introgression ∼50,000 years ago.^68^ Within the EAS PCA group, we further identified 22 VUS at the same allele count (DAC = 2) with ages ranging from 30 and 3,035 generations (geometric mean age = 74 generations). The youngest example was a missense mutation in *SLC2A10* (95% CI: 5–130 generations) located on a 1,129 kb (2.14 cM) shared IBD haplotype (Fig. 6b), indicative of recent shared ancestry among carriers.

Such findings illustrate that even among variants at the same apparent frequency, mutation age and genealogical structure can reveal substantial differences in evolutionary history, offering complementary evidence for variant interpretation.

## Discussion

Molecular dating of phylogenies enabled evolutionary processes to be interpreted on an absolute timescale, revealing patterns of divergence and speciation that were previously inaccessible. In human genomics, however, inference at comparably fine temporal resolution within the recent past has remained challenging, particularly for ultra-rare variants whose interpretation is poorly served by allele frequency alone. Here we show that scalable, uncertainty-aware estimation of allele ages from large ARGs can recover temporal structure within rare variation, providing a complementary perspective on recent mutational history with the potential to inform clinical practice. This was enabled by two key methodological developments. Firstly, we developed an accurate and efficient method to estimate the ages of nodes and mutations in an ARG, which captures uncertainty through a principled variational approximation of their posterior distributions. Secondly, we developed the methods to perform large contiguous ARG topology inference (necessary to accurately date ultra-rare alleles) distributed over a cluster. We then applied these advances to infer highly contiguous ARGs for nearly 50,000 individuals over 14% of the genome, estimating allele ages for 23 million variants. Our analyses show that these ages capture deep biological signals of demography and selection, inaccessible to allele frequency alone. Importantly, because our ARG topology and dating methods do not require any labelling or partitioning of individuals, these results are stable and interpretable under the biased sampling common to modern datasets.

Several factors affect the reliability of these age estimates. First, we assume that each observed allele arose from a single mutational event. While recurrent mutation is relatively rare in humans, it becomes increasingly relevant as analyses focus on ultra-rare variation at very large sample sizes,^69^ particularly in mutational hotspots and at those clinically important loci where multiallelic sites are common. Incorporating calibrated, context-dependent mutation rates^70, 71^ and extending ARG methods to better accommodate multiallelic sites are therefore important directions for future work. Second, our analyses focus primarily on rare, and typically recent, variants. Recent ancestry is often tightly constrained by long shared haplotypes, providing strong information for dating, whereas deeper genealogical structure is more weakly informed by the data. As a consequence, absolute ages in the deep past are sensitive to timescale calibration and to the regularisation required to stabilise root ages, and should be interpreted with appropriate caution. Importantly, relative age comparisons among rare variants are relatively unaffected.

We have shown that age contains information about the likelihood of deleteriousness, and so has potential to inform clinical prioritization of alleles in rare genetic disease diagnosis. The examples in Figs. 3 and 6 give a glimpse of how this might work. This is likely most helpful in narrowing the search: candidate variants who are inferred to be old might be deprioritized. This could greatly increase power in some cases: for instance, an individual who has inherited a small portion of the genome from an undersampled portion of the globe could have a large number of candidate private alleles; however, these would also be inferred to be old (and with high uncertainty), so attention could be directed to the smaller number of confidently recent variants.

While ARG inference has made major leaps in recent years and interest in applications is burgeoning,^35, 72–80^ there remains considerable work to do before it becomes a routine component of clinical and statistical genetics pipelines. Scalability is one axis: even with distributed implementations, WGS data at biobank scale remain a substantial challenge. Another is uncertainty: the credible intervals reported by tsdate are expected to be well-calibrated *given* the ARG, but do not incorporate uncertainty in ARG topology itself. At present, Bayesian methods that sample from a posterior on ARGs^29, 31^ are limited to much smaller sample sizes. Hybrid inference schemes that reconstruct the tightly constrained recent past using non-parametric, heuristic approaches and then apply model-based methods to the deep past are therefore attractive.^81^ As ARG methods mature, it will be important to develop systematic approaches to quality control and evaluation. Standardised simulations,^82–84^ richer comparison metrics that quantify differences between inferred and true genealogies,^23, 34^ and new visualisation tools^85–87^ are important steps. Here we evaluated our inferred ARGs by comparing tsdate ages and tree structures to multiple independent lines of evidence. A systematic collection of such benchmarks across multiple species would be a valuable resource for the field, ensuring that methods are robust to the vagaries of real data.

Inferred ages of common alleles have found diverse applications.^63, 64, 88–93^ Our work shows that accurate and uncertainty-aware allele ages can now be obtained for ultra-rare alleles. These ages provide a coherent temporal interpretation of ultra-rare variation and link genealogical structure with functional and clinical consequences across a heterogeneous cohort. As sequencing efforts expand to larger and more diverse populations^11, 94–96^ and genealogical methods continue to develop, allele age is likely to become an important statistic in human genomics, informing evolutionary, functional, and clinical analyses. By avoiding reliance on population labels, allele age provides a stable, transferable measure of variation that is well suited to large, ancestrally mixed datasets and to cross-cohort synthesis of rare variant information.

## Online methods

### 1 Variational dating algorithm

We develop an efficient and accurate method of estimating the ages of each ancestral node in an inferred ARG, using a Bayesian message-passing algorithm (Fig. 1). Each edge in an ARG represents the inheritance of a particular segment of genome by a *child* node from another, more ancestral *parent* node. The *area* of an edge is its genomic span in base pairs, multiplied by the time between parent and child nodes in generations. We assume that the number of mutations falling on this edge is Poisson distributed with a mean given by its area multiplied by the mutation rate. The conjugate prior to the Poisson is the Gamma distribution, and therefore if the edge lengths were unconstrained, a Gamma prior on each edge length would lead to a Gamma posterior. ARGs impose strong constraints on node ages,^41^ however, and the exact posterior of node times is therefore intractable. We use Expectation Propagation^43^ (EP) to compute an efficient and principled variational approximation of the posterior node times in an ARG.

More precisely, we place exponential priors on node times and obtain a Gamma distribution for each node that approximates its marginal posterior. Properties of exponential families^97^ allow us to decompose the marginal posterior of each node time into a product of per-edge log-linear “factors” that are refined iteratively by EP updates (Fig. S26). For a given edge, EP proceeds by removing the associated factors from the parent and child variational posteriors, forming a bivariate “cavity” distribution (Fig. S26B, blue contours). The cavity is multiplied by the Poisson likelihood for the edge, forming the target or “surrogate” distribution (Fig S26B, black contours). Finally, the variational posteriors for the parent and child are updated to match the mean and variance of the surrogate (Fig S26B, red contours). A key factor in the computational efficiency of this algorithm is that the moments of the surrogate distribution are expressed in terms of hypergeometric functions and efficiently updated using saddlepoint approximations.^98^ A single round of EP updates requires traversing all edges in the ARG from leaves-to-roots and then roots-to-leaves. Expectation propagation is not guaranteed to converge in general, but in practice we find rapid convergence to accurate variational posteriors for node ages (Figs. S3, S4).

Formally, let V and E be the sets of nodes and edges in the ARG, respectively, and let $t_{i}$ be the age of the ith node measured as generations in the past. We know the ages of a subset of sampled nodes S, and wish to infer ages for the remaining set of internal nodes N. For simplicity, assume that a given pair of nodes is connected by at most one edge and use ij∈E to denote the edge connecting parent i to child j (i.e., i is ancestral to j over a single contiguous genomic segment). This assumption is for notational convenience only, and the underlying model can accommodate an arbitrary number of edges between a given ancestor and descendant. Each edge has two dimensions: time $t_{i}-t_{j}$ measured in generations, and genomic span $s_{ij}$ measured in base pairs. If the per-generation, per-base probability of mutation is a constant μ≪1 and independent between bases, then the probability of $y_{ij}$ mutations occurring along the edge is Poisson,

(1) $$p_{ij}\left(y_{ij}|t_{i}-t_{j}\right)=\frac{{\left(\mu s_{ij}\right)}^{y_{ij}}}{\text{Γ}\left(y_{ij}+1\right)}{\left(t_{i}-t_{j}\right)}^{y_{ij}}\exp\left\{-\mu s_{ij}\left(t_{i}-t_{j}\right)\right\},$$

subject to the constraint $t_{j}<t_{i}$ for all ij∈E. Let p(t∣η) be a joint prior distribution on the ages of the internal nodes, with hyperparameters η. The posterior of node ages is then,

$$p(t|y,\eta )=Z{\left(y,\eta \right)}^{-1}p(t|\eta )\prod_{ij\in E}p_{ij}\left(y_{ij}|t_{i}-t_{j}\right)$$

where Z(y,η) is the appropriate normalizing constant.

Exact posterior inference of node ages is not tractable because Z is an integral over a high-dimensional polytope. Instead, we approximate the true posterior p by a product distribution $q_{\theta }$,

$$\begin{array}{l} q_{\theta }(t)=\prod_{i\in N}q_{\theta_{i}}\left(t_{i}\right)\approx p(t|y,\eta ),\\ q_{\theta_{i}}\left(t_{i}\right)\approx p\left(t_{i}|y,\eta \right)=\int p(t|y,\eta )dt^{\setminus i} \end{array}$$

where $q_{\theta_{i}}$ is an approximate Gamma marginal for the ith node with variational parameters $\theta_{i}$, and $t^{\setminus i}$ is the vector of node ages without the ith node. The advantage of this approximation scheme is that the integration problem becomes an optimization problem: find the values of θ that produce the best approximation according to some distributional measure of discrepancy, in our case the Kullback-Leibler (KL) divergence. Since Gamma distributions form an exponential family, minimizing KL divergence is equivalent to moment matching of sufficient statistics ϕ(t)={t,logt}:

(2) $$\underset{\theta }{\text{arg min}}\int p(t|y,\eta )\log\frac{p(t|y,\eta )}{q_{\theta }(t)}dt=\left\{\theta :E_{p}[\phi (t)]=E_{q_{\theta }}[\phi (t)]\right\},$$

where $E_{p}[\cdot ]$ denotes expectation with respect to p(t∣y,η), and ϕ(t) is the concatenation of Gamma sufficient statistics for each node.

The minimization in Equation (2) is still intractable, as $E_{p}[\phi (t)]$ also involves a high-dimensional integral. We use “expectation propagation”^43^ to approximate the global optimization problem with a series of tractable local problems. Because the approximate marginals $q_{\theta_{i}}\left(t_{i}\right)\propto \exp\left\{\phi \left(t_{i}\right)\cdot \theta_{i}\right\}$ are log-linear in the variational parameters $\theta_{i}$ (up to a normalizing constant), each may be factorized into

$$q_{\theta_{i}}\left(t_{i}\right)\propto \prod_{k}\exp\left\{\phi \left(t_{i}\right)\cdot \theta_{i,k}\right\}$$

subject to $\sum_{k}\theta_{i,k}=\theta_{i}$. Note that the individual factors have the same form as the original distribution, but are not required to have a finite integral (as long as their product can be normalized). Therefore, we reparameterize the approximate joint posterior as

$$q_{\theta }(t)=\prod_{i\in N}q_{i}\left(t_{i}\right)\propto \prod_{i\in N}q_{i,0}\left(t_{i}\right)\prod_{ij\in E}q_{i,ij}\left(t_{i}\right)q_{j,ij}\left(t_{j}\right),$$

with the shorthand $q_{i,ij}=\exp\left\{\phi \left(t_{i}\right)\cdot \theta_{i,ij}\right\}$, and requiring that $\theta_{i}=\theta_{i,0}+\sum_{j:ij\in E}\theta_{ij}+\sum_{j:ji\in E}\theta_{ji}$ defines a valid (normalizable) distribution for all i. This aligns the form of the posterior to match that of the likelihood (1), allowing us to work with terms grouped by edge, not by node. In words, the variational marginals for each node are split into “messages” that correspond to the prior $\left(q_{i,0}\right)$ and edges $\left(q_{i,ij}\right)$, and each has the same form as the unnormalized posterior.

Expectation propagation works by iteratively refining the messages, one at a time. For edge ij, the “cavity” distribution is the approximation without the ij-th factor,

$$q_{\theta }^{\setminus ij}(t)\propto \frac{q_{\theta }(t)}{q_{i,ij}\left(t_{i}\right)q_{j,ij}\left(t_{j}\right)}\propto \exp\left\{\phi \left(t_{i}\right)\cdot \left(\theta_{i}-\theta_{i,ij}\right)+\phi \left(t_{j}\right)\cdot \left(\theta_{j}-\theta_{j,ij}\right)\right\}\prod_{k\in V\setminus \{i,j\}}q_{k}\left(t_{k}\right).$$

The “surrogate” is the product of the cavity and the Poisson edge factor from the exact unnormalized posterior,

(3) $$q_{\theta }^{\setminus ij}p_{ij}(t)\propto q_{\theta }^{\setminus ij}(t)\times p_{ij}\left(y_{ij}|t_{i}-t_{j}\right).$$

Intuitively, the surrogate is an approximation of the posterior that is exact locally (for the edge being refined) and approximate elsewhere. The variational parameters can be updated by matching moments (minimizing KL divergence) against the surrogate: given a current set of parameters θ, we wish to find an updated set of parameters $\theta^{\prime }$ such that

$$E_{q_{\theta }^{\setminus ij}p_{ij}}[\phi (t)]=E_{q_{\theta^{\prime }}}[\phi (t)].$$

The crucial difference with the global problem (2) is that the surrogate is *mostly factorized*, so that $E_{q_{\theta }^{\setminus ij}p_{ij}}[\phi (t)]$ and $E_{q_{\theta^{\prime }}}[\phi (t)]$ only differ for nodes i and j (i.e., for $\phi \left(t_{i}\right)$ and $\phi \left(t_{j}\right)$). Thus, the only variational marginals that need modification are $q_{i}$ and $q_{j}$. The messages are updated by dividing the new approximate posterior by the cavity (equivalent to subtracting natural parameters), so the only changes between θ and $\theta^{\prime }$ are that $\theta_{i,ij}^{\prime }=\theta_{i}^{\prime }-\left(\theta_{i}-\theta_{i,ij}\right)$ and $\theta_{j,ij}^{\prime }=\theta_{j}^{\prime }-\left(\theta_{j}-\theta_{j,ij}\right)$.

What remains is to calculate moments of the surrogate (3). Rather than the sufficient statistics $E\left[t_{i}\right]$ and $E\left[\log t_{i}\right]$, it turns out to be more numerically stable to match $E\left[t_{i}\right]$ and $E\left[t_{i}^{2}\right]$, and in practice gives a nearly equivalent fit. To find the moments, first note that the density of the surrogate is proportional to

$$q_{\theta }^{\setminus ij}(t)p_{ij}\left(y_{ij}|t_{i}-t_{j}\right)\propto q_{\theta_{i}-\theta_{i,ij}}\left(t_{i}\right)q_{\theta_{j}-\theta_{j,ij}}\left(t_{j}\right)p_{ij}\left(y_{ij}|t_{i}-t_{j}\right)\prod_{k\notin \{i,j\}}q_{k}\left(t_{k}\right),$$

and so terms related to $t_{k}$ with k∉{i,j} do not appear. At this point, it is useful to switch to the canonical parametrization of the Gamma, so that $q_{i}\left(t_{i}\right)\propto \exp\left\{-t_{i}\beta_{i}+\left(\alpha_{i}-1\right)\log t_{i}\right\}$. The message is then parameterized as $q_{i,ij}\propto \exp\left\{-t_{i}\beta_{i,ij}+\gamma_{i,ij}\log t_{i}\right\}$, and the cavity parameters as ${\tilde{\alpha }}_{i}=\alpha_{i}-\gamma_{i,ij}$ and ${\tilde{\beta }}_{i}=\beta_{i}-\beta_{i,ij}$, so that

$$\begin{array}{l} q_{\theta_{i}-\theta_{i,ij}}\left(t_{i}\right)q_{\theta_{j}-\theta_{j,ij}}\left(t_{j}\right)p_{ij}\left(y_{ij}|t_{i}-t_{j}\right)\\ \;\propto t_{i}^{{\tilde{\alpha }}_{i}-1}t_{j}^{{\tilde{\alpha }}_{j}-1}{\left(t_{i}-t_{j}\right)}^{y_{ij}}e^{-{\tilde{\beta }}_{i}t_{i}-{\tilde{\beta }}_{j}t_{j}-\mu s_{ij}\left(t_{i}-t_{j}\right)}. \end{array}$$

Defining

(4) Fn=F12α˜j+n,α˜i+α˜j+yij+nα˜j+yij+n+1;μsij−β˜jμsij+β˜i,

where F12 is the Gauss hypergeometric function, we can write the first two moments as

(5) $$E\left[t_{j}\right]=\frac{{\tilde{\alpha }}_{j}\left({\tilde{\alpha }}_{i}+{\tilde{\alpha }}_{j}+y_{ij}\right)}{\left(\mu s_{ij}+{\tilde{\beta }}_{i}\right)\left({\tilde{\alpha }}_{j}+y_{ij}+1\right)}\frac{F_{1}}{F_{0}}$$

(6) $$E\left[t_{j}^{2}\right]=\frac{{\tilde{\alpha }}_{j}\left({\tilde{\alpha }}_{j}+1\right)\left({\tilde{\alpha }}_{i}+{\tilde{\alpha }}_{j}+y_{ij}\right)\left({\tilde{\alpha }}_{i}+{\tilde{\alpha }}_{j}+y_{ij}+1\right)}{{\left(\mu s_{ij}+{\tilde{\beta }}_{i}\right)}^{2}\left({\tilde{\alpha }}_{j}+y_{ij}+1\right)\left({\tilde{\alpha }}_{j}+y_{ij}+2\right)}\frac{F_{2}}{F_{0}}$$

(7) $$E\left[t_{i}\right]=E\left[t_{j}\right]\frac{\mu s_{ij}-{\tilde{\beta }}_{j}}{\mu s_{ij}+{\tilde{\beta }}_{i}}+\frac{{\tilde{\alpha }}_{i}+{\tilde{\alpha }}_{j}+y_{ij}}{\mu s_{ij}+{\tilde{\beta }}_{i}}$$

(8) $$E\left[t_{i}^{2}\right]=E\left[t_{j}^{2}\right]{\left(\frac{\mu s_{ij}-{\tilde{\beta }}_{j}}{\mu s_{ij}+{\tilde{\beta }}_{i}}\right)}^{2}+\frac{{\tilde{\alpha }}_{i}+{\tilde{\alpha }}_{j}+y_{ij}+1}{\mu s_{ij}+{\tilde{\beta }}_{i}}\left(2E\left[t_{i}\right]-\frac{{\tilde{\alpha }}_{i}+{\tilde{\alpha }}_{j}+y_{ij}}{\mu s_{ij}+{\tilde{\beta }}_{i}}\right).$$

Or, when node j is a contemporary sample $\left(t_{j}=0\right)$,

(9) $$E\left[t_{i}\right]=\left({\tilde{\alpha }}_{i}+y_{ij}\right){\left(\mu s_{ij}+{\tilde{\beta }}_{i}\right)}^{-1}$$

(10) $$E\left[t_{i}^{2}\right]=\left({\tilde{\alpha }}_{i}+y_{ij}\right)\left({\tilde{\alpha }}_{i}+y_{ij}+1\right){\left(\mu s_{ij}+{\tilde{\beta }}_{i}\right)}^{-2}.$$

The full derivation of Equations (4) through (10) is given in SI §1. In practice, we use a fast and numerically stable saddlepoint approximation^98^ to evaluate the hypergeometric series (SI §2). Equivalent moments are derived for noncontemporary samples in SI §3. The updated natural parameters are obtained from the moments by

$$\theta_{i}^{\prime }=\left[\frac{E{\left[t_{i}\right]}^{2}}{E\left[t_{i}^{2}\right]-E{\left[t_{i}\right]}^{2}}-1,-\frac{E\left[t_{i}\right]}{E\left[t_{i}^{2}\right]-E{\left[t_{i}\right]}^{2}}\right].$$

If necessary, $\theta_{i}^{\prime }$ is projected onto the feasible range (such that the marginals can be normalized).

Expectation propagation converges to a fixed point that is often very close to the global minimum of (2).^99^ We find that iterating over edges in increasing then decreasing time order (i.e., from leaves to roots and then roots to leaves) provides the most rapid convergence, although in theory the updates could be done in parallel. Finally, variational approximations to the posteriors of mutation ages are calculated from those of node ages after convergence (SI §4).

### 2 Timescale calibration via root-to-leaf distance

The expectation propagation update described in Methods §1 is local in scope; taking a noisy observation of the area of an edge and updating our posterior belief regarding the ages of the two attached nodes. However, the assumption that mutation follows a Poisson process implies a strong global property: that the number of mutations in a given time interval is equal in expectation to the sum of all edge areas intersecting that interval (multiplied by the per-base mutation rate). Therefore, after the per-node posteriors have been obtained by expectation propagation we rescale time globally to enforce an approximate molecular clock.

Unfortunately, we cannot expect this property to hold for ARGs produced by tsinfer. The reason is that tsinfer encodes uncertainty about the topology of genealogical trees via polytomies. If the true genealogies are actually binary across all positions, then these “spurious” polytomies will create an excess of edge area relative to mutational density, which will introduce bias into estimates of node ages.

Instead, we enforce a match between the average number of mutations carried by each sample in a given time interval and the length of the interval multiplied by the mutation rate. Intuitively, the number of neutral mutations carried by a sample measures the average distance between it and the roots, a quantity that is not impacted by spurious polytomies. Specifically, we find a piecewise-constant, monotonic transformation of time that results in an ARG satisfying this constraint. To do so, we use equally-spaced quantiles of mutation ages to determine a time discretization, use the posterior means of node ages to calculate edge areas, and integrate over the ages of mutations on each edge.

A second variational approximation is used to transform the posteriors themselves: we transform a lower and an upper quantile of each posterior, then find the gamma distribution that matches these transformed quantiles. This strategy only requires a binary search over time intervals and a few Newton iterations, and is far more efficient than matching mean and variance (that would be calculated by piecewise-constant integration). The algorithm requires a single pass over edges and a single pass over nodes, and is described in detail in SI §7.

This approach is inspired by the rescaling algorithm implemented in SINGER^31^ and POLEGON,^42^ that matches observed and expected numbers of mutations rather than the average count per sample. To assess the impact of spurious polytomies, we implemented both rescaling approaches in tsdate, and applied them to a 100 Mb simulated ARG containing 20,000 samples after collapsing branches without mutational support. When the ARG contains strictly binary trees, both approaches are similarly unbiased (Fig S27). However, when spurious polytomies are present, rescaling based on absolute mutation counts and edge area introduces a strong, time-dependent bias in node ages (Fig S27, “area rescaling”), that is largely eliminated by weighting mutations by the number of carriers (Fig S27, “path rescaling”).

### 3 Regularization of root ages

Information about the ages of nodes comes from noisy measurements of edge lengths—mutational density—that are propagated up the ARG from a fixed reference point (the sample ages). Typically, the oldest ancestral segments will each have very short spans, because of the cumulative action of recombination. Mutational density is sparse on these ancient haplotypes, and there is relatively little ancestral material that can constrain their ages. As a consequence, the posteriors of the oldest nodes typically have extremely high variance, and this is exacerbated in the variational approximations by the local nature of expectation propagation. Thus, we regularize the ages of “ultimate” roots (the nodes with no parents) via an exponential prior. This scheme acts as a soft constraint on the maximum height of the ARG.

Specifically, if the ith node has no parents, then it is given the prior $p\left(t_{i}|\eta \right)\propto \exp\left\{-\eta t_{i}\right\}$. As this is a special case of the Gamma distribution, the prior incorporates directly into the variational posterior as $\alpha_{i,0}=0$ and $\beta_{i,0}=\eta$. Rather than specify η *a priori*, we fit it via expectation maximization (EM) at the end of each round of EP updates. Let $\delta_{i}$ be a binary indicator of whether node i is without parents, and $q_{i,\setminus 0}$ be the variational posterior without the contribution of the prior (i.e., $\alpha_{i,\setminus 0}=\alpha_{i}$ and $\beta_{i,\setminus 0}=\beta_{i}-\left(1-\delta_{i}\right)\eta$). The surrogate model is $\prod_{i}q_{i,\setminus 0}\left(t_{i}\right)\exp\left(-\eta t_{i}\delta_{i}\right)$. The associated EM objective for η is $Q\left(\eta^{\prime }|\eta \right)=C-\delta_{i}E_{q_{i}}\left[t_{i}\right]\eta^{\prime }$ where C is a constant that does not depend on η, leading to the update

$$\eta^{\prime }=\frac{\sum_{i}\delta_{i}}{\sum_{i}\delta_{i}\alpha_{i}^{\setminus 0}/\left(\beta_{i}^{\setminus 0}+\eta \right)}.$$

For the applications in this paper involving very recent mutations, this simple regularization scheme works well while adding little additional overhead. However, it may be an oversimplification if the root ages themselves are of interest, especially if a large heterogeneous sequence is being dated. In this case, the regularization scheme may be generalized to a mixture of Gamma distributions, at the expense of computational cost (described in detail in SI §6).

### 4 Ambiguous singleton phasing

ARG inference methods require input genotypes to be phased, most often achieved by statistical methods.^100, 101^ While phasing tends to be quite accurate for common variants and ARG inference methods robust to switch errors in simulations,^102^ singletons require special consideration and are often omitted from analysis. Support for phasing singletons has only recently been introduced in SHAPEIT5,^101^ and is based on choosing the shorter of the two possible carrier haplotypes, as this is likely to be the older copy (and thus the more likely to have mutated at a given base). A similar rationale is used by Runtc,^16, 103^ where ancestor age is estimated directly from shared haplotype length.

Singleton mutations have no impact on the tree topologies produced by tsinfer. So, the analogous procedure with an inferred but undated ARG is to choose the shorter of the two edges that connect to the carrier individual as the correct phase. However, the choice of phase determines the allocation of mutations across terminal edges, and may therefore impact the estimated ages of ancestors and of the singleton mutations themselves. Thus, we adapted the model described in Methods §1 to integrate over both possible phases for each singleton mutation. By doing so, we propagate uncertainty about choice of phase into the variational posteriors for the ages of nodes and mutations, and therefore leverage information from the entire graph rather than just singleton edges. This modification requires splitting the edges above each leaf so that any given individual has at most two immediate ancestors (nodes) over the span of every attached edge. See the Supplement (SI §5) for a detailed description of the “phase-agnostic” EP algorithm.

In Fig. S28, we show the impact of phasing for singleton age estimates in a 100Mb inferred ARG of 20,000 diploids, simulated from an equilibrium population with msprime. Choosing singleton phase based on inferred edge span largely mitigates error due to incorrect phase, relative to a worst-case scenario where phase is randomly assigned. The phase-agnostic algorithm marginally improves upon this shortest-edge heuristic. See also Fig. S9 for a benchmark on 1KGP data.

### 5 Accuracy of mutation age estimates from inferred ARGs

To measure the accuracy of variational posteriors for mutation age under realistic inferences, we simulated ARGs with 15,000 diploids sampled uniformly across populations using stdpopsim^84^ (version 0.2.1) and msprime.^104^ The simulated ARGs are modelled after 40Mb of human chromosome 17, using the HapMapII recombination map^105^ and four different demographic models: AmericanAdmixture_4B18,^106^ OutOfAfricaArchaicAdmixture_5R19^107^ OutOfAfrica_3G09,^108^ and Zigzag_1S14.^109^ We inferred ARG topologies using tsinfer with default paramters, and used the tsdate VG (new) and IO (previous) algorithms to date mutations. We measured bias and RMSE in estimated mutation age for both methods, across frequencies of very rare mutations (< 0.001 in global frequency; Fig. S1, Tab. S1).

To measure the calibration of the posteriors produced by the VG algorithm, we calculated empirical versus observed coverage of true mutation ages for posterior intervals of various widths. Because we expect ARG topology inference by tsinfer to introduce errors that are not modelled by the VG algorithm, we dated both true and inferred ARGs with the expectation that posteriors from the latter would underestimate uncertainty (Fig. S4).

### 6 Dating algorithm benchmarking

To assess the performance of the variational gamma algorithm versus the previous version of tsdate (“inside-outside”),^41^ we used an ARG of human chromosome 22 from a large realistic simulation.^45^ We randomly subsampled the ARG (by simplification^110^) to sample sizes ranging from 10 to 1 million human chromosomes, for three replicates at each sample size. We also benchmarked POLEGON^42^ (version 0.1.3, commit 0c088b3). We used the default settings for POLEGON (100 MCMC samples with a thinning interval of 10, followed by 3 rescaling iterations).

For all three methods, we measured wall time using the time module of the python standard library, and peak memory usage using memray for python code. For POLEGON, the additional memory usage of the compiled binary used for MCMC sampling was profiled via GNU Time, and peak memory usage was taken as the maximum over the parent python process and subprocess. Wall time and peak memory were measured separately, to avoid additional overhead from memory profiling. Finally, we measure accuracy as the root mean squared error in log_10_ estimated node age.

### 7 1,000 Genomes Project ARG inference

We downloaded the phased 1000 Genomes dataset from https://ftp.1000genomes.ebi.ac.uk/vol1/ftp/data_collections/1000G_2504_high_coverage/working/20220422_3202_phased_SNV_INDEL_SV/. Notably, singletons are absent from this resource.^49^ We converted the VCF to VCF Zarr^111^ format using bio2zarr version 0.1.5, and used ancestral state information from Paten et al.^112^ to polarise alleles. We performed ARG inference using tsinfer following the same approach as the GEL dataset (Methods §14). Details of the regions inferred are given in Tab. S7. All 3202 samples were included.

We extracted singletons from the unphased high-coverage 1000G data (https://ftp.1000genomes.ebi.ac.uk/vol1/ftp/data_collections/1000G_2504_high_coverage/working/20201028_3202_raw_GT_with_annot/) using bcftools.^113^ We added SNPs to the inferred ARGs if they were biallelic, passed quality control, had no missing genotypes and had a derived allele present in a single sample. We then used tsdate 0.2.4 to phase singletons and date the ARGs using a mutation rate of 1.29 × 10^−8^. Summary statistics of the resulting ARGs are given in Tab. S7.

To evaluate sensitivity to sample size, we also inferred ARGs using tsinfer+tsdate on the long arm of chromosome 20 for randomly-sampled sets of 100, 300 and 1500 samples, ensuring that the proportion of every super-population in each sample was conserved. Results are shown in Fig. S8.

To test the effectiveness of tsdate’s phase-agnostic singleton dating, we identified mutations that are singletons in the 1500 sample subset but of higher frequency in the full 3202 sample dataset. Results are shown in Fig. S9.

### 8 Comparison to ancient DNA dates

We used ancient DNA data from the 1240K Allen Ancient DNA Resource (AADR)^50^ v62.0 for Fig S5. We converted genome data for chromosome 20 to VCF using convertf^114^ and PLINK,^115^ then lifted over from hg19 to hg38 using the bcftools^113^ liftover plugin together with the chain file from https://hgdownload.soe.ucsc.edu/goldenPath/hg19/liftOver/. We set ancient DNA sample ages using the AADR field “Date mean in BP in years before 1950 CE” and minimum site ages from the oldest aDNA sample containing the derived allele at that site. We compared these minimum site ages to the time of node ancestral to the mutation in the inferred 1KGP ARGs (Methods §7).

We define a site as consistent with the aDNA date if the node time converted to years is older than the minimum aDNA site age, using both a conservative assumption of an average human generation of time of 25 years, and a more lenient assumption of 27 years.^116^

### 9 Comparison to PHLASH

We compared the inferred times of human effective population size $\left(N_{e}\right)$ changes as derived from the 1KGP inferred ARGs (Methods §7) with independent estimates from a recent study.^51^ Fig S6 shows data from Fig. 3a of Terhorst^51^ (obtained from https://github.com/jthlab/phlash_paper/blob/master/data/fig7b.csv) along with the inverse of the instantaneous coalescence rate (equivalent to $N_{e}$) computed from chromosome 20 of the inferred ARGs using the tskit pair_coalescence_rates function. Coalescent times are comparable, as both methods use the same mutation rate (1.29 × 10^−8^). For ease of display we focus on three continental groupings (AFR, EUR and EAS) present both in the PHLASH analysis and in our 1KGP inferred ARGs.

### 10 Dating the 17q21.31 inversion

To estimate the age of the 17q21.31 inversion with tsinfer+tsdate, we extracted the identifiers and associated H1 and H2 alleles of 21 marker SNPs from Table 2 of Donnelly et al.,^117^ where H1 and H2 correspond to non-carriers and carriers of the inversion, respectively. We excluded the SNP labeled E_TAUIVS11_10 because we could not confirm its hg38 genomic coordinates. For the remaining 20 SNPs, we used their RSids to retrieve hg38-mapped positions from https://myvariant.info/. Of these, 18 were present in the inferred 1KGP ARG (Methods §7); three additional markers were excluded because the derived or ancestral allele in the ARG did not match either the H1 or H2 allele, rendering carrier status ambiguous. We assigned a haploid sample in the ARG to the H1 or H2 group if it carried the H1 or H2 allele, respectively, at all 15 retained marker sites. We computed cross-coalescence rates between the H1 and H2 groups using the pair_coalescence_rates function in tskit, with 200 evenly spaced, non-overlapping genomic windows from ∼45.6–46.2 Mb and 50 log-spaced time windows from approximately 10^3^ to 3 × 10^5^ generations ago.

Relate-based^37^ upper-bound estimates for the age of the inversion by genomic region were obtained from https://github.com/a-ignatieva/dolores-paper/blob/main/1KGP/data, as published by Ignatieva et al.^34^ We lifted over genomic region coordinates from hg19 to hg38 using liftover^118^ (v1.3.2) with a chain file from https://hgdownload.soe.ucsc.edu/goldenPath/hg19/liftOver/, determining that the total region used for inversion age inference was from ∼45.6–46.2 Mb. The results are compared with tsinfer+tsdate estimates in Fig. S7.

### 11 Comparison to published allele age estimates

To compare allele age estimates produced by tsinfer+tsdate on real data with those of other methods, we assembled a dataset of four previously published estimates for 1KGP data. We developed a Snakemake^119^ pipeline to run tsdate on chromosome 20 of the 1KGP ARGs (Methods §7) and to perform the necessary data manipulation over the existing datasets. We ran tsdate using a mutation rate of $\mu =1.29\times 10^{-8}$.

We obtained allele age estimates from four external methods (Relate, CoalNN, SINGER, and GEVA) from publicly available resources. We downloaded Relate^37^ data from https://zenodo.org/records/3234689, where results are provided separately for each population in the 1KGP; we calculated the age of each mutation as a population size weighted average of the branch midpoint estimates for each population. Site positions were lifted over from hg19 to hg38 using liftover^118^ (version 1.3.2) with a chain file obtained from https://hgdownload.soe.ucsc.edu/goldenPath/hg19/liftOver/. We obtained CoalNN^18^ allele age data for each super population in the 1KGP from https://github.com/PalamaraLab/coalNN_data/releases/tag/v1.0; we applied the same population size weighted averaging approach across super populations to obtain a single estimate per mutation. We downloaded ARGs estimated from 200 African individuals by SINGER^31^ from https://zenodo.org/records/10467509. The dataset consists of 100 ARGs sampled from the posterior, and we extraced allele ages from the first sample. Mean allele ages from GEVA^17^ were downloaded from https://human.genome.dating/, and site positions were lifted over from hg19 to hg38 using the same procedure as for Relate.

After aggregating allele age estimates, we identified a subset of 162,556 sites that were present in all datasets and had consistent derived and ancestral allele assignments. No allele-based filtering was applied to SINGER because nucleotide alleles are not specified in the ARG; ancestral and derived alleles are simply encoded as 0 and 1, respectively. The results are shown in Fig. S10.

### 12 Simulating sampling bias

To perform simulations modelling the sampling bias in the GEL dataset we used stdpopsim^84^ (version 0.2.1) to run forward-time SLiM^120, 121^ (version 4.3) simulations of a 40Mb segment of the human genome, chosen to match the longest inference region (17q4, Tab. S2), and using the HapMapII recombination map.^105^ We imposed selection by simulating neutral and deleterious mutations, applying the Mixed_K23 distribution of fitness effects (DFE)^122^ to exonic regions within the chosen segment, and used the OutOfAfrica_3G09 model,^108^ which describes a history in which European, East Asian, and African populations evolved separately with limited migration over the past 30–60,000 years.

For balanced sampling, we took an equal number of individuals from each simulated population; in the unbalanced case we sampled from these populations in a ratio of 95:1:5, which approximately matches sampling proportions in the GEL dataset. Note that despite the simulation terminating with a global population of only ∼100,000 individuals, the majority of variants still have a DAF < 0.1% (with 76% of variants counted as ultra-rare in the unbalanced case versus 78% in the balanced case). We then ran tsinfer+tsdate on the simulated data, using default parameters.

Mutation ages can only be estimated on the basis of node times above and below a mutation. For a fair comparison, we therefore set the “true” age of each mutation as the midpoint of the simulated dates of its child and parent nodes, comparing this to the inferred midpoint age taken from the topologically unconstrained posterior mean node times provided by tsdate. Mutation ages were only used from sites without recurrent mutations (corresponding to 98.2% or 1,419,535 sites in the simulation with balanced sampling and 98.7% or 1,000,412 sites in the simulation with unbalanced sampling).

The results are shown in Fig S11, and additional analysis is provided in SI §9.

### 13 Distributed ARG inference

Briefly, tsinfer operates in three phases: first, we generate putative ancestral haplotypes from the genotype matrix using heuristics; then we infer how ancestors descend from older ancestors using the Li and Stephens^123^ (LS) Hidden Markov Model (HMM); then we infer how samples descend from the inferred ancestors, using the same LS HMM machinery. Making inference of the large contiguous ARGs fundamental to this study feasible with the computing resources available required two key updates to tsinfer.

A significant bottleneck in earlier versions of tsinfer occurs during the second phase, in which we infer genetic inheritance among inferred ancestors. Groups of ancestors for which the LS HMM was conducted in parallel were determined only by their temporal ordering. This resulted in many small sets of ancestors, greatly limiting parallelism. As ancestors that do not overlap genomically are independent and can be placed in the same matching group this approach is too conservative. Tsinfer version 0.4 uses a linesweep algorithm to detect overlaps, encoding the result in a directed acyclic graph that represents the minimal constraints on parallelism. Parallel ancestor groups are then formed by progressively removing ancestors with no inbound edges until no ancestors remain.

The second key advance in tsinfer version 0.4 is to allow matching under the LS HMM for these groups of independent ancestors (and samples) to be distributed over a cluster. The overall inference job is arranged into a directed graph of tasks which output either partial HMM results or intermediate tree sequence files. The core count and memory requirements for each job are dynamically set from the number and length of ancestors in each group, allowing high cluster utilisation. In addition to enabling greater parallelism, this architecture also provides resilience to the failures that are inevitable in a busy compute cluster, along with checkpointing and mid-run analysis and monitoring. We developed a Snakemake^119^ pipeline to fully orchestrate this process, allowing large-scale tsinfer inference to be performed on a range of job schedulers.

### 14 Genomics England dataset

We inferred ARGs for Genomics England’s aggv2 dataset. This dataset contains 722 million variants for 78,195 rare disease and cancer participants recruited as part of the 100,000 Genomes Project.^19, 124^ 69 of these participants had withdrawn consent for the use of their data in research as per the 100,000 Genomes Project main programme release 17 and were therefore removed from consideration. We excluded a further 30,591 individuals who were identified as parents of other participants and phased by direct transmission information^54^ for two reasons. Firstly, tsinfer does not currently support the use of pedigree information to enforce ancestral relationships between individuals, meaning that the haploid genomes of parents and children would always be contemporaries in the ARG. Secondly, the probands in each pedigree are of primary interest for genetic diagnosis purposes, such that removing them in favour of their parents would limit the clinical applications of inferred mutation ages. The remaining subset of 47,535 putatively unrelated individuals was used for ARG inference.

We converted the aggv2 VCF data to VCF-Zarr format using bio2zarr (version 0.0.8).^111^ We then filtered as above to 47,535 diploid samples, and added ancestral state information.^112^ Sites were then subject to a series of quality control filters which removed sites at duplicated positions, non-SNP variants, non-biallelic sites, and sites with either missing or low quality ancestral state assignments. Additionally, we implemented allele frequency-based filtering, requiring a minimum of 2 derived and 3 ancestral alleles at each site. To avoid problematic flanking regions we applied a sliding window approach with a window size of 100,000 base pairs, requiring that a window-average density of 50 sites per kilobase needed to be met before sites would be used from the 5’ and 3’ ends. We then performed distributed ARG inference using tsinfer, with ancestor truncation disabled, path compression enabled and with a mismatch ratio of zero (ensuring that there is one mutation per site).^38, 41^ We then fully simplified the resulting tree sequences, and used tsdate (version 0.2.1) to split disjoint nodes and then assign ages to nodes and mutations using the variational gamma method with a SNP mutation rate of 1.29 × 10^−8^ per base pair per generation.^82, 125, 126^

As singleton variants were excluded from the phased aggv2, we extracted all biallelic, singleton SNPs and the corresponding individual in which they occur from the unphased aggv2 VCF files using bcftools v1.12, applying the same site-level quality control filters as used by Shi et al.^54^ to construct the phased dataset. We then appended unphased singleton mutations in their derived state to the terminal branches. We re-ran tsdate (version 0.2.1) to phase singleton mutations and date the updated tree sequences using the same parameters as specified above. Allele ages were taken as the posterior mean times inferred using the variational gamma method.

We applied this pipeline to the 15 genome regions detailed in Tab. S2. Statistics for the resulting 15 ARGs are listed in Tab. S4 and the CPU resources required detailed in Tab. S3. The combined dataset of 11,445,983 sites used for primary inference with tsinfer and the 11,778,293 singletons (23 could not be dated due to numerical instability during posterior rescaling) added by tsdate yielded a final dataset of 23,224,276 allele ages.

### 15 Annotation of dated mutations

To characterize how estimated mutation ages vary across diverse genomic contexts, we annotated all variants in the GEL dated ARGs using multiple external resources.

Publicly available ancestry-stratified allele frequencies were obtained from gnomAD v4.1 (genomes and exomes; https://gnomad.broadinstitute.org/data#v4) and GenomeAsia100k^60^ (genomes; https://browser.genomeasia100k.org/#tid=download) as composite VCF files. For each variant overlapping the GEL ARG, alleles were polarised according to the ancestral and derived states in the ARG. Where the external datasets reported the opposite reference/alternate encoding, frequencies were flipped accordingly to yield derived allele frequencies consistent with the GEL ancestral polarisation.

Variant consequences within canonical gene transcripts were predicted using ENSEMBL VEP^127^ v111. AlphaMissense^65^ scores for all missense variants in canonical transcripts were extracted from public Google Cloud buckets (https://console.cloud.google.com/storage/browser/_details/dm_alphamissense/AlphaMissense_hg38.tsv.gz) and, following AlphaMissense nomenclature, classified as ‘likely benign’, ‘ambiguous’, or ‘likely pathogenic’ using default thresholds. Variants were also annotated with PrimateAI-3D^66^ scores downloaded from https://primad.basespace.illumina.com/download, under controlled access for research-only use. Scores > 0.8 were classified as ‘likely pathogenic’, < 0.60 as ‘likely benign’ with all others classified as ‘ambiguous’.

Gene-specific modes of disease inheritance were extracted from 450 virtual gene panels in PanelApp^67^ (https://panelapp.genomicsengland.co.uk/panels/518/, accessed 11 November 2024), restricted to clinical-grade ‘Green’ genes (confidence = 3). Missense variants were classified as ‘recessive’ if the gene was annotated as biallelic in every panel, and ‘dominant’ if annotated as monoallelic in every panel. Genes with multiple or conflicting annotations (both recessive and dominant possible) were excluded. Genes not present in any panel were labelled ‘none’.

Genomic constraint z-scores for chromosome 17, stratified into 1Kb regions, were obtained using the gnocchi method^55^ based on the gnomADv3 sequencing dataset (https://storage.googleapis.com/gcp-public-data--gnomad/release/3.1/secondary_analyses/genomic_constraint/constraint_z_genome_1kb.qc.download.txt.gz). Only scores within quality-controlled regions were used. Variants were assigned constraint classes between < −4 (least constrained) and > 4 (most constrained) if they overlapped a scored region.

### 16 PCA group assignment

Individuals in the GEL dataset were assigned continental group labels according to their genetic distance in PC space to a set of nine reference groups released as part of gnomADv4.1.^55^ Following the gnomAD protocol, the pc_project function from Hail^128^ was used to project sample genotypes and reference group allele frequencies onto the top 20 gnomADv4.1 PC axes (gs://gcp-public-data--gnomad/release/4.0/pca/gnomad.v4.0.pca_loadings.ht). The assign_population_pcs function from the gnomAD Hail utilities was then used to predict group assignments using the trained sklearn ONNX random forest model, with a minimum assignment probability threshold of 0.8. Individuals with a fitted probability of < 0.8 to all reference groups were labelled as “Remaining individuals”. We refer to the groupings of individuals as “PCA groups” throughout.

### 17 Individual vs all coalescence counts

To quantify how uneven ancestry representation in the GEL dataset influences the density and timing of genealogical coalescences we randomly subsampled up to 500 focal individuals per PCA group (or included all individuals when n<500). For each focal individual, coalescence events were counted against all other individuals within the largest contiguous inferred ARG segment (17q4; Tab. S2). Coalescence counts were computed using tskit’s pair_coalescencecounts in evenly spaced, logarithmically scaled time windows. Results are shown in Fig. S20.

### 18 Cryptic relatives in the GEL dataset

Although parents from duo and trio families within the broader aggV2 cohort were excluded from ARG inference, singleton variant counts remain highly sensitive to the presence of close biological relatives. To mitigate this effect, we used the genetically inferred kinship matrix previously generated by the Genomics England bioinformatics team (https://re-docs.genomicsengland.co.uk/principal_components/) to identify cryptic relatedness. From the total cohort of 47,535 individuals in the ARG, we excluded 4,849 with at least one genetically inferred < 3^rd^ degree relative from all analyses involving singleton variants.

### 19 Z-scoring allele ages within allele count bins

Allele age is correlated with allele frequency, with younger alleles generally occurring at lower frequencies (e.g., Fig. S17). To control for this relationship, we normalized allele ages by z-scoring within discrete derived allele count (DAC) bins. For each allele i with estimated age $t_{i}$ and DAC bin g(i), we applied a log_10_ transformation followed by within-bin standardization:

$$z_{i}=\frac{\log_{10}\left(t_{i}\right)-\mu_{g(i)}}{\sigma_{g(i)}}$$

where $\mu_{g(i)}$ and $\sigma_{g(i)}$ represent the mean and standard deviation of ${\text{log}}_{10}(t)$ within DAC bin g(i). This yields a z-score $z_{i}$ that reflects how old or young an allele is relative to others of the same frequency, enabling frequency-controlled comparisons across annotations and individuals.

### 20 Quantile-quantile (QQ) analysis

To compare missense allele age distributions stratified by AlphaMissense or PrimateAI-3D pathogenicity class or PanelApp mode of inheritance, 100 samples from the gamma posterior of each variant were pooled to compute quantiles for each category of variant. For each of Figs. 4a.i and 4b.i, a “control” class is chosen, and then for each percentile, the ratio of that percentile in the comparison class to the value in the control class is shown, plotted against the percentile in the control class. In other words, for each value of 0<p<1, let x(p) and y(p) be the p-th quantiles of the “likely benign” and “likely pathogenic” age distributions, respectively; then each point on the red curve in Fig. 4a.i is of the form (x(p),y(p)/x(p)), for some p. For each non-control group, this procedure was repeated 100 times using bootstrap sampling with replacement to generate 95% confidence intervals in QQ plots.

### 21 Logistic regression analysis

To quantify allele enrichment within frequency-controlled allele age z-score bins (Methods §19) we used logistic regression to compare the frequency of variants assigned to each annotation class against all other variants with valid annotations. For AlphaMissense, PrimateAI-3D and PanelApp mode of inheritance classes, comparisons were restricted to missense variants in the GEL dataset across all contigs with assigned class labels. For constraint, comparisons were made using *all* variants within regions with an annotated constraint level on chromosome 17.

### 22 Clinically classified variants

Variants in the GEL ARG were annotated with clinical classifications based on the ACMG/AMP framework.^129^ Classifications were obtained from ClinVar^130^ (January 2024 release; https://ftp.ncbi.nlm.nih.gov/pub/clinvar/vcf_GRCh38/weekly/clinvar_20240107.vcf.gz) and Genomics England’s internal clinical scientist rare diseases outcomes questionnaire (main programme release v19; https://re-docs.genomicsengland.co.uk/exit_questionnaire/). Variants with discordant classifications, either within or across sources, were excluded prior to downstream analyses. Pathogenic and likely pathogenic variants were grouped as P/LP, and benign and likely benign variants were grouped as B/LB. Clinically classified variants (DAC > 1) whose child nodes showed extreme uncertainties in ages were excluded.

### 23 ARG-informed heuristic to identify likely recurrent mutations

Recurrent mutations—independent mutational events at the same genomic site—violate a core assumption of the ARG inference method we used that each mutation originates once and is inherited along a single ancestral branch. Such violations can distort local genealogies and bias allele age estimates, and hence pose a particular challenge when analysing individual variants. To mitigate the effect of recurrent mutations biasing our analysis of clinically classified variants, we devised a heuritic identify mutations more likely to be recurrent by examining the local genealogical structure of each ultra-rare clinically classified variant with DAC > 1 and DAF < 0.1%.

For each variant, we computed the edge span (the genomic interval over which the ancestral branch carrying the mutation is inherited) and the local identity-by-descent (IBD) span (the region where all carriers share a most recent common ancestor at the node immediately below the mutation).

Under the assumption of a single origin, the mutation-bearing edge would typically fall within a broader region of shared ancestry. In contrast, recurrent or misinferred mutations are more likely to generate short, isolated branches or apparent discontinuities in the inferred ARG. Accordingly, we flagged mutations as more likely to be recurrent if (a) the mutation coincided with the boundary of its edge span, suggesting the introduction of an artificial recombination breakpoint during inference to accommodate inconsistent mutation patterns; or (b) the edge span was shorter than or equal to the local IBD span, indicating genealogical inconsistency with the surrounding ancestry.

Following this, we annotated each mutation as a CpG>TpG transition (or its reverse complement) using the ancestral reference sequence. We first identified CpG dinucleotides by scanning the ancestral FASTA sequence for consecutive “CG” bases and storing their genomic coordinates. Mutations were then labelled as CpG>TpG transitions if they occurred at one of these CpG dinucleotide positions and involved a C to T or G to A substitution.

We then evaluated our recurrence heuristic across DAC bins and by mutation type. As expected, mutations classified as CpG>TpG transitions—known hotspots for recurrent events due to methylation-mediated deamination—were more frequently flagged as likely recurrent than other mutation types (Fig. S24) We also observed an increasing proportion of flagged variants at higher DAC values, consistent with the expectation that variants with more derived alleles have a higher likelihood of including at least one recurrent event.

## Supplementary Material

Supplement 1
