# Supplementary material for: Tracing the evolutionary histories of ultra-rare variants using variational dating of large ancestral recombination graphs
Source: bioRxiv. 2026 Jan 12:2026.01.07.698223. Preprint. [Version 1] doi: 10.64898/2026.01.07.698223 (PMC12871112; doi:10.64898/2026.01.07.698223)
Supplement: Supplement 1 [file NIHPP2026.01.07.698223v1-supplement-1.pdf]

bioRxiv preprint doi: https://doi.org/10.64898/2026.01.07.698223; this version posted January 12, 2026. The copyright holder for this preprint (which was not certified by peer review) is the author/funder, who has granted bioRxiv a license to display the preprint in perpetuity. It is made available under aCC-BY 4.0 International license.

Supplementary Information

This section contains the supplementary information, which we integrate into the main document for ease of cross referencing during review.

1 Expectation propagation update

Here we derive the moments used in the expectation propagation update described in Methods §1. Recall that the "surrogate" distribution for which we wish to compute moments has the unnormalized form,

$$\begin{aligned} &q_{\theta_i-\theta_{i,ij}}(t_i)q_{\theta_j-\theta_{j,ij}}(t_j)p_{ij}(y_{ij}|t_i-t_j) \\ &\quad\propto t_i^{\tilde{\alpha}_i-1}t_j^{\tilde{\alpha}_j-1}(t_i-t_j)^{y_{ij}}e^{-\tilde{\beta}_i t_i-\tilde{\beta}_j t_j-\mu s_{ij}(t_i-t_j)}. \end{aligned}$$

Then, if we define

$$Z_{ij}(a_i,a_j)=\int_0^\infty\int_0^{t_i} t_i^{a_i-1}t_j^{a_j-1}(t_i-t_j)^{y_{ij}}e^{-\tilde{\beta}_i t_i-\tilde{\beta}_j t_j-\mu s_{ij}(t_i-t_j)}dt_j dt_i,$$

then we can obtain the moments through

$$\mathbb{E}[t_i^k t_j^\ell]=\frac{Z_{ij}(\tilde{\alpha}_i+k,\tilde{\alpha}_j+\ell)}{Z_{ij}(\tilde{\alpha}_i,\tilde{\alpha}_j)}.$$

To evaluate this, rewrite the integral with $T=t_i$ and $uT=t_j$, so that

$$\begin{aligned} Z_{ij}(a_i,a_j)&=\int_0^1\int_0^\infty T^{a_i-1}(uT)^{a_j-1}(T(1-u))^{y_{ij}}e^{-\tilde{\beta}_i T-\tilde{\beta}_j uT-\mu s_{ij}(1-u)T}TdTdu \\ &=\int_0^1\left(\int_0^\infty T^{a_i+a_j+y_{ij}-1}e^{-\{\tilde{\beta}_i+\mu s_{ij}+(\tilde{\beta}_j-\mu s_{ij})u\}T}dT\right)u^{a_j-1}(1-u)^{y_{ij}}du \\ &=\int_0^1\frac{\Gamma(a_i+a_j+y_{ij})}{(\tilde{\beta}_i+\mu s_{ij}+(\tilde{\beta}_j-\mu s_{ij})u)^{a_i+a_j+y_{ij}}}u^{a_j-1}(1-u)^{y_{ij}}du. \end{aligned}$$

Next we use the fact that (ref[131] §3.197.3)

$$\int_0^1 x^{b-1}(1-x)^{c-b-1}(1-zx)^{-a}dx=B(b,c-b)\,{}_2F_1(a,b;c;z)\qquad\text{for } c>b>0,\ |z|<1,$$

where B is the beta function and ${}_2F_1$ is the Gaussian hypergeometric function. With $b=a_j$, $c=1+a_j+y_{ij}$, $a=a_i+a_j+y_{ij}$, and $z=(\tilde{\beta}_j-\mu s_{ij})/(\tilde{\beta}_i+\mu s_{ij})$, this is

$$Z_{ij}(a_i,a_j)=\frac{B(y_{ij}+1,a_j)\Gamma(a_i+a_j+y_{ij})}{(\mu s_{ij}+\beta_i)^{a_i+a_j+y_{ij}}}\,{}_2F_1\left(\begin{matrix}a_j,a_i+a_j+y_{ij}\\ a_j+y_{ij}+1\end{matrix};\frac{\mu s_{ij}-\beta_j}{\mu s_{ij}+\beta_i}\right). \tag{11}$$

If node j is a contemporary sample ($t_j=0$) this simplifies to

$$Z_{ij}(a_i)=\frac{\Gamma(a_i+y_{ij})}{(\mu s_{ij}+\tilde{\beta}_i)^{a_i+y_{ij}}}.$$

bioRxiv preprint doi: https://doi.org/10.64898/2026.01.07.698223; this version posted January 12, 2026. The copyright holder for this preprint (which was not certified by peer review) is the author/funder, who has granted bioRxiv a license to display the preprint in perpetuity. It is made available under aCC-BY 4.0 International license.

Defining

$$F_{in} = {}_2F_1\left(\begin{matrix}\tilde{\alpha}_j, \tilde{\alpha}_i + \tilde{\alpha}_j + y_{ij} + n \\ \tilde{\alpha}_j + y_{ij} + 1\end{matrix}; \frac{\mu s_{ij} - \tilde{\beta}_j}{\mu s_{ij} + \tilde{\beta}_i}\right)$$
$$F_{jn} = {}_2F_1\left(\begin{matrix}\tilde{\alpha}_j + n, \tilde{\alpha}_i + \tilde{\alpha}_j + y_{ij} + n \\ \tilde{\alpha}_j + y_{ij} + n + 1\end{matrix}; \frac{\mu s_{ij} - \tilde{\beta}_j}{\mu s_{ij} + \tilde{\beta}_i}\right),$$

we can thus write the first two moments as

$$\mathbb{E}[t_i] = \frac{\tilde{\alpha}_i + \tilde{\alpha}_j + y_{ij}}{\mu s_{ij} + \tilde{\beta}_i}\frac{F_{i1}}{F_{i0}}$$
$$\mathbb{E}[t_i^2] = \frac{(\tilde{\alpha}_i + \tilde{\alpha}_j + y_{ij})(\tilde{\alpha}_i + \tilde{\alpha}_j + y_{ij} + 1)}{(\mu s_{ij} + \tilde{\beta}_i)^2}\frac{F_{i2}}{F_{i0}}$$
$$\mathbb{E}[t_j] = \frac{\tilde{\alpha}_j(\tilde{\alpha}_i + \tilde{\alpha}_j + y_{ij})}{(\mu s_{ij} + \tilde{\beta}_i)(\tilde{\alpha}_j + y_{ij} + 1)}\frac{F_{j1}}{F_{j0}}$$
$$\mathbb{E}[t_j^2] = \frac{\tilde{\alpha}_j(\tilde{\alpha}_j + 1)(\tilde{\alpha}_i + \tilde{\alpha}_j + y_{ij})(\tilde{\alpha}_i + \tilde{\alpha}_j + y_{ij} + 1)}{(\mu s_{ij} + \tilde{\beta}_i)^2(\tilde{\alpha}_j + y_{ij} + 1)(\tilde{\alpha}_j + y_{ij} + 2)}\frac{F_{j2}}{F_{j0}}.$$

Or, when node j is a contemporary sample ($t_j = 0$),

$$\mathbb{E}[t_i] = (\tilde{\alpha}_i + y_{ij})(\mu s_{ij} + \tilde{\beta}_i)^{-1}$$
$$\mathbb{E}[t_i^2] = (\tilde{\alpha}_i + y_{ij})(\tilde{\alpha}_i + y_{ij} + 1)(\mu s_{ij} + \tilde{\beta}_i)^{-2}.$$

The updated natural parameters are obtained from the moments by

$$\theta_i' = \left[\frac{\mathbb{E}[t_i]^2}{\mathbb{E}[t_i^2] - \mathbb{E}[t_i]^2} - 1, -\frac{\mathbb{E}[t_i]}{\mathbb{E}[t_i^2] - \mathbb{E}[t_i]^2}\right].$$

As a final note: the computational bottleneck in the fitting procedure will be computing values of ${}_2F_1$, so it is helpful to reduce the number of distinct values we need to compute. Using Gauss's contiguous relation that $z(ab/c){}_2F_1(a+1, b+1; c+1; z) = b({}_2F_1(a, b+1; c+1; z) - {}_2F_1(a, b; c; z))$, we can write F_{i1}/F_{i0} in terms of F_{j1}/F_{j0}, and similarly reduce F_{i2}/F_{i0}, obtaining that

$$\mathbb{E}[t_i] = \mathbb{E}[t_j]\frac{\mu s_{ij} - \tilde{\beta}_j}{\mu s_{ij} + \tilde{\beta}_i} + \frac{\tilde{\alpha}_i + \tilde{\alpha}_j + y_{ij}}{\mu s_{ij} + \tilde{\beta}_i}$$
$$\mathbb{E}[t_i^2] = \mathbb{E}[t_j^2]\left(\frac{\mu s_{ij} - \tilde{\beta}_j}{\mu s_{ij} + \tilde{\beta}_i}\right)^2 + \frac{\tilde{\alpha}_i + \tilde{\alpha}_j + y_{ij} + 1}{\mu s_{ij} + \tilde{\beta}_i}\left(2\mathbb{E}[t_i] - \frac{\tilde{\alpha}_i + \tilde{\alpha}_j + y_{ij}}{\mu s_{ij} + \tilde{\beta}_i}\right).$$

2 Numerically stable approximations of ${}_2F_1$

The Gaussian hypergeometric function ${}_2F_1$ can be difficult to compute in a numerically stable fashion for arbitrary parameter regimes without relying on multiprecision floating point arithmetic, due to catastrophic cancellation between alternating terms in the defining hypergeometric series.

A numerically stable alternative is to approximate ${}_2F_1$ via Laplace's method, using the scheme in[98] (derived here for completeness). The idea is to use Euler's integral identity

$${}_2F_1(a, b; c; z) = \int_0^1 \frac{\exp\{a\log x + (c-a)\log(1-x) - b\log(1-zx)\}}{x(1-x)B(a, c-a)}dx$$

bioRxiv preprint doi: https://doi.org/10.64898/2026.01.07.698223; this version posted January 12, 2026. The copyright holder for this preprint (which was not certified by peer review) is the author/funder, who has granted bioRxiv a license to display the preprint in perpetuity. It is made available under aCC-BY 4.0 International license.

and change variables such that the transformed integrand is well-approximated by a Gaussian function. Note that $f(x) = -a \log x - (c-a)\log(1-x) + b\log(1-zx)$ has a minimum in $(0,1)$ given by

$$\hat{x} = 2a(\sqrt{t^2 - 4az(c-b)} - t)^{-1}, \quad \text{where } t = z(b-a) - c$$

provided $c > a > 0$ and $z > 0$. The first condition will always be satisfied in our application; and if $z < 0$ then the Pfaff transform ${}_2F_1(a,b;c;z) = (1-z)^{-b}{}_2F_1(c-a,b;c;z(z-1)^{-1})$ is used instead.

Making the logit change of variables by setting $x' = g(x) = \log(x/(1-x))$ (and so $dx' = x^{-1}(1-x)^{-1}dx$ and $x' = g(x) = \log x - \log(1-x)$, $x = g^{-1}(x') = e^{x'}/(1+e^{x'})$) and taking a second-order Taylor expansion of $f(g^{-1}(x'))$ around $g(\hat{x})$ leads to:

$$\begin{aligned}
{}_2F_1(a,b;c;z) &= B(a,c-a)^{-1}\int_{-\infty}^{\infty} \exp\{-f(g^{-1}(x'))\}dx' \\
&\approx B(a,c-a)^{-1}\int_{-\infty}^{\infty} \exp\left\{-f(\hat{x}) - (x'-g(\hat{x}))^2 \frac{f''(\hat{x})}{2g'(\hat{x})^2}\right\} dx' \\
&= \frac{g'(\hat{x})\exp\{-f(\hat{x})\}}{B(a,c-a)}\left(\frac{2\pi}{f''(\hat{x})}\right)^{\frac{1}{2}} \\
&= \frac{\hat{x}^{a-1}(1-\hat{x})^{c-a-1}}{B(a,c-a)(1-z\hat{x})^b}\left(\frac{2\pi}{a\hat{x}^{-2} + (c-a)(1-\hat{x})^{-2} - bz^2(1-z\hat{x})^{-2}}\right)^{\frac{1}{2}},
\end{aligned} \tag{12}$$

where the first-order term has disappeared from the Taylor approximation on the second line because $f'(\hat{x}) = 0$. Finally,[98] suggest using the identity ${}_2F_1(a,b;c;0) = 1$ to calibrate the approximation, via

$$\begin{aligned}
{}_2F_1(a,b;c;z) &= \frac{{}_2F_1(a,b;c;z)}{{}_2F_1(a,b;c;0)} \\
&\approx \frac{c^{c-1/2}}{a^a(c-a)^{c-a}}\frac{\hat{x}^a(1-\hat{x})^{c-a}}{(1-z\hat{x})^b}\left(\frac{\hat{x}^2}{a} + \frac{(1-\hat{x})^2}{c-a} - \frac{bz^2\hat{x}^2(1-\hat{x})^2}{a(c-a)(1-z\hat{x})^2}\right)^{-\frac{1}{2}},
\end{aligned}$$

which follows from applying (12) separately to the numerator and denominator and simplifying the result.

3 Non-contemporary samples and internal constraints

In previous sections we have assumed that sample nodes are contemporary (fixed to time zero), in which case the expectation propagation surrogate for singleton edges reduces to a Gamma distribution and moments follow directly. However, if an edge ij has one end fixed to a particular nonzero time, the solution is more complicated. As above we used the hypergeometric function ${}_2F_1$, here we will encounter the hypergeometric functions of Tricomi and Kummer, U and M, with integral representations

$$\begin{aligned}
U(a,b;z) &= \int_0^{\infty} \frac{\exp\{-zu + a\log u + (b-a)\log(1+u)\}}{u\Gamma(a)}du \\
M(a,b;z) &= \int_0^{1} \frac{\exp\{-zv + a\log v + (b-a)\log(1-v)\}}{x(1-v)B(a,b-a)}dv.
\end{aligned}$$

bioRxiv preprint doi: https://doi.org/10.64898/2026.01.07.698223; this version posted January 12, 2026. The copyright holder for this preprint (which was not certified by peer review) is the author/funder, who has granted bioRxiv a license to display the preprint in perpetuity. It is made available under aCC-BY 4.0 International license.

Following the notation of SI §1 for the edge ij, define for later use

$$\begin{aligned} U_n &= U(y_{ij} + n + 1, \tilde{\alpha}_i + y_{ij} + n + 1; (\mu s_{ij} + \tilde{\beta}_i)t_j) \\ M_n &= M(\tilde{\alpha}_k + n, \tilde{\alpha}_k + y_{jk} + n + 1; (\mu s_{jk} - \tilde{\beta}_k)t_j). \end{aligned}$$

First suppose the child j has fixed time t_j. Then the normalizer analogous to Z_{ij} in (11) is

$$\begin{aligned} Z_{ij}(a_i) &= \int_{t_j}^{\infty} t_i^{a_i - 1}(t_i - t_j)^{y_{ij}} e^{-\tilde{\beta}_i t_i - \mu s_{ij}(t_i - t_j)} dt_i \\ &= \frac{\Gamma(y_{ij}+1) t_j^{a_i + y_{ij}}}{\tilde{\beta}_i t_j} U_0, \end{aligned}$$

and so

$$\begin{aligned} E[t_i] &= t_j \left(1 + (y_{ij}+1)\frac{U_1}{U_0}\right) \\ E[t_i^2] &= t_j^2 \left(1 + 2(y_{ij}+1)\frac{U_1}{U_0} + (y_{ij}+1)(y_{ij}+2)\frac{U_2}{U_0}\right). \end{aligned}$$

Now suppose that the edge is jk with fixed time t_j (where j is now the parent). In this case, the normalizer is

$$\begin{aligned} Z_{jk}(a_k) &= \int_{0}^{t_j} t_k^{a_k - 1}(t_j - t_k)^{y_{jk}} e^{-\tilde{\beta}_k t_k - \mu s_{jk}(t_j - t_k)} dt_k \\ &= \frac{B(a_k, y_{jk}+1) t_j^{a_k + y_{jk}}}{\mu s_{jk} t_j} M_0, \end{aligned}$$

and so

$$\begin{aligned} E[t_k] &= t_j \frac{\tilde{\alpha}_k}{\tilde{\alpha}_k + y_{jk} + 1}\frac{M_1}{M_0} \\ E[t_k^2] &= t_j^2 \frac{\tilde{\alpha}_k(\tilde{\alpha}_k+1)}{(\tilde{\alpha}_k + y_{jk} + 1)(\tilde{\alpha}_k + y_{jk} + 2)}\frac{M_2}{M_0}. \end{aligned}$$

Calculating these moments in practice requires evaluation of the Tricomi hypergeometric function $U(a, b; z)$ or the Kummer hypergeometric function $M(a, b; z)$. As for ${}_2F_1$, direct evaluation of the defining hypergeometric series can suffer from numerical instability under certain parameter regimes. Given the integral identities,

$$\begin{aligned} U(a, b; z) &= \int_0^{\infty} \frac{\exp\{-zu + a\log u + (b-a)\log(1+u)\}}{u\Gamma(a)} du \\ M(a, b; z) &= \int_0^{1} \frac{\exp\{-zv + a\log v + (b-a)\log(1-v)\}}{x(1-v)B(a, b-a)} dv \end{aligned}$$

changing variables to $du' = u^{-1}du$ and $dv' = v^{-1}(1-v)^{-1}dv$, then using Laplace's method leads to the numerically stable approximations,

$$\begin{aligned} U(a, b; z) &= \frac{U(a, b; z)}{z^a U(a, a+1; z)} \approx \frac{e^a}{a^a}\left(1 + \frac{(b-a-1)\hat{u}^2}{a(1+\hat{u})^2}\right)^{-\frac{1}{2}} (1+\hat{u})^{b-a-1}\hat{u}^a e^{-z\hat{u}} \\ \hat{u} &= \frac{2a}{\sqrt{(b-z-1)^2 + 4za} - b + z + 1} \end{aligned}$$

bioRxiv preprint doi: https://doi.org/10.64898/2026.01.07.698223; this version posted January 12, 2026. The copyright holder for this preprint (which was not certified by peer review) is the author/funder, who has granted bioRxiv a license to display the preprint in perpetuity. It is made available under aCC-BY 4.0 International license.

and

$$M(a,b;z) = \frac{M(a,b;z)}{M(a,b;0)} \approx \frac{b^b}{a^a(b-a)^{b-a}} \left(\frac{\hat{v}^2 b}{a} + \frac{(1-\hat{v})^2 b}{b-a}\right)^{-\frac{1}{2}} (1-\hat{v})^{b-a}\hat{v}^a e^{z\hat{v}}$$
$$\hat{v} = \frac{2a}{\sqrt{(z-b)^2+4za} - z + b}$$

where we have used the identities $z^a U(a, a+1; z) = 1$ and $M(a,b;0) = 1$ to calibrate the approximations, analogously to SI §2.

4 Mutation ages

Mutations are mapped to edges in the ARG, but we cannot know more precisely where a particular mutation is located in time. Implicit in the Poisson mutation process from Methods §1 is the assumption that mutations are uniformly distributed along the time dimension of an edge. Thus the nth conditional moment of the age of a mutation on edge ij (with parent i, child j) is $\mathbb{E}[t_{ij}^n | t_i, t_j] = (t_i^{n+1} - t_j^{n+1})(t_i - t_j)^{-1}(n+1)^{-1}$. Let $q_{\boldsymbol{\theta}}^{\setminus ij} p_{ij}(\boldsymbol{t})$ be the variational surrogate for edge ij (i.e. the "cavity" distribution for edge ij multiplied by its Poisson likelihood), defined in equation (3). As described previously, the surrogate distribution is globally approximate (in that it replaces the joint distribution of node ages by a factorized variational approximation) but locally exact (in that it models the dependence between the age of parent i and child j that is due to the likelihood of edge ij). Under this approximation, the age t_{ij} of a mutation on the edge has the nth unconditional moment,

$$\mathbb{E}[t_{ij}^n] = \frac{\int \mathbb{E}[t_{ij}^n | t_i, t_j] q_{\boldsymbol{\theta}}^{\setminus ij} p_{ij}(\boldsymbol{t}) d\boldsymbol{t}}{\int q_{\boldsymbol{\theta}}^{\setminus ij} p_{ij}(\boldsymbol{t}) d\boldsymbol{t}}.$$

Using the precise definition of the variational gamma surrogate and following the rationale and notation of SI §1, let

$$F_{nmo} = {}_2F_1 \left(\begin{matrix} \tilde{\alpha}_j + n, \tilde{\alpha}_i + \tilde{\alpha}_j + y_{ij} + m \\ \tilde{\alpha}_j + y_{ij} + o + 1 \end{matrix} ; \frac{\mu s_{ij} - \tilde{\beta}_j}{\mu s_{ij} + \tilde{\beta}_i} \right).$$

Then the first two moments of the mutation's age are,

$$\mathbb{E}[t_{ij}] = \left(1 + \frac{\tilde{\alpha}_j(\mu s_{ij} - \tilde{\beta}_j + 1)}{\tilde{\alpha}_j + y_{ij} + 1} \frac{F_{111}}{F_{000}}\right) \frac{\tilde{\alpha}_i + \tilde{\alpha}_j + y_{ij}}{2(s_{ij}\mu + \tilde{\beta}_i)}$$
$$\mathbb{E}[t_{ij}^2] = \left(\frac{F_{020}}{F_{000}} + \frac{\tilde{\alpha}_j}{\tilde{\alpha}_j + y_{ij} + 1}\left(\frac{F_{121}}{F_{000}} + \frac{\tilde{\alpha}_j + 1}{\tilde{\alpha}_j + y_{ij} + 2}\frac{F_{222}}{F_{000}}\right)\right) \times \frac{(\tilde{\alpha}_i + \tilde{\alpha}_j + y_{ij})(\tilde{\alpha}_i + \tilde{\alpha}_j + y_{ij} + 1)}{3(s_{ij}\mu + \tilde{\beta}_i)^2}$$

and may be used to construct a variational gamma approximation, as was done for node ages.

5 Ambiguous singleton phasing

In diploid data, mutations with frequency one are likely to have ambiguous phase. In this case, the model in SI §1 may be adjusted so as to integrate over both possible phases for each singleton

bioRxiv preprint doi: https://doi.org/10.64898/2026.01.07.698223; this version posted January 12, 2026. The copyright holder for this preprint (which was not certified by peer review) is the author/funder, who has granted bioRxiv a license to display the preprint in perpetuity. It is made available under aCC-BY 4.0 International license.

mutation. This requires splitting the edges above each leaf so that any given individual has at most two immediate ancestors (nodes) over the span of every attached edge. For example, imagine we have a sequence of length L containing a diploid individual where the first haplotype has immediate ancestor i over interval $[0, a)$ and ancestor j over interval $[a, L)$; and similarly the second haplotype has ancestor k over interval $[0, b)$ and ancestor l over interval $[b, L)$. Each of these intervals is represented by an edge in the tree sequence data structure, and in general $a \neq b$; that is, edges can overlap between the two haplotypes. After splitting leaf edges as described above for this example, if $a < b$, the individual is subtended by a pair of edges leading to ancestors i and k over the interval $[0, a)$, a pair leading to j and k over $[a, b)$, and a pair leading to j and l over $[b, L)$.

Consider an individual whose haplotypes are u and v, and let $\mathcal{E}_{uv}$ be the edges that end in u or v (i.e., $\mathcal{E}_{uv} = \{ij \in \mathcal{E} \ : \ j = u \text{ or} j = v\}$). After splitting as described above, these edges come in concurrent pairs of the form (iu, jv), where i and j are the immediate ancestors of the individual on a given genomic segment (and possibly $i = j$). Each pair is associated with an interval of span $s_{iu,jv}$, and these intervals are nonoverlapping. If a singleton mutation over this interval is unphased, then it cannot be assigned to either edge iu or edge jv. If the individual (u, v) is contemporary ($t_u = t_v = 0$) then the total branch area of the edge pair is $s_{iu,jv}(t_i + t_j)$. Assuming all singleton mutations on the edge pair are unphased, the likelihood for the total count $y_{iu,jv}$ of such mutations is

$$p(y_{iu,jv}|t_i + t_j) = \frac{(\mu s_{iu,jv})^{y_{iu,jv}}}{\Gamma(y_{iu,jv} + 1)}(t_i + t_j)^{y_{iu,jv}} \exp\{-\mu s_{iu,jv}(t_i + t_j)\}.$$

Let $\mathcal{I}$ denote the set of individuals, $\mathcal{E}_\mathcal{I} = \bigcup_{uv \in \mathcal{I}} \mathcal{E}_{uv}$ the set of all edges subtended by individuals, and $\mathcal{P}_{uv}$ the set of concurrent edge pairs for individual uv. Then the joint distribution of node ages becomes

$$p(\boldsymbol{t}, \boldsymbol{y}|\boldsymbol{\eta}) = p(\boldsymbol{t}|\boldsymbol{\eta}) \prod_{ij \in \mathcal{E} \setminus \mathcal{P}} p(y_{ij}|t_i - t_j) \prod_{iu,jv \in \mathcal{P}} p(y_{iu,jv}|t_i + t_j),$$

where $\mathcal{P}$ are all split leaf edges and $\mathcal{E} \setminus \mathcal{P}$ are all internal edges (e.g., edges carrying phased, non-singleton mutations).

Following the rationale in SI §1 leads to an expectation propagation update for the ages of the two ancestors t_i and t_j involved in a given singleton edge pair:

$$\begin{aligned}
F_k &= {}_2F_1\left(\begin{matrix}\tilde{\alpha}_j + k, \tilde{\alpha}_i + \tilde{\alpha}_j + y_{iu,jv} + k \\ \tilde{\alpha}_i + \tilde{\alpha}_j + k\end{matrix}; \frac{\mu s_{iu,jv} + \tilde{\beta}_j}{\mu s_{iu,jv} + \tilde{\beta}_i}\right) \\
\mathbb{E}[t_j] &= \frac{\tilde{\alpha}_j(\tilde{\alpha}_i + \tilde{\alpha}_j + y_{iu,jv})}{(\mu s_{iu,jv} + \tilde{\beta}_i)(\tilde{\alpha}_i + \tilde{\alpha}_j)} \frac{F_1}{F_0} \\
\mathbb{E}[t_j^2] &= \frac{\tilde{\alpha}_j(\tilde{\alpha}_j + 1)(\tilde{\alpha}_i + \tilde{\alpha}_j + y_{iu,jv})(\tilde{\alpha}_i + \tilde{\alpha}_j + y_{iu,jv} + 1)}{(\mu s_{iu,jv} + \tilde{\beta}_i)^2(\tilde{\alpha}_i + \tilde{\alpha}_j)(\tilde{\alpha}_i + \tilde{\alpha}_j + 1)} \frac{F_2}{F_0} \\
\mathbb{E}[t_i] &= -\mathbb{E}[t_j]\frac{\mu s_{iu,jv} + \beta_j}{\mu s_{iu,jv} + \beta_i} + \frac{\tilde{\alpha}_i + \tilde{\alpha}_j + y_{iu,jv}}{\mu s_{iu,jv} + \tilde{\beta}_i} \\
\mathbb{E}[t_i^2] &= \mathbb{E}[t_j^2]\left(\frac{\mu s_{iu,jv} + \tilde{\beta}_j}{\mu s_{iu,jv} + \tilde{\beta}_i}\right)^2 + \frac{\tilde{\alpha}_i + \tilde{\alpha}_j + y_{iu,jv} + 1}{\mu s_{iu,jv} + \tilde{\beta}_i}\left(2\mathbb{E}[t_i] + \frac{\tilde{\alpha}_i + \tilde{\alpha}_j + y_{iu,jv}}{\mu s_{iu,jv} + \tilde{\beta}_i}\right),
\end{aligned}$$

assuming these ancestors are distinct ($i \neq j$). If instead a single ancestor is attached to both

bioRxiv preprint doi: https://doi.org/10.64898/2026.01.07.698223; this version posted January 12, 2026. The copyright holder for this preprint (which was not certified by peer review) is the author/funder, who has granted bioRxiv a license to display the preprint in perpetuity. It is made available under aCC-BY 4.0 International license.

edges of the pair (so $i = j$), the update becomes

$$\mathbb{E}[t_i] = (\tilde{\alpha}_i + y_{iu,jv})(\mu s_{iu,jv} + \tilde{\beta}_i)^{-1}$$
$$\mathbb{E}[t_i^2] = (\tilde{\alpha}_i + y_{iu,jv})(\tilde{\alpha}_i + y_{iu,jv} + 1)(\mu s_{iu,jv} + \tilde{\beta}_i)^{-2}$$

because the surrogate reduces to a Gamma distribution.

6 Mixture prior on root ages

In SI §1, we assumed generic gamma priors for particular nodes without specifying how these are determined. Information about the ages of nodes comes from noisy measurements of edge lengths – mutational density – that are propagated up the ARG from a fixed reference point (the sample ages). Typically, the oldest ancestral segments will each have very short spans, because of the cumulative action of recombination. Mutational density is sparse on these ancient haplotypes, and there is relatively little ancestral material that can act as constraint on their ages. As a consequence, the posteriors of the oldest nodes typically have extremely high variance, and this is exacerbated in the variational approximations by the local nature of expectation propagation. Thus, we regularise the ages of "ultimate" roots (the nodes with no parents) via a prior, and use flat priors for all other nodes. This scheme acts as a soft constraint on the maximum height of the ARG. Because the choice of prior introduces another decision for the user with potentially large consequences on the quality of inference, we fit an arbitrarily flexible prior via an Empirical Bayes method.

To this end, we employ a mixture of gamma distributions for a prior on the ages of ultimate roots, with hyperparameters that are estimated during each EP iteration from the current variational approximation. Explicitly, let the hyperparameters $\eta_k = \{\omega_k, \gamma_k, \kappa_k\}$ be the mixture weight, shape, and rate for the kth mixture component. The prior for the ith node is

$$p(t_i|\boldsymbol{\eta}) \propto \sum_{k=1}^{K} \delta_{i,k} \frac{\omega_k \kappa_k^{\gamma_k}}{\Gamma(\gamma_k)} \exp\{(\gamma_k - 1)\log t_i - \kappa_k t_i\},$$

where the node-specific weights $\delta_{i,k}$ are assumed to be specified *a priori*, so that the choice $\delta_{i,k} = 1$ if node i has no parents and $\delta_{i,k} = 0$ otherwise leads to an i.i.d. prior across ultimate roots. Other choices of δ allow stratification of the prior across frequency classes or windows along the genome, or extension of the prior to non-root nodes. As before, the messages due to the prior are removed from the variational approximation to create the cavity $q_{\boldsymbol{\theta}}^{\setminus 0}(\boldsymbol{t}) \propto q_{\boldsymbol{\theta}}(\boldsymbol{t}) / \prod_i q_{i,0}(t_i)$ with natural parameters $\theta_i^{\setminus 0} = \theta_i - \theta_{i,0}$ and then updated by matching moments against the surrogate $q^{\setminus 0}(\boldsymbol{t}) \prod_i p(t_i|\boldsymbol{\eta})$.

We extend this standard EP update with an expectation-maximization (EM) procedure that finds optimal hyperparameters for the surrogate. To perform EM, we augment the model with per-node binary variables $z_{i,k}$ that indicate if node i belongs to mixture component k, so that under this model $\sum_k z_{i,k} = 1$ and $z_{i,k} = 1$ with probability ω_k, and then if $z_{i,k} = 1$ then t_i is drawn from the distribution with parameters (γ_k, κ_k). The joint posterior of $\boldsymbol{t}$ and $\boldsymbol{z}$ is then

$$g(\boldsymbol{t}, \boldsymbol{z}|\boldsymbol{\eta}) \propto q_{\boldsymbol{\theta}}^{\setminus 0}(\boldsymbol{t}) \prod_{k=1}^{K} \prod_{i \in \mathcal{N}} \left(\frac{\delta_{i,k} \omega_k \kappa_k^{\gamma_k}}{\Gamma(\gamma_k)} \exp\{(\gamma_k - 1)\log t_i - \kappa_k t_i\} \right)^{z_{i,k}}.$$

Now our goal is, given a current set of parameters $\eta^{(n)}$, to find $\eta^{(n+1)}$ to maximize the expected value of $\log g$, averaging $\boldsymbol{t}$ and $\boldsymbol{z}$ across the posterior. To do this, let $g_n \equiv g(\boldsymbol{t}, \boldsymbol{z}|\boldsymbol{\eta}^{(n)})$ denote

bioRxiv preprint doi: https://doi.org/10.64898/2026.01.07.698223; this version posted January 12, 2026. The copyright holder for this preprint (which was not certified by peer review) is the author/funder, who has granted bioRxiv a license to display the preprint in perpetuity. It is made available under aCC-BY 4.0 International license.

the augmented surrogate at the nth EM iteration. The expectation step yields the objective function,

$$
\begin{aligned}
Q(\boldsymbol{\eta}^{(n+1)} \parallel \boldsymbol{\eta}^{(n)}) &= \mathbb{E}_{g_n}[\log g_{n+1}] \\
&= C + \sum_{k=1}^{K} (\log \omega_k^{(n+1)} - \log \Gamma(\gamma_k^{(n+1)}) + \gamma_k^{(n+1)} \log \kappa_k^{(n+1)}) \sum_{i \in \mathcal{N}} \mathbb{E}_{g_n}[z_{i,k}] \\
&\quad + \sum_{k=1}^{K} (\gamma_k^{(n+1)} - 1) \sum_{i \in \mathcal{N}} \mathbb{E}_{g_n}[z_{i,k} \log t_i] - \sum_{k=1}^{K} \kappa_k^{(n+1)} \sum_{i \in \mathcal{N}} \mathbb{E}_{g_n}[z_{i,k} t_i]
\end{aligned}
$$

where the necessary expectations involve standard manipulations of gamma distributions,

$$
\begin{aligned}
\mathbb{E}_{g_n}[z_{i,k}] &= \frac{\delta_{i,k} \omega_k^{(n)} \Gamma(\gamma_k^{(n)})^{-1} (\kappa_k^{(n)})^{\gamma_k^{(n)}} \Gamma(\gamma_k^{(n)} + \alpha_i^{\setminus 0} - 1)(\kappa_k^{(n)} + \beta_i^{\setminus 0})^{-\gamma_k^{(n)} - \alpha_i^{\setminus 0} + 1}}{\sum_{k'} \delta_{i,k'} \omega_{k'}^{(n)} \Gamma(\gamma_{k'}^{(n)})^{-1} (\kappa_{k'}^{(n)})^{\gamma_{k'}^{(n)}} \Gamma(\gamma_{k'}^{(n)} + \alpha_i^{\setminus 0} - 1)(\kappa_{k'}^{(n)} + \beta_i^{\setminus 0})^{-\gamma_{k'}^{(n)} - \alpha_i^{\setminus 0} + 1}} \\
\mathbb{E}_{g_n}[z_{i,k} \log t_i] &= \mathbb{E}_{g_n}[z_{i,k}] \left(\psi(\gamma_k^{(n)} + \alpha_i^{\setminus 0} - 1) - \log(\kappa_k^{(n)} + \beta_i^{\setminus 0}) \right) \\
\mathbb{E}_{g_n}[z_{i,k} t_i] &= \mathbb{E}_{g_n}[z_{i,k}] \frac{\gamma_k^{(n)} + \alpha_i^{\setminus 0} - 1}{\kappa_k^{(n)} + \beta_i^{\setminus 0}}
\end{aligned}
$$

and we've used the canonical parameterization $\theta_i^{\setminus 0} = (\alpha_i^{\setminus 0} - 1, -\beta_i^{\setminus 0})$. The maximization step $\boldsymbol{\eta}^{(n+1)} = \arg\max_{\boldsymbol{\eta}'} Q(\boldsymbol{\eta}' \parallel \boldsymbol{\eta}^{(n)})$ gives the updates

$$
\begin{aligned}
\omega_k^{(n+1)} &\propto \sum_{i \in \mathcal{N}} \mathbb{E}_{g_n}[z_{i,k}] \\
\gamma_k^{(n+1)}, \kappa_k^{(n+1)} &= \underset{\gamma', \kappa' > 0}{\arg\max} \sum_{i \in \mathcal{N}} (\gamma' - 1) \mathbb{E}_{g_n}[z_{i,k} \log t_i] - \kappa' \mathbb{E}_{g_n}[z_{i,k} t_i] - (\log \Gamma(\gamma') - \gamma' \log \kappa') \mathbb{E}_{g_n}[z_{i,k}].
\end{aligned}
$$

Note that the second line is equivalent to finding maximum likelihood estimates of gamma parameters from sufficient statistics Upon convergence, integrating over the optimized surrogate yields the approximate posterior moments for the ages of individual nodes, $\mathbb{E}_{q^{\setminus 0} p_0}[t_i] = \sum_k \mathbb{E}_{\hat{g}}[z_{i,k} t_i]$ and $\mathbb{E}_{q^{\setminus 0} p_0}[\log t_i] = \sum_k \mathbb{E}_{\hat{g}}[z_{i,k} \log t_i]$, that are used to calculate updated variational parameters θ_i' and prior messages $\theta_{i,0}' = \theta_i' - \theta_i^{\setminus 0}$.

7 Timescale calibration algorithm

Here we describe the time-rescaling algorithm used by tsdate. First we describe a simple approach that matches mutation counts and edge area over time, and then an extension that is robust to spurious polytomies in the genealogies.

Under the mutational clock, the expected total number of mutations that fall on an ARG in some time window $[a, b)$ is equal to the mutation rate μ multiplied by the total area of all edges contained in that window. Suppose an edge e between parent p_e and child c_e has span s_e and y_e mutations; the area of this edge that intersects $[a, b)$ is $\widehat{y}_{[a,b)}(e) = s_e \min(0, \min(b, t_{p_e}) - \max(a, t_{c_e}))$. The corresponding observed number of mutations on edge e is obtained by integrating over mutations' temporal positions on their edge: $\bar{y}_{[a,b)}(e) = y_e \min(0, \min(b, t_{p_e}) - \max(a, t_{c_e}))/(t_{p_e} - t_{c_e})$, The total expected and observed counts are obtained by summing over all edges: $\widehat{y}_{[a,b)} = \sum_{e \in \mathcal{E}} \widehat{y}_{[a,b)}(e)$ and $\bar{y}_{[a,b)} = \sum_{e \in \mathcal{E}} \bar{y}_{[a,b)}(e)$. Then, the interval length is rescaled so that expected and observed mutational counts match, mapping a and b to a' and b' where $b' - a' =$

bioRxiv preprint doi: https://doi.org/10.64898/2026.01.07.698223; this version posted January 12, 2026. The copyright holder for this preprint (which was not certified by peer review) is the author/funder, who has granted bioRxiv a license to display the preprint in perpetuity. It is made available under aCC-BY 4.0 International license.

$(b-a)\bar{y}_{[a,b)}/\widehat{y}_{[a,b)}$. The endpoints of all intervals are adjusted given the new lengths and the constraint that the first interval starts at zero, so that the age of a given node k located in $[a,b)$ is updated using the rescaled length and lower bound, to $t'_k = a' + (t_k - a)(a' - b')/(a - b)$.

There are two problems with applying this core idea. First, tsinfer encodes topological ambiguity in the ARG by polytomies. That is, a given node may have multiple children in a given inferred marginal tree when underlying true genealogy is binary. A consequence of these artefactual polytomies is, roughly, that the mutational area is overcounted, and rescaling based on observed counts of mutations will bias dates downwards. While the impact of polytomies on total branch area is minimal when the ARG contains relatively few samples, the bias can become extreme for large datasets when polytomies may contain hundreds or thousands of nodes.

To see the issue, consider a single tree in which a branch from parent i to child j has no mutations. In the inferred tree, i and j will be merged; but what should the "inferred" time of the resulting node be? There is no *a priori* best answer, but an argument can be made that the parent time t_i is a better choice: suppose that samples a and b have node j as their MRCA, and sample c has node i as its MRCA with a and b. Then if we were to place the merged node "ij" more recently than t_i, this would imply that a, b, and c share a common ancestor more recently than they actually do. Furthermore, the time t_{ij} of the merged node provides the upper bound on the time of any events occurring on the edge between i and j; so if t_{ij} is less than the true time t_i, we will incorrectly constrain the times of those events. (These "events" might be a stand-in for how information in this tree affects dating of nodes and mutations in adjacent trees.) On the other hand, setting the merged node's time t_{ij} to t_i results in an overestimate of the TMRCA of a and b (unless perhaps we interpret it as the "most recent inferrable ancestor"), but provides an accurate upper bound (given available information) on the times of those events.

The solution is to choose a statistic for the rescaling numerator and denominator that is not impacted by artefactual polytomies. Consider sampling a path from a randomly selected sample to the root in a given marginal tree. The number of mutations and length of the path is unaffected by any missing intermediate nodes (e.g., nodes which have been collapsed into their parent). Taking the average over all possible paths and summing across marginal trees leads to similar statistics as $\widehat{y}$ and $\bar{y}$, but weighted by the number of descendant samples. For a tree $\mathbb{T}$ let $s_{\mathbb{T}}$ denote the span of the tree, $d_{\mathbb{T}}(e)$ the number of samples descending from edge e in tree $\mathbb{T}$, and $y_{\mathbb{T}}(e)$ the number of mutations on edge e in the tree. Then, define

$$\widehat{Y}_{[a,b)}(e) = \mu \times \min(0, \min(b, t_{p_e}) - \max(a, t_{c_e})) \times \sum_{\mathbb{T}:e\in\mathbb{T}} s_{\mathbb{T}} d_{\mathbb{T}}(e)$$
$$\bar{Y}_{[a,b)}(e) = \frac{\min(0, \min(b, t_{p_e}) - \max(a, t_{c_e}))}{(t_{p_e} - t_{c_e})} \times \sum_{\mathbb{T}:e\in\mathbb{T}} y_{\mathbb{T}}(i,j) d_{\mathbb{T}}(e),$$

where the summations are over marginal trees that contain the edge e. As above, define $\widehat{Y}_{[a,b)}$ and $\bar{Y}_{[a,b)}$ as the sum of these over all edges, which may be computed efficiently in $\mathcal{O}(N + T\log N)$ time using the incremental strategy of,[47] where N is the number of samples in the ARG and T is the number of marginal trees. Subsequently, node ages are rescaled as described above for segregating sites but with $Y_{[a,b)}/Y_{[a,b)}$ as the rescaling factor.

The second challenge we need to solve is that rather than rescaling point estimates of node age we need to rescale variational posteriors, which implies integrating a distribution over a piecewise constant function to calculate sufficient statistics for moment matching. This is costly for large ARGs.

A solution is to rescale posteriors by matching quantiles rather than moments. We will choose

bioRxiv preprint doi: https://doi.org/10.64898/2026.01.07.698223; this version posted January 12, 2026. The copyright holder for this preprint (which was not certified by peer review) is the author/funder, who has granted bioRxiv a license to display the preprint in perpetuity. It is made available under aCC-BY 4.0 International license.

new parameters for each node i to match two things: the median and the ratio between the third and first quantiles. Denote the desired piecewise linear map to the transformed timescale as $t' = F(t)$. The value $F(t)$ can be computed in $\mathcal{O}(\log K)$ time given precomputed rescaling factors for K intervals, by using a binary search to find the interval bracketing t. Let $Q^{-1}(p|\alpha, \beta)$ (for $p \in [0, 1]$) denote the inverse CDF for the Gamma distribution with shape α and scale β. Recall that the Gamma distribution is a scale family with the property $Q^{-1}(p|\alpha, \beta) = \beta^{-1}Q^{-1}(p|\alpha, 1)$. Fix two quantiles $p_1 < p_2$ and define the function

$$f(\alpha) = \frac{Q^{-1}(p_2|\alpha, \beta)}{Q^{-1}(p_1|\alpha, \beta)} = \frac{Q^{-1}(p_2|\alpha, 1)}{Q^{-1}(p_1|\alpha, 1)},$$

which is monotonically decreasing in $\alpha > 0$, and therefore has an inverse that may be found numerically with a small number of iterations of Newton's method. Now suppose the variational posterior of node i is Gamma(α_i, β_i); we wish to find α'_i and β'_i such that $Q^{-1}(0.5, \alpha'_i, \beta'_i) = F(Q^{-1}(0.5, \alpha_i, \beta_i))$ and (with the arbitrary choice $p_1 = 0.25$ and $p_2 = 0.75$),

$$\frac{Q^{-1}(p_2, \alpha'_i, \beta'_i)}{Q^{-1}(p_1, \alpha'_i, \beta'_i)} = \frac{F(Q^{-1}(p_2, \alpha_i, \beta_i))}{F(Q^{-1}(p_1, \alpha_i, \beta_i))}.$$

To do this, we first solve $f(\alpha'_i) = F(Q^{-1}(p_2|\alpha_i, \beta_i))/F(Q^{-1}(p_1|\alpha_i, \beta_i))$ for α'_i, and then set $\beta'_i = Q^{-1}(0.5|\alpha'_i, 1)/F(Q^{-1}(0.5|\alpha_i, \beta_i))$.

8 Genomic properties of regions used for inference

We compared the genic content and recombination rate of regions selected for ARG inference of the GEL dataset (Methods §14) to those of randomly sampled autosomal regions of identical sizes, and used genome-wide allele age estimates to assess whether these genomic properties were associated with differences in allele age. All analyses were restricted to autosomes (chr1–22).

Inference regions were defined as the fixed set of autosomal segments used for ARG construction (listed in Tab S2). For all comparisons, region lengths were preserved exactly, and summary statistics were aggregated across all inference regions.

Protein-coding gene annotations were obtained from Ensembl (GRCh38; `https://ftp.ensembl.org/pub/release-115/gtf/homo_sapiens`). Gene intervals annotated as protein-coding were restricted to autosomes and reduced to a non-overlapping union. For each region set, the proportion of bases overlapping protein-coding genes was calculated as the total number of genic bases divided by the total genomic span of the regions. For inference regions, genic content was calculated per region and aggregated across all regions.

Recombination rates were estimated using fine-scale genetic maps from HapMap II lifted to GRCh38 coordinates. For each region, genetic map positions at the start and end coordinates were obtained by linear interpolation, and genetic distance was calculated as the difference between these values. Recombination rate was expressed as centimorgans per megabase (cM/Mb). Aggregate recombination rates were computed by dividing the total genetic distance by the total physical length across all regions.

Mean allele ages were obtained from genome-wide Relate[37] age estimates inferred using the 1,000 Genomes Project Yoruba (YRI) population (`https://zenodo.org/records/3234689`). For each region, Relate age estimates overlapping that interval were averaged, and an overall mean allele age was computed as a length-weighted mean across all regions.

bioRxiv preprint doi: https://doi.org/10.64898/2026.01.07.698223; this version posted January 12, 2026. The copyright holder for this preprint (which was not certified by peer review) is the author/funder, who has granted bioRxiv a license to display the preprint in perpetuity. It is made available under aCC-BY 4.0 International license.

To generate null distributions, 100 random region sets were sampled sampled from the callable autosomal genome, defined as autosomes excluding centromeric regions and annotated assembly gaps. Centromeres were identified using UCSC cytoband annotations (acen and gvar bands; `https://hgdownload.soe.ucsc.edu/goldenPath/hg38/database/cytoBand.txt.gz`), and assembly gaps were excluded using UCSC gap annotations for GRCh38 (`https://hgdownload.soe.ucsc.edu/goldenPath/hg38/database/gap.txt.gz`). Each replicate consisted of one region per inference segment, matched exactly by length. Regions were sampled uniformly across all valid genomic placements within the callable genome. To avoid positional bias, callable intervals were weighted by the number of valid start positions they contained for a given region length. For each replicate, genic content, recombination rate, and mean allele ages were recomputed using the same procedures applied to the observed inference regions.

The inference regions showed clear deviations from random expectations in terms of genic content and recombination rate (Supp Fig S13.a,b) — consistent with broad-scale chromosomal variation in gene density and recombination rate, given that the inference regions are concentrated on chromosomes 17–22, which include some of the most gene-dense human chromosomes (notably chromosomes 17[132] and 19[133]) and are characterised by elevated recombination rates per unit physical length owing to the inverse relationship between chromosome size and recombination rate.[134] In contrast, the mean allele age estimated from Relate YRI data for the inference regions lay well within the distribution obtained from random, length-matched regions (Supp Fig S13.c), with no strong deviation from the null expectation. This indicates that, despite being enriched for genic sequence and higher recombination the inference regions are not detectably biased with respect to ARG-derived genome-wide allele age estimates.

9 Age vs frequency as a predictor of selection

Using data simulated from a model of human evolution under purifying selection (Methods §12), we assessed the ability of mutation age versus allele frequency to predict the known selection coefficients, s, associated with each mutation. We considered simulations under balanced versus unbalanced sampling, which generated $n = 1{,}419{,}535$ vs $n = 1{,}000{,}412$ non-recurrent mutations respectively. We also repeated the analysis restricting these two datasets to ultra-rare variants only (DAF < 0.001; $n = 1{,}110{,}527$ vs $n = 761{,}867$).

Treating the selection coefficient s as a response variable, we fitted an additive linear model in R[135] using the logarithm of age plus the logarithm of frequency as predictors, and an additional model which also included the mutation's population of origin as a separate additive factor. The simulated DFE generates a highly skewed distribution of selection coefficients, with a majority of mutations being neutral ($s = 0$), resulting in residuals which do not conform to a simple statistical distribution. We therefore did not calculate p-values, but simply measure the variance contribution (pseudo R^2) of the age versus frequency term by dropping each independently from the full model. Pseudo R^2 values were calculated as the proportional reduction in deviance, $1 - D_{\text{reduced}}/D_{\text{full}}$, where D_{reduced} is the deviance of the model with the dropped term. We report the R^2 contributions of the age and frequency terms, and also calculate the ratio of the two values, to produce a measure of the relative predictive ability of age versus frequency in each model. Finally, we fitted models in which mutation age was taken either as the true (t_{true}) or the tsinfer+tsdate inferred (t_{tsdate}) midpoint time.

The results in Tab. S8 show that under a balanced sampling scheme, age and frequency are roughly equivalent in predictive power, to within a factor of 2. If true (midpoint) age is known, then over the whole dataset, age provides about twice as much information as frequency when

bioRxiv preprint doi: https://doi.org/10.64898/2026.01.07.698223; this version posted January 12, 2026. The copyright holder for this preprint (which was not certified by peer review) is the author/funder, who has granted bioRxiv a license to display the preprint in perpetuity. It is made available under aCC-BY 4.0 International license.

predicting the selection coefficient, even if population-of-origin is taken into account. However, for ultra-rare variants, the relative power is reduced, and using inferred age results in frequency having slightly more predictive power. Nevertheless, over all the results, even the lowest R^2 for age is 6.539e-6, which is comparable in magnitude to the highest R^2 for frequency (1.684e-5).

Under the unbalanced sampling regime, allele age consistently explains orders of magnitude more variation in the selection coefficient than allele frequency. This is true both using inferred age or the true age as a predictor: in the latter case, age accounts for the entire prediction: the additional contribution of frequency is negligible. Accounting for population-of-origin somewhat reduces this difference, nevertheless in our models age always remains a better predictor of (negative) selection when sampling is unbalanced, even for ultra-rare alleles. We also note that the higher predictive power of age over frequency is maintained when changing the model to use the raw (unlogged) frequency values as a predictor (data not shown).

This simulation and analysis illustrates the potential predictive power of allele age; further work is required, however, to understand more fully these properties over a range of evolutionary scenarios. In practice, of course, both frequency (in the sample) and (estimated) age are available.

bioRxiv preprint doi: https://doi.org/10.64898/2026.01.07.698223; this version posted January 12, 2026. The copyright holder for this preprint (which was not certified by peer review) is the author/funder, who has granted bioRxiv a license to display the preprint in perpetuity. It is made available under aCC-BY 4.0 International license.

Supplementary Tables and Figures

In this section we include all supplementary tables and figures.

Table S1: Accuracy of the new Variational Gamma (VG) algorithm compared to the original Inside-Outside (IO) algorithm in tsdate. Accuracy was assessed by comparing true (simulated) and inferred (`tsinfer` and `tsdate`) ARGs across four human `stdpopsim` models, over a 40 Mb section of chromosome 17 with 15000 diploids sampled evenly across populations. Root mean square error (RMSE) and bias are calculated for $\log_{10}$ mutation ages (estimated as edge midpoints), for all mutations and for low-frequency mutations segregating in $< 0.1\%$ of sample haplotypes (shown in parentheses). Statistics are shown separated by frequency in Fig. S1.

	VG		IO	
`stdpopsim` model	Bias	RMSE	Bias	RMSE
`AmericanAdmixture_4B18`	−0.01 (−0.01)	0.27 (0.27)	−0.32 (−0.38)	0.48 (0.53)
`OutOfAfricaArchaicAdmixture_5R19`	−0.03 (−0.04)	0.32 (0.33)	−0.38 (−0.46)	0.58 (0.65)
`OutOfAfrica_3G09`	−0.01 (−0.01)	0.27 (0.27)	−0.30 (−0.36)	0.47 (0.51)
`Zigzag_1S14`	−0.00 (0.00)	0.31 (0.31)	−0.16 (−0.21)	0.40 (0.44)

bioRxiv preprint doi: https://doi.org/10.64898/2026.01.07.698223; this version posted January 12, 2026. The copyright holder for this preprint (which was not certified by peer review) is the author/funder, who has granted bioRxiv a license to display the preprint in perpetuity. It is made available under aCC-BY 4.0 International license.

Table S2: Inference regions. Details of the 15 regions used for inference, including coordinates, genic proportion, and average recombination rate (cM/Mb).

region	start	end	length	gene content	recomb rate
16p1	16,689	14,637,672	14,620,983	0.712	2.236
16p2	22,661,538	28,286,126	5,624,588	0.531	2.237
17p1	281,784	18,276,569	17,994,785	0.661	2.387
17q1	26,718,757	36,149,680	9,430,923	0.670	1.142
17q2	36,458,468	37,824,094	1,365,626	0.633	2.059
17q3	38,221,639	45,432,621	7,210,982	0.643	0.833
17q4	45,586,151	83,087,382	37,501,231	0.594	1.636
18p	148,918	15,357,916	15,208,998	0.479	2.626
19p	75,006	24,174,775	24,099,769	0.714	2.044
20p	227,205	26,274,525	26,047,320	0.487	1.951
20q1	31,254,980	64,237,737	32,982,757	0.524	1.733
21q1	34,495,543	42,960,010	8,464,467	0.726	2.275
21q2	44,253,397	46,680,226	2,426,829	0.702	1.713
22q1	16,439,646	18,110,684	1,671,038	0.560	3.230
22q2	18,897,685	21,045,540	2,147,855	0.715	2.980
Total	N/A	N/A	206,798,151	0.600	1.942

Table S3: CPU resources for ARG inference. For each region listed in Tab. S2 we show the CPU time consumed by different phases of inference. Note that this is the cumulative user time across all threads and processes and not the elapsed wall-clock time.

region	gen_ancestors	match_ancestors	match_samples	tsdate	total
16p1	8.28h	6.37d	1.54y	14.03m	1.56y
16p2	1.16h	2.65d	88.04d	6.37m	90.74d
17p1	17.96h	14.13h	2.62y	19.45m	2.63y
17q1	2.23h	5.29d	176.03d	7.58m	181.42d
17q2	13.89m	3.44h	15.54d	2.69m	15.70d
17q3	1.54h	3.92d	112.25d	5.79m	116.24d
17q4	1.06d	6.23d	1.89y	41.56m	1.91y
18p	5.92h	9.51d	1.49y	16.19m	1.52y
19p	12.92h	6.63d	2.15y	14.85m	2.17y
20p	1.29d	11.72h	3.29y	22.99m	3.29y
20q1	1.12d	1.89d	1.34y	39.86m	1.35y
21q1	2.65h	5.72d	196.69d	9.85m	202.53d
21q2	30.34m	18.85h	36.83d	1.42m	37.64d
22q1	8.26m	5.71h	14.66d	3.18m	14.90d
22q2	13.16m	6.70h	36.47d	3.53m	36.76d
Total	5.71d	50.74d	16.18y	3.49h	16.33y

bioRxiv preprint doi: https://doi.org/10.64898/2026.01.07.698223; this version posted January 12, 2026. The copyright holder for this preprint (which was not certified by peer review) is the author/funder, who has granted bioRxiv a license to display the preprint in perpetuity. It is made available under aCC-BY 4.0 International license.

Table S4: Tree sequence statistics by region in the GEL dataset. For each region listed in Tab. S2 we show the number of variant sites used in inference (sites); the number of trees, nodes, edges, and mutations in the final inferred tree sequence; and the size of tszip file.

region	length (bp)	sites	trees	nodes	edges	muts	file size
16p1	14,620,983	2,207,614	708,992	1,793,817	14,582,277	2,207,614	247.3MB
16p2	5,624,588	648,035	202,069	531,263	3,544,965	648,035	64.5MB
17p1	17,994,785	2,054,172	706,131	1,683,050	13,588,316	2,054,172	244.5MB
17q1	9,430,923	963,103	303,130	738,625	4,403,891	963,103	88.3MB
17q2	1,365,626	148,058	41,648	195,915	925,266	148,058	14.2MB
17q3	7,210,982	767,500	234,079	589,059	3,379,624	767,500	65.6MB
17q4	37,501,231	4,054,901	1,252,598	2,949,271	22,785,855	4,054,901	432.0MB
18p	15,208,998	1,600,531	490,490	1,271,875	11,525,869	1,600,531	189.4MB
19p	24,099,769	2,396,917	797,814	1,981,628	17,134,593	2,396,917	284.5MB
20p	26,047,320	2,934,752	858,060	2,093,350	16,711,709	2,934,752	304.1MB
20q1	32,982,757	3,783,588	1,153,190	2,752,320	21,697,573	3,783,588	403.6MB
21q1	8,464,467	988,743	326,829	827,212	6,195,030	988,743	110.7MB
21q2	2,426,829	304,942	109,770	342,957	2,007,819	304,942	34.0MB
22q1	1,671,038	147,455	53,852	220,890	1,352,677	147,455	19.5MB
22q2	2,147,855	223,988	75,972	261,365	1,448,721	223,988	23.4MB
Total	206,798,151	23,224,299	7,314,624	16,806,547	141,284,185	23,224,299	2.5GB

Table S5: Allele age comparisons by clinical class. G denotes the geometric mean allele age (in generations). For doubletons, results are shown for variants estimated to be likely recurrent and likely non-recurrent using a genealogical consistency filter (Methods §23). Statistical tests evaluate whether pathogenic or likely pathogenic (P/LP) variants are younger than the comparison class using permutation tests.

Count	Class	n	G (age)	p-value
**Singletons**				
	P/LP	749	44	–
	B/LB	380	60	< 0.0001
	VUS	6,335	54	< 0.0001
	P/LP (GEL)	52	33	0.0052
	P/LP (ClinVar)	770	46	–
**Doubletons (likely recurrent)**				
	P/LP	108	150	–
	B/LB	117	145	0.83
	VUS	1,461	136	0.393
**Doubletons (likely non-recurrent)**				
	P/LP	107	55	–
	B/LB	129	81	0.0012
	VUS	1,821	76	0.0066

bioRxiv preprint doi: https://doi.org/10.64898/2026.01.07.698223; this version posted January 12, 2026. The copyright holder for this preprint (which was not certified by peer review) is the author/funder, who has granted bioRxiv a license to display the preprint in perpetuity. It is made available under aCC-BY 4.0 International license.

Table S6: Example of estimated ancient doubletons (DAC=2) classified as variants of uncertain significance (VUS) in the GEL dataset. All 6 are classified as Missense variants. Estimated age is reported in generations before the present.

Chr	Pos	rsID	Gene	Gene MOI	Ggroup	Age	95% CI
21	44,525,821	rs139112987	TSPEAR	Recessive	AFR	35,340	6,148–89,540
22	20,090,255	rs777348779	DGCR8	Dominant	EAS	21,657	3,028–58,215
16	580,192	rs1361495767	PIGQ	Recessive	NFE	9,465	728–29,108
17	75,842,559	rs551855408	UNC13D	Recessive	AFR	5,596	527–16,503
19	5,110,736	rs1466512977	KMD4B	Dominant	NFE	5,369	333–17,197
17	77,488,314	rs762543093	SEPTIN9	Dominant	NFE	4,828	1,209–10,903

Table S7: Per-region tree sequence statistics for the 1000 Genomes Project dataset. The regions are a subset of those detailed in Tab. S2. See Tab. S4 for details on the columns shown.

region	length (bp)	sites	trees	nodes	edges	muts	file size
17p	17,994,697	596,776	265,521	528,258	3,482,633	596,776	218.5MB
17q1	9,163,785	263,697	105,366	203,601	1,171,136	263,697	82.0MB
17q2	1,337,198	40,576	15,856	37,064	198,920	40,576	14.0MB
17q3	7,230,866	211,258	80,931	163,430	933,648	211,258	65.6MB
17q4	37,497,391	1,162,183	465,421	937,537	5,941,940	1,162,183	389.8MB
20p	25,524,955	849,268	350,460	661,927	4,065,850	849,268	273.9MB
20q	34,481,295	1,087,525	441,943	847,444	5,102,012	1,087,525	347.0MB
Total	133,230,187	4,211,283	1,725,498	3,379,261	20,896,139	4,211,283	1.4GB

bioRxiv preprint doi: https://doi.org/10.64898/2026.01.07.698223; this version posted January 12, 2026. The copyright holder for this preprint (which was not certified by peer review) is the author/funder, who has granted bioRxiv a license to display the preprint in perpetuity. It is made available under aCC-BY 4.0 International license.

Table S8: Variance explained by mutation age versus allele frequency in predicting negative selection. Proportional reduction in deviance (pseudo R^2) when age or frequency terms are removed from linear models that predict the selection coefficient (s). Two simulated datasets were used, comprising 60,000 samples of a 40Mb region of human chromosome 17.

**Sampling**	**Model**	**Data**	**Age R^2**	**Freq R^2**	**Ratio**
Balanced	$s \sim \log(\text{freq}) + \log(t_{\text{true}})$	all	1.932e-5	8.718e-6	2.216
		ultra-rare	1.64e-5	1.192e-5	1.376
	$s \sim \log(\text{freq}) + \log(t_{\text{tsdate}})$	all	1.087e-5	1.315e-5	0.8267
		ultra-rare	7.97e-6	1.684e-5	0.4733
	$s \sim \log(\text{freq}) + \log(t_{\text{true}}) + \text{pop}$	all	1.796e-5	8.546e-6	2.102
		ultra-rare	1.464e-5	1.129e-5	1.297
	$s \sim \log(\text{freq}) + \log(t_{\text{tsdate}}) + \text{pop}$	all	9.728e-6	1.298e-5	0.7494
		ultra-rare	6.539e-6	1.656e-5	0.3949
Unbalanced	$s \sim \log(\text{freq}) + \log(t_{\text{true}})$	all	0.0001445	6.393e-9	22596
		ultra-rare	0.0001521	3.297e-8	4613
	$s \sim \log(\text{freq}) + \log(t_{\text{tsdate}})$	all	0.000107	4.142e-7	258.4
		ultra-rare	0.0001109	4.133e-7	268.3
	$s \sim \log(\text{freq}) + \log(t_{\text{true}}) + \text{pop}$	all	4.49e-5	6.416e-7	69.98
		ultra-rare	2.663e-5	1.847e-6	14.42
	$s \sim \log(\text{freq}) + \log(t_{\text{tsdate}}) + \text{pop}$	all	2.431e-5	3.772e-6	6.444
		ultra-rare	1.121e-5	7.2e-6	1.558

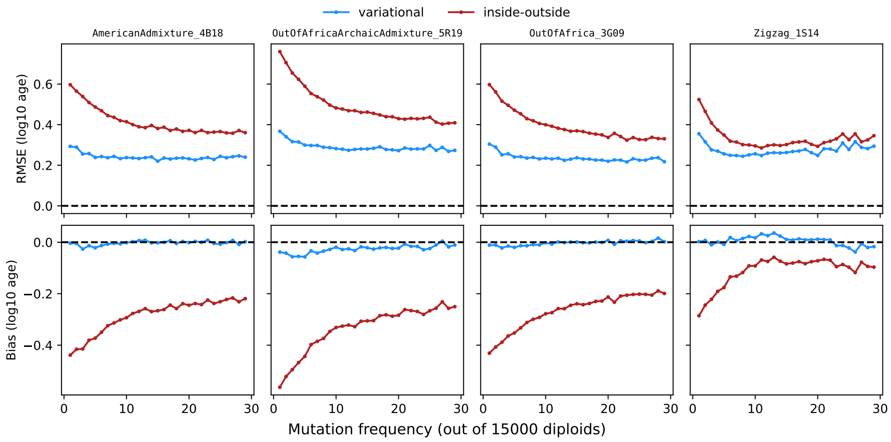

Figure S1: Accuracy of the algorithm described in this study ("variational") compared to the previous version in tsdate ("inside-outside"). Accuracy was assessed by comparing true (simulated) and inferred (tsinfer and tsdate) ARGs across four human stdpopsim models, over a 40 Mb section of chromosome 17 with 15,000 diploids sampled evenly across populations. Root mean square error (RMSE) and bias are calculated for $\log_{10}$ mutation ages (estimated as edge midpoints), separately for mutations at various frequencies in the sample.

bioRxiv preprint doi: https://doi.org/10.64898/2026.01.07.698223; this version posted January 12, 2026. The copyright holder for this preprint (which was not certified by peer review) is the author/funder, who has granted bioRxiv a license to display the preprint in perpetuity. It is made available under aCC-BY 4.0 International license.

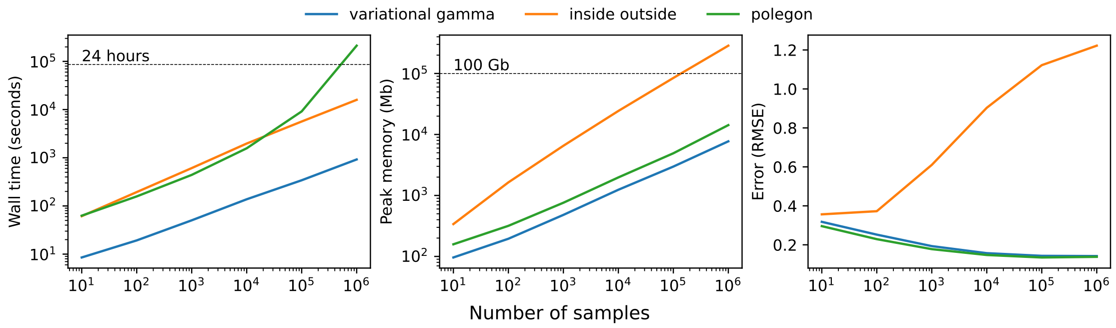

Figure S2: Benchmarks of the `variational_gamma` algorithm, alongside the original `inside_outside` algorithm in tsdate and POLEGON. All three methods were applied to a realistic, pedigree-based simulation of human chromosome 22, randomly subsampled to various numbers of samples From left to right are (median of three replicates) wall time in seconds, peak memory usage in Mb, and root mean squared error of estimated $\log_{10}$ node ages.

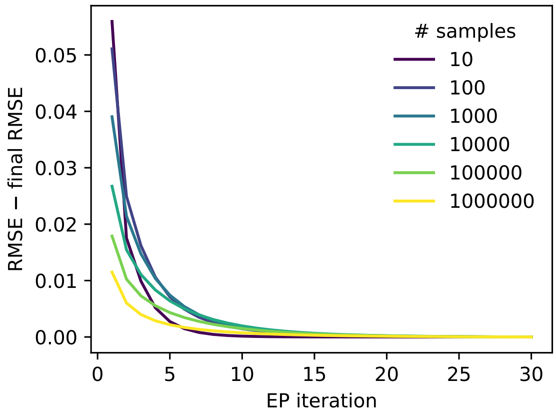

Figure S3: Convergence of the tsdate `variational_gamma` algorithm with iteration of the expectation propagation scheme. Convergence was measured as root mean square error (RMSE) in node ages at a given iteration, minus the RMSE at the final iteration, averaged over three replicate ARGs per sample size. Each replicate was generated by randomly subsampling a large, pedigree-based simulation of human chromosome 22.

bioRxiv preprint doi: https://doi.org/10.64898/2026.01.07.698223; this version posted January 12, 2026. The copyright holder for this preprint (which was not certified by peer review) is the author/funder, who has granted bioRxiv a license to display the preprint in perpetuity. It is made available under aCC-BY 4.0 International license.

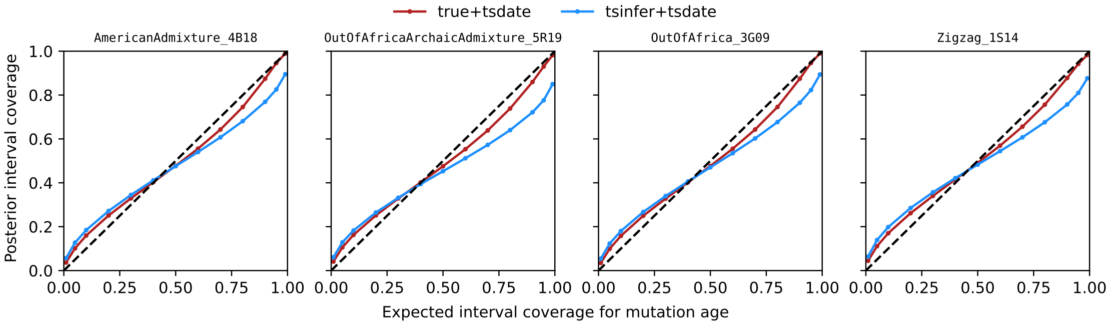

Figure S4: Calibration of the approximate posteriors for mutation ages produced by the tsdate `variational_gamma` algorithm. tsdate was applied to both true (simulated) and inferred (tsinfer) ARGs across four human stdpopsim models, over a 40 Mb section of chromosome 17 with 15,000 diploids sampled evenly across populations. Calibration can be quantified by comparing the proportion of true ages that fall within posterior intervals to what is expected given the interval widths, a perfectly calibrated method would fall on the $y = x$ line in these plots. The approximate posteriors from the true ARGs provide reasonable estimates of uncertainty, especially for the widest intervals, despite the assumption of a particular parametric form. In contrast, the approximate posteriors from the inferred ARGs underestimate uncertainty – for instance, a 99% interval is roughly equivalent to a 90% interval. This is expected, because the uncertainty reported by tsdate does not model the additional error from the tsinfer topology inference.

bioRxiv preprint doi: https://doi.org/10.64898/2026.01.07.698223; this version posted January 12, 2026. The copyright holder for this preprint (which was not certified by peer review) is the author/funder, who has granted bioRxiv a license to display the preprint in perpetuity. It is made available under aCC-BY 4.0 International license.

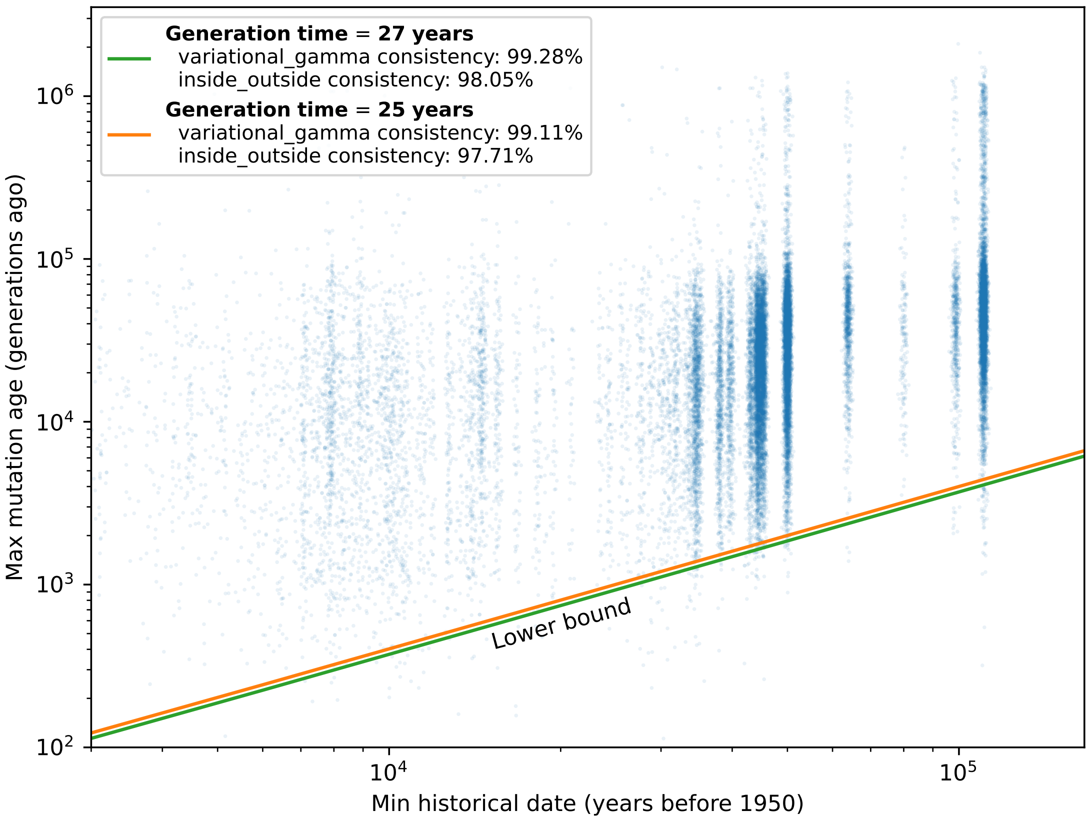

Figure S5: Allelic ages inferred by tsinfer+tsdate from the 1000 Genomes dataset (chromosome 20) compared to minimum variant ages from the Allen Ancient DNA Resource. See Methods §8 for details. Ancient DNA dates range from 0.1ka to 111ka.

bioRxiv preprint doi: https://doi.org/10.64898/2026.01.07.698223; this version posted January 12, 2026. The copyright holder for this preprint (which was not certified by peer review) is the author/funder, who has granted bioRxiv a license to display the preprint in perpetuity. It is made available under aCC-BY 4.0 International license.

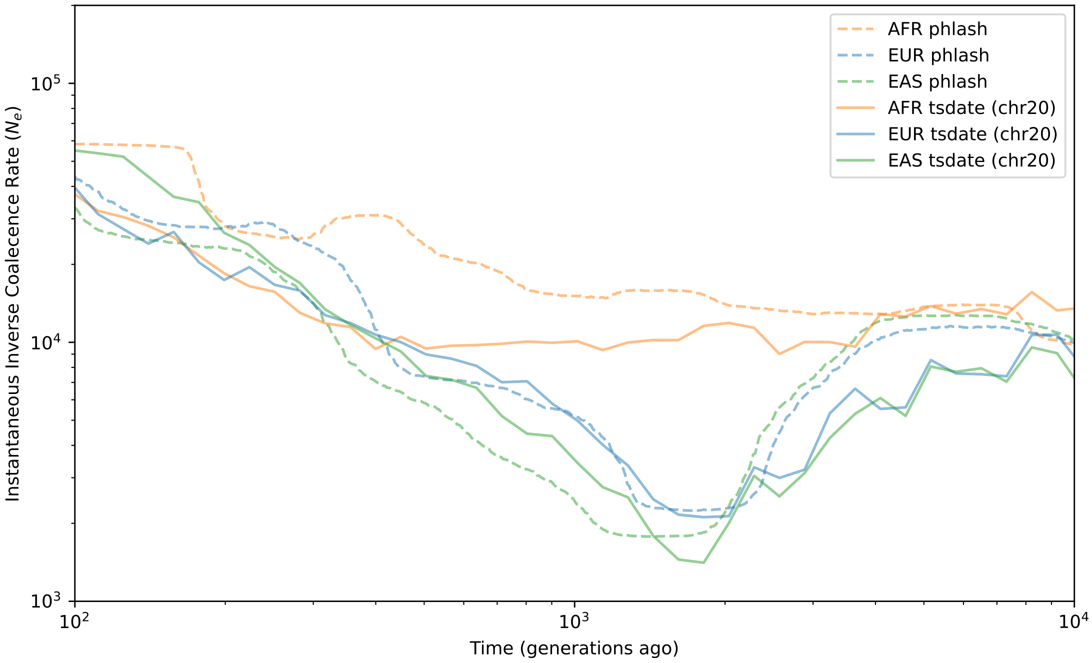

Figure S6: Inverse instantaneous coalescence rates (N_e) inferred by tsinfer+tsdate from the 1000 Genomes dataset (chromosome 20), focussing on the proposed time period for the Out of Africa bottleneck. Previously published estimates from PHLASH are also shown.[51] See Methods §9 for details. The plot spans the time period 2.7ka to 270ka (assuming a generation time of 27 years).

bioRxiv preprint doi: https://doi.org/10.64898/2026.01.07.698223; this version posted January 12, 2026. The copyright holder for this preprint (which was not certified by peer review) is the author/funder, who has granted bioRxiv a license to display the preprint in perpetuity. It is made available under aCC-BY 4.0 International license.

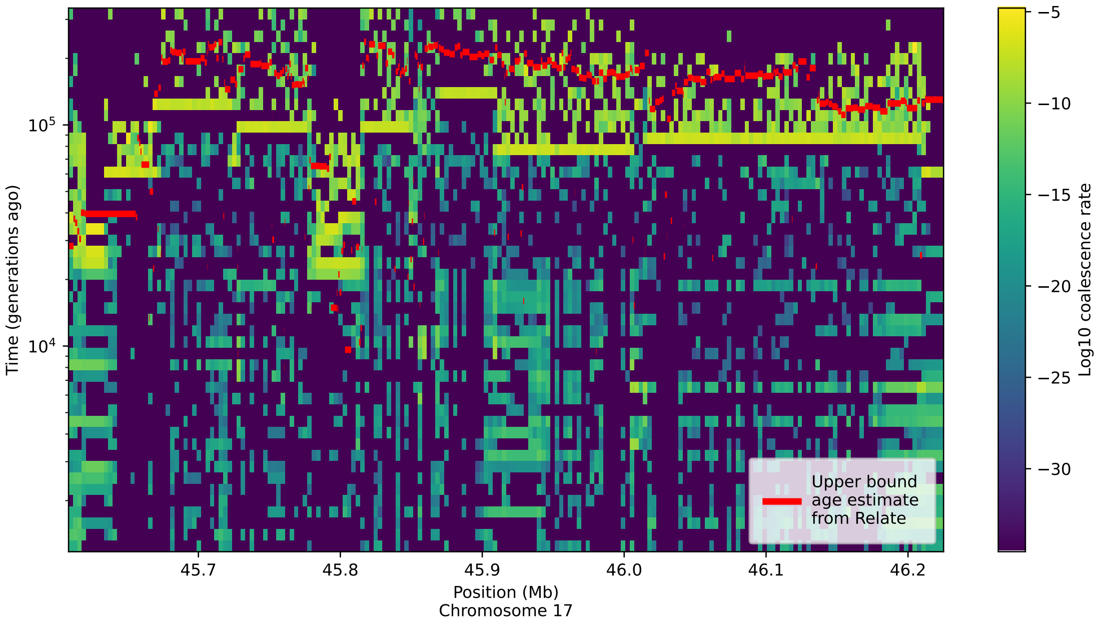

Figure S7: Dated cross coalescence rates inferred by tsinfer+tsdate between carriers and non-carriers of a known ancient inversion on chromosome 17 of the 1000 Genomes dataset. The upper bounds for the inversion from a published estimate which range from 160 ka to 6417 ka (assuming a generation time of 27 years), are shown in red. See Methods §10 for details.

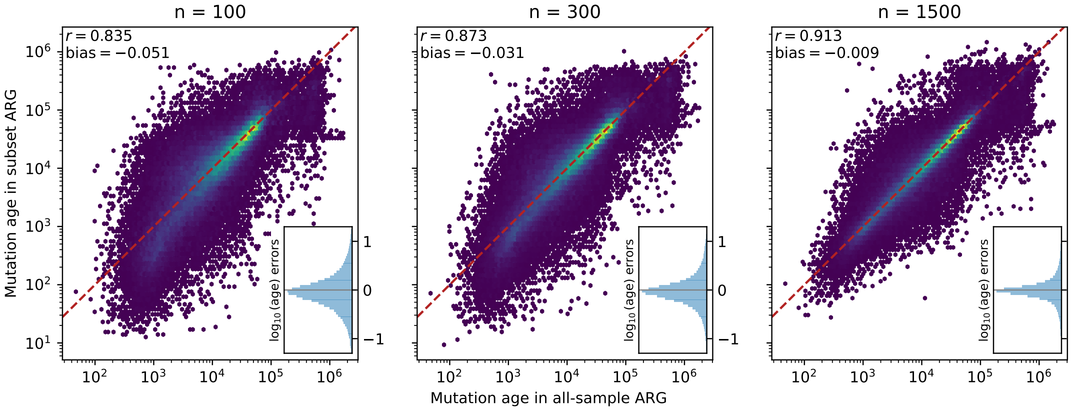

Figure S8: Dependence of mutation age estimates on sample size in the 1000 Genomes Project data. Mutation age estimates based on ARGs inferred from three subsets (100, 300 and 1500 samples) compared to estimates from the complete dataset. See Methods §7 for details.

bioRxiv preprint doi: https://doi.org/10.64898/2026.01.07.698223; this version posted January 12, 2026. The copyright holder for this preprint (which was not certified by peer review) is the author/funder, who has granted bioRxiv a license to display the preprint in perpetuity. It is made available under aCC-BY 4.0 International license.

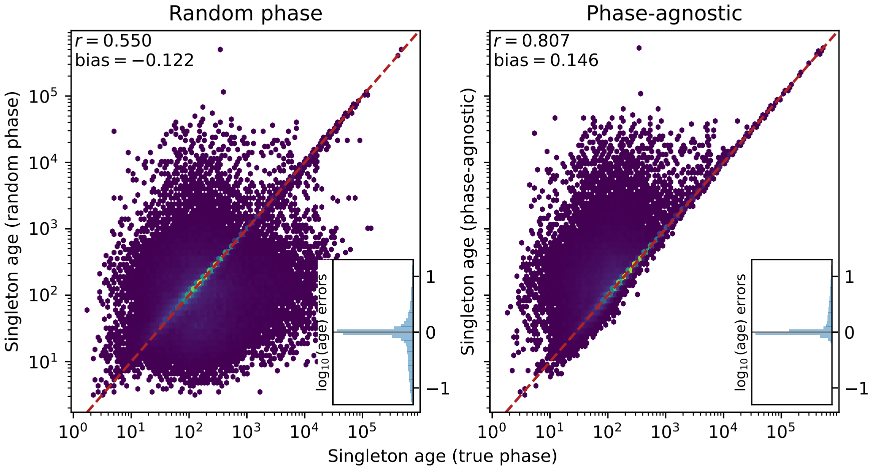

Figure S9: Validation of phase-agnostic singleton mutation dating using the 1000 Genomes Project data. We used 106,863 sites that are singletons in an ARG inferred from a subset of 1,500 individuals, but of higher frequency in the all-sample ARG. Age estimates of singletons obtained using their true phase are compared to those using a randomly assigned phase (left) and the phase-agnostic dating algorithm (right). See Methods §7 for details.

bioRxiv preprint doi: https://doi.org/10.64898/2026.01.07.698223; this version posted January 12, 2026. The copyright holder for this preprint (which was not certified by peer review) is the author/funder, who has granted bioRxiv a license to display the preprint in perpetuity. It is made available under aCC-BY 4.0 International license.

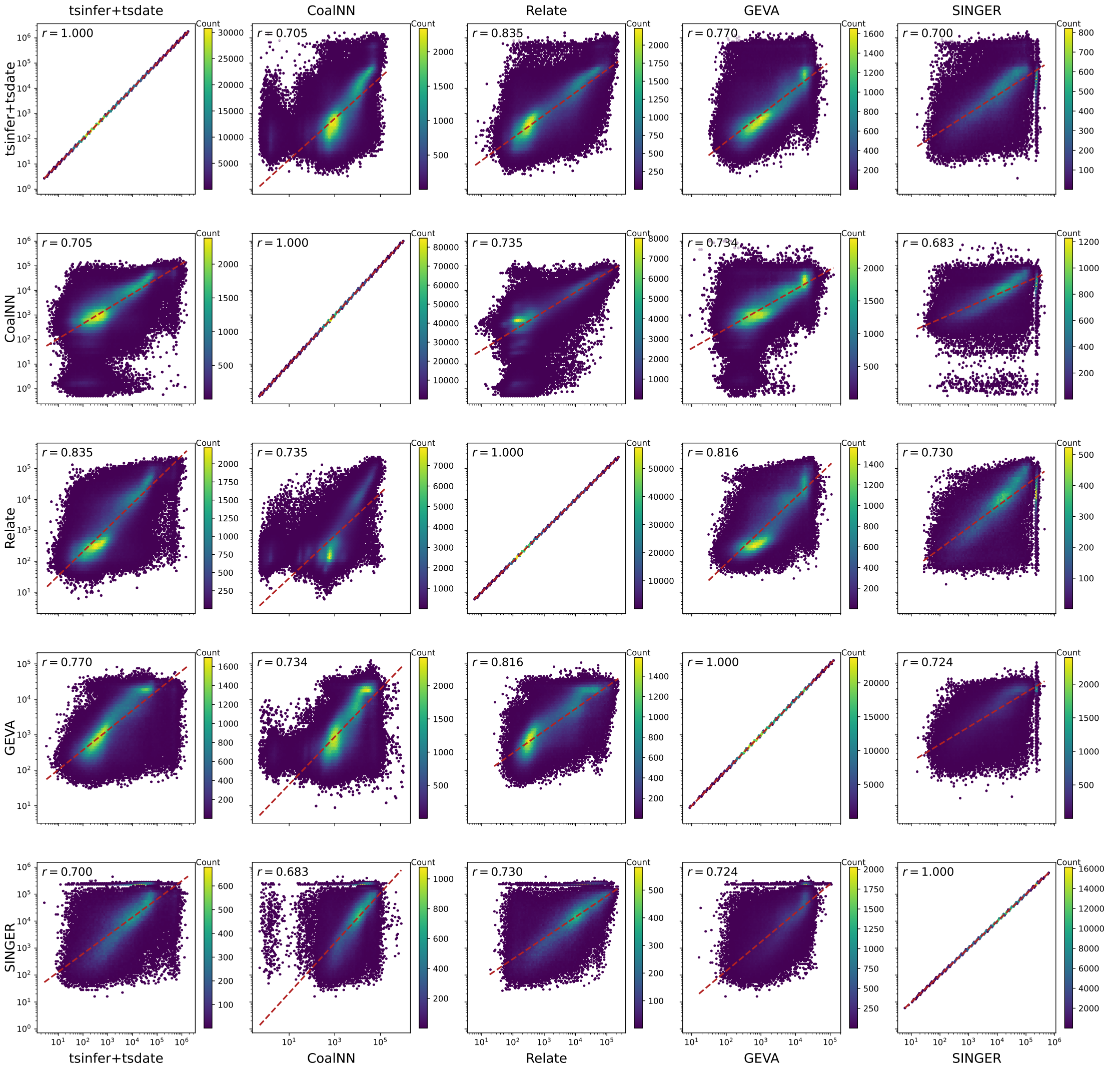

Figure S10: Pairwise comparison of allele age estimates obtained from five methods using chromosome 20 of the 1000 Genomes Project data. Results are restricted to a subset of 162,556 sites that are present in all datasets and have identical allele polarisation. See Methods §11 for details.

bioRxiv preprint doi: https://doi.org/10.64898/2026.01.07.698223; this version posted January 12, 2026. The copyright holder for this preprint (which was not certified by peer review) is the author/funder, who has granted bioRxiv a license to display the preprint in perpetuity. It is made available under aCC-BY 4.0 International license.

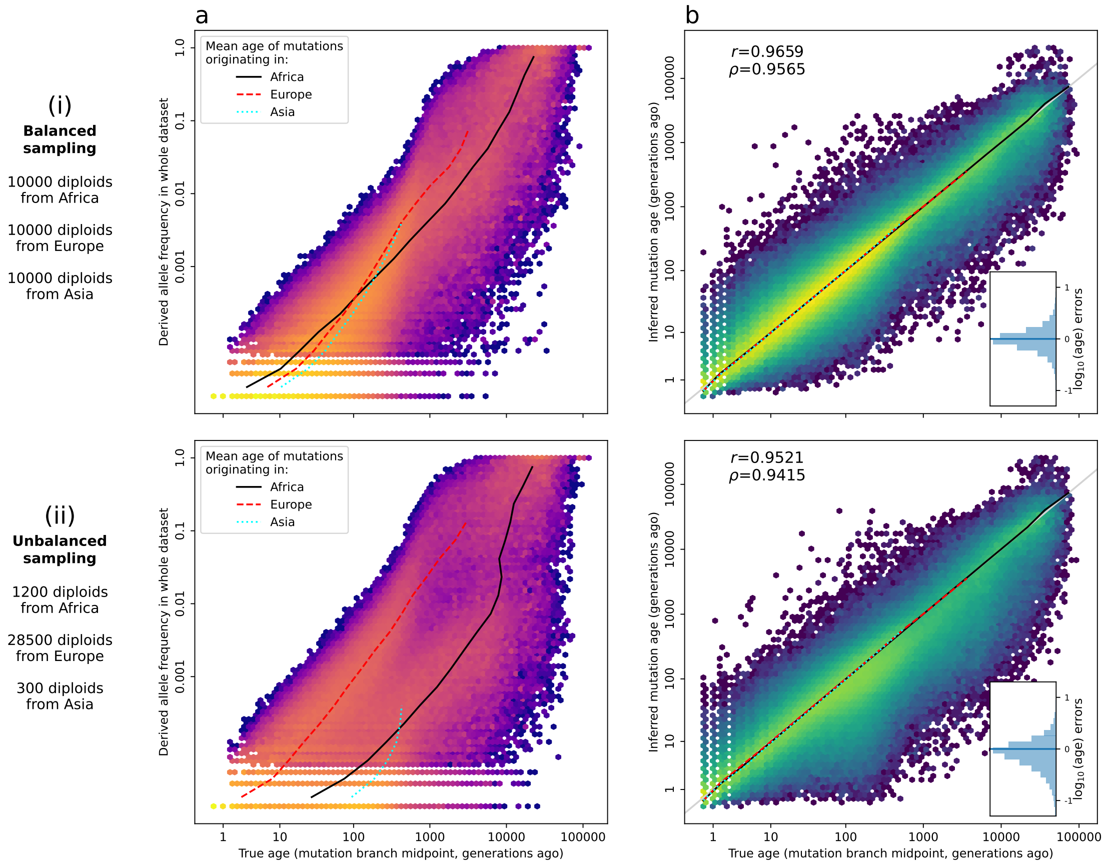

Figure S11: Robustness to unbalanced sampling. Distributions of mutation ages and frequencies in a 40 MB segment of human chromosome 17 from a 3-population Out-of-Africa simulation with negative selection under balanced (top row) and unbalanced (bottom row) sampling regimes. Data is restricted to the 98% of polymorphic sites with only one mutation. (left plots) Allele frequency against true age; solid/dotted/dashed lines lines indicate binned mean mutation ages for the 3 populations in 20 evenly-spaced log-frequency bins. (right plots) Inferred age against true age; lines as in (i), and residual inferred ages in inset histograms. Although the relationship between age and frequency breaks down with unbalanced sampling (compare bottom-left to top-left plot), age estimation is not affected (compare bottom-right and top-right plots).

bioRxiv preprint doi: https://doi.org/10.64898/2026.01.07.698223; this version posted January 12, 2026. The copyright holder for this preprint (which was not certified by peer review) is the author/funder, who has granted bioRxiv a license to display the preprint in perpetuity. It is made available under aCC-BY 4.0 International license.

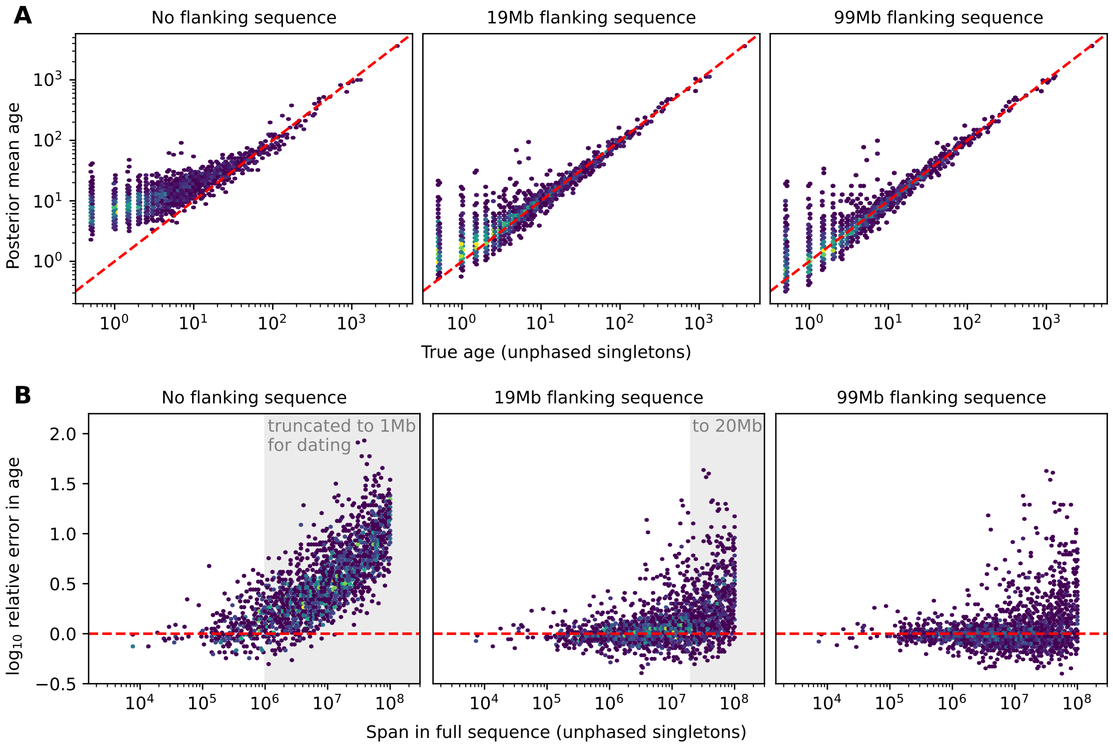

Figure S12: ARGs with short sequence lengths lead to bias in the estimated ages of recent mutations. (A) True versus estimated ages of unphased singleton mutations, from a 1Mb interval in the center of a 100Mb simulated tree sequence with 40k haploids. Each panel shows the same mutations, dated on subsegments of the tree sequence of progressively larger lengths (1Mb, 20Mb, and 100Mb). Substantial upwards bias in the youngest mutation ages is evident for shorter sequences. (B) The error in estimated ages versus the span of the edge. The singletons in the gray region are on edges that must have been truncated by removing the flanking regions prior to dating. Thus, truncating long, recent haplotypes leads to substantial dating error.

bioRxiv preprint doi: https://doi.org/10.64898/2026.01.07.698223; this version posted January 12, 2026. The copyright holder for this preprint (which was not certified by peer review) is the author/funder, who has granted bioRxiv a license to display the preprint in perpetuity. It is made available under aCC-BY 4.0 International license.

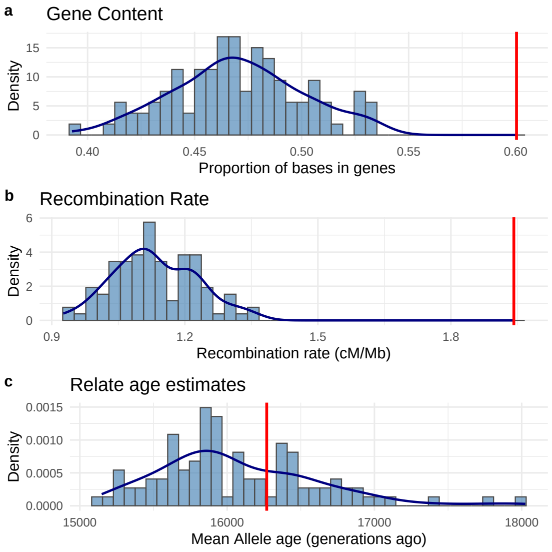

Figure S13: Comparison of genomic properties between the GEL ARG inference regions (red line; Table S2) and matched random autosomal regions (blue bars). The GEL estimate aggregates values across all 15 inference regions. Each blue bar corresponds to one of 100 independent replicates, each comprising 15 regions matched to the inference regions by size. Panels show (a) gene content (fraction of bases within ENSEMBL protein-coding genes), (b) average recombination rate (cM/Mb; HapMap II), and (c) mean allele age inferred from the Relate Yoruba (YRI) dataset.

bioRxiv preprint doi: https://doi.org/10.64898/2026.01.07.698223; this version posted January 12, 2026. The copyright holder for this preprint (which was not certified by peer review) is the author/funder, who has granted bioRxiv a license to display the preprint in perpetuity. It is made available under aCC-BY 4.0 International license.

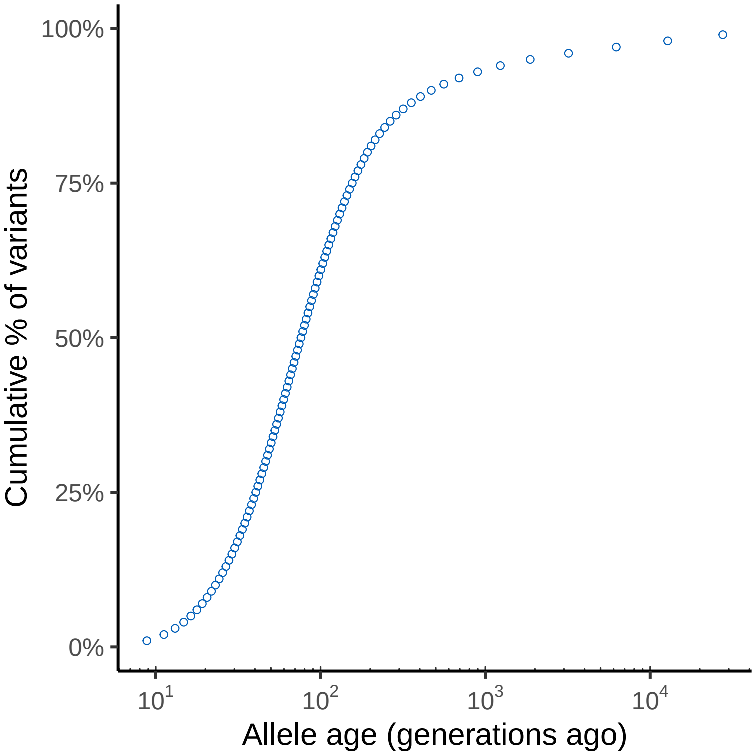

Figure S14: Cumulative distribution function of estimated variant ages in the GEL dataset.

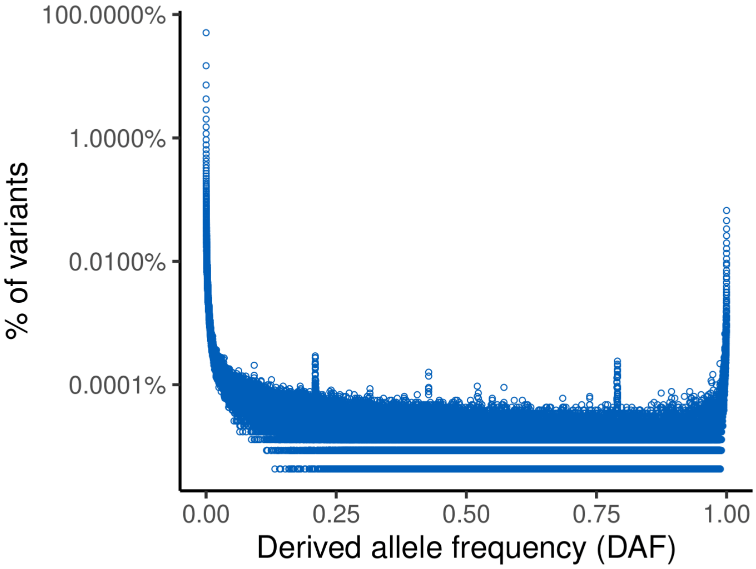

Figure S15: Derived allele frequency spectrum (AFS) in the GEL dataset.

bioRxiv preprint doi: https://doi.org/10.64898/2026.01.07.698223; this version posted January 12, 2026. The copyright holder for this preprint (which was not certified by peer review) is the author/funder, who has granted bioRxiv a license to display the preprint in perpetuity. It is made available under aCC-BY 4.0 International license.

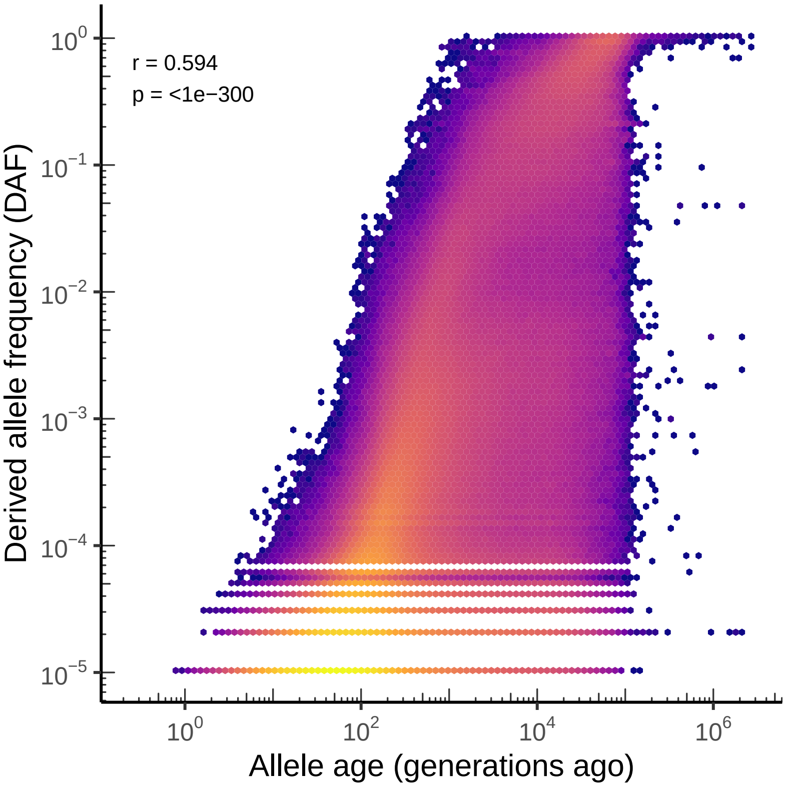

Figure S16: Log–log relationship between allele age (generations ago) and derived allele frequency (DAF), showing a positive correlation ($r = 0.594$, $p < 10^{-300}$), consistent with younger variants tending to segregate at lower frequency.

bioRxiv preprint doi: https://doi.org/10.64898/2026.01.07.698223; this version posted January 12, 2026. The copyright holder for this preprint (which was not certified by peer review) is the author/funder, who has granted bioRxiv a license to display the preprint in perpetuity. It is made available under aCC-BY 4.0 International license.

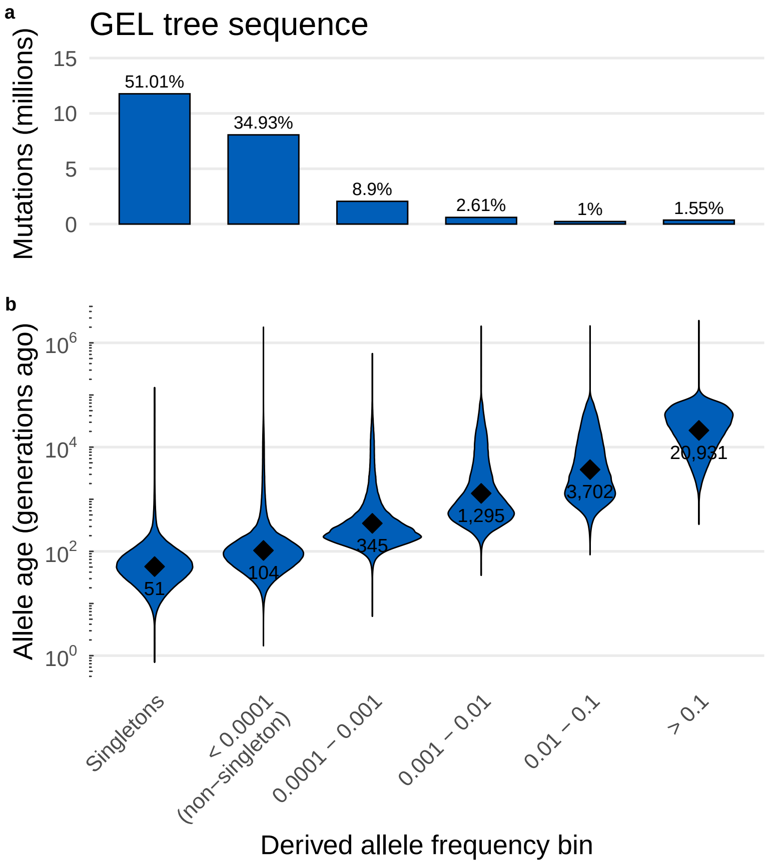

Figure S17: Allele age versus derived allele frequency in the GEL dataset. (a) Number of mutations per frequency bin (millions). (b) Violin distributions of allele age (generations ago) across the same bins, showing younger ages at lower frequencies.

bioRxiv preprint doi: https://doi.org/10.64898/2026.01.07.698223; this version posted January 12, 2026. The copyright holder for this preprint (which was not certified by peer review) is the author/funder, who has granted bioRxiv a license to display the preprint in perpetuity. It is made available under aCC-BY 4.0 International license.

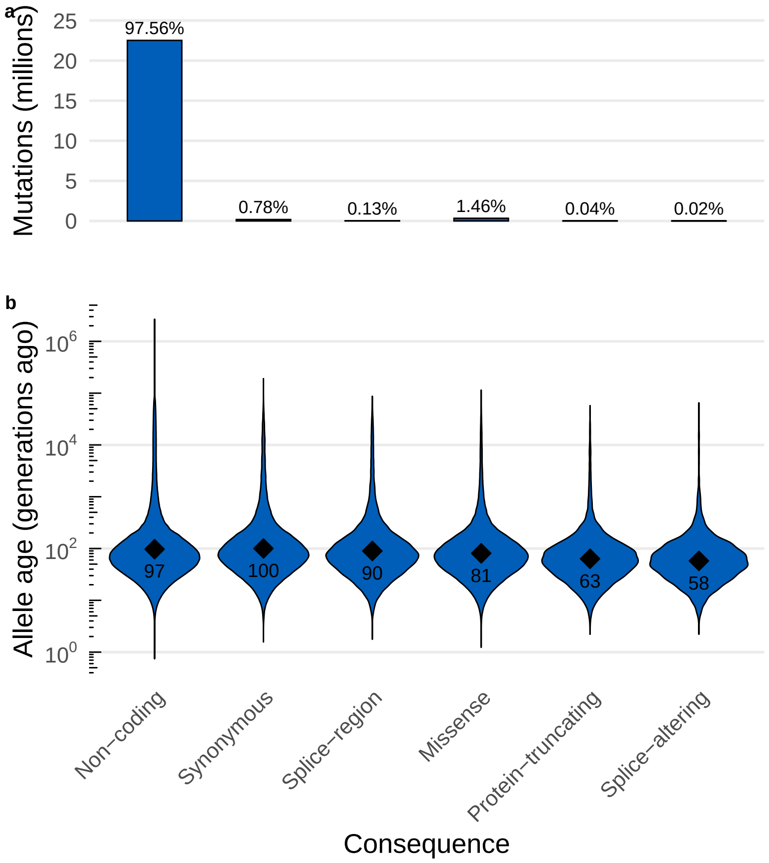

Figure S18: Allele age versus mutation consequence in the GEL dataset. (a) Number of mutations per consequence category (millions), ordered by severity. (b) Violin distributions of allele age (generations ago) across the same bins, showing younger ages more severe consequence types; points and labels show geometric mean ages. Categories shown (left to right): Non-coding, Synonymous, Splice-region, Missense, Protein-truncating, and Splice-altering as estimated using Ensembl VEP.

bioRxiv preprint doi: https://doi.org/10.64898/2026.01.07.698223; this version posted January 12, 2026. The copyright holder for this preprint (which was not certified by peer review) is the author/funder, who has granted bioRxiv a license to display the preprint in perpetuity. It is made available under aCC-BY 4.0 International license.

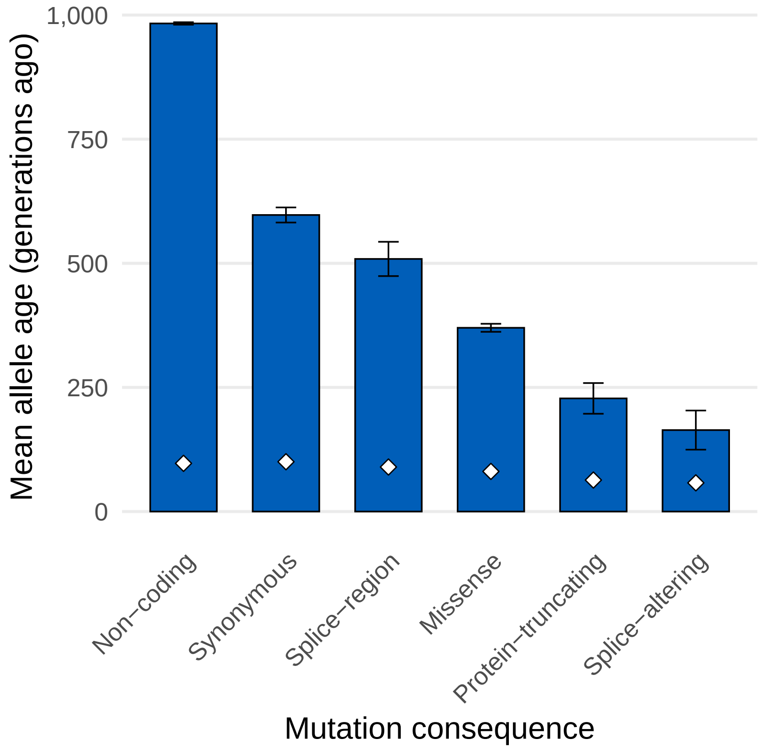

Figure S19: Mean allele age by mutation consequence in the GEL dataset. Bars show the arithmetic mean per category; error bars are 95% confidence intervals from a t-distribution (`mean_cl_t`). White diamond markers denote the geometric mean. Categories shown (left to right): Non-coding, Synonymous, Splice-region, Missense, Protein-truncating, and Splice-altering as estimated using Ensembl VEP. As expected from theory, the majority of all mutations across all categories are young (recent geometric mean ages), but a much smaller proportion of putatively deletrious alleles are old (strong decrease in arithmetic mean ages).

bioRxiv preprint doi: https://doi.org/10.64898/2026.01.07.698223; this version posted January 12, 2026. The copyright holder for this preprint (which was not certified by peer review) is the author/funder, who has granted bioRxiv a license to display the preprint in perpetuity. It is made available under aCC-BY 4.0 International license.

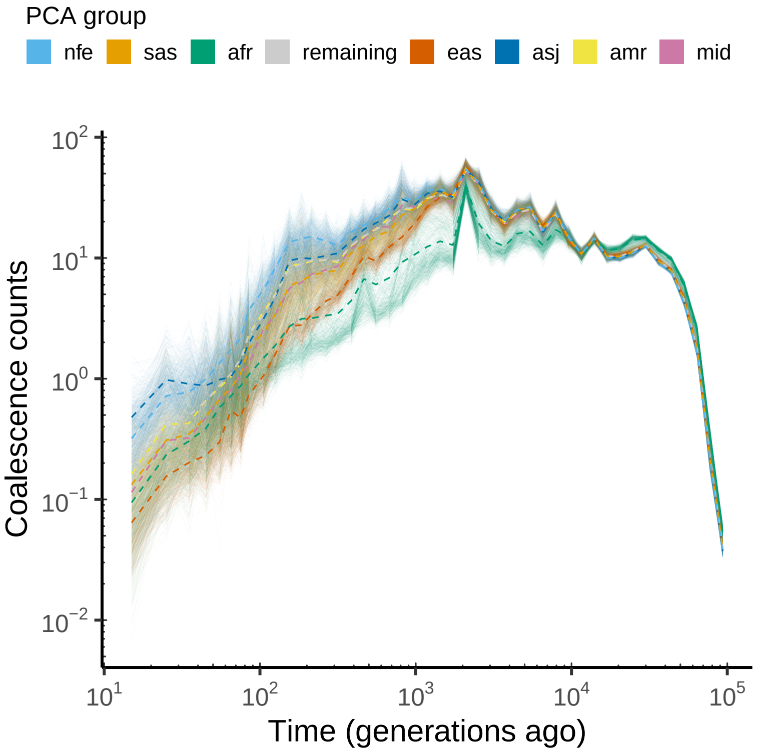

Figure S20: Pairwise coalescence counts between focal individuals and all others in the inferred ARG, coloured by PCA group. Each faint line represents a single focal individual (up to 500 per group, or all when $n < 500$), showing the number of coalescence events through time across the largest contiguous inferred ARG segment on chromosome 17q. Dashed lines indicate the mean for each PCA group. Both axes are shown on a log scale.

bioRxiv preprint doi: https://doi.org/10.64898/2026.01.07.698223; this version posted January 12, 2026. The copyright holder for this preprint (which was not certified by peer review) is the author/funder, who has granted bioRxiv a license to display the preprint in perpetuity. It is made available under aCC-BY 4.0 International license.

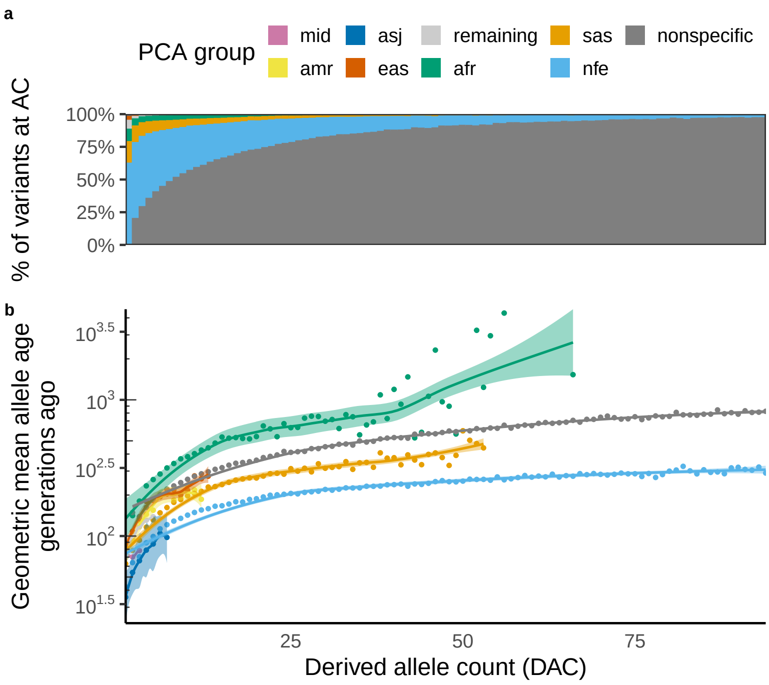

Figure S21: PCA group-stratified allele age versus derived allele count (DAC). **(A)** Proportion of variants per DAC bin across the cohort. **(B)** Geometric mean allele age (generations ago) by DAC, stratified by PCA groups (AFR, AMR, EAS, SAS, NFE, ASJ, MID) plus remaining individuals (unassigned to any one group) and a *nonspecific* category aggregating variants observed across multiple groups. Geometric means are restricted to groups with > 10 alleles in a given DAC bin.

bioRxiv preprint doi: https://doi.org/10.64898/2026.01.07.698223; this version posted January 12, 2026. The copyright holder for this preprint (which was not certified by peer review) is the author/funder, who has granted bioRxiv a license to display the preprint in perpetuity. It is made available under aCC-BY 4.0 International license.

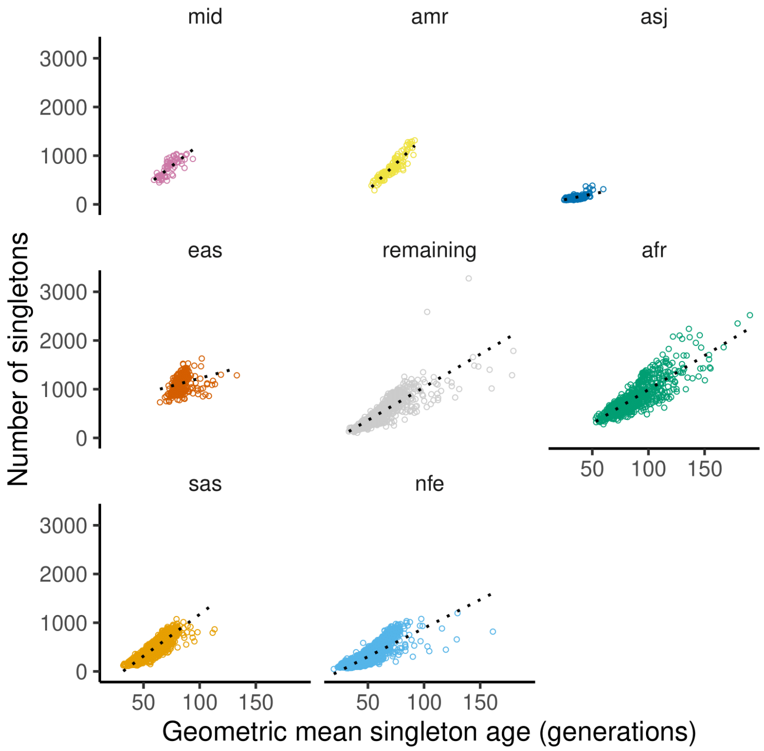

Figure S22: Number of singletons (GEL dataset-wide DAC = 1) per individuals vs the geometric mean age of those singletons, stratified by PCA groups (AFR, AMR, EAS, SAS, NFE, ASJ, MID) plus remaining individuals (unassigned to any one group). Analyses were restricted to a subset of 42,686 individuals without a < 3 degree relative in the GEL dataset.

bioRxiv preprint doi: https://doi.org/10.64898/2026.01.07.698223; this version posted January 12, 2026. The copyright holder for this preprint (which was not certified by peer review) is the author/funder, who has granted bioRxiv a license to display the preprint in perpetuity. It is made available under aCC-BY 4.0 International license.

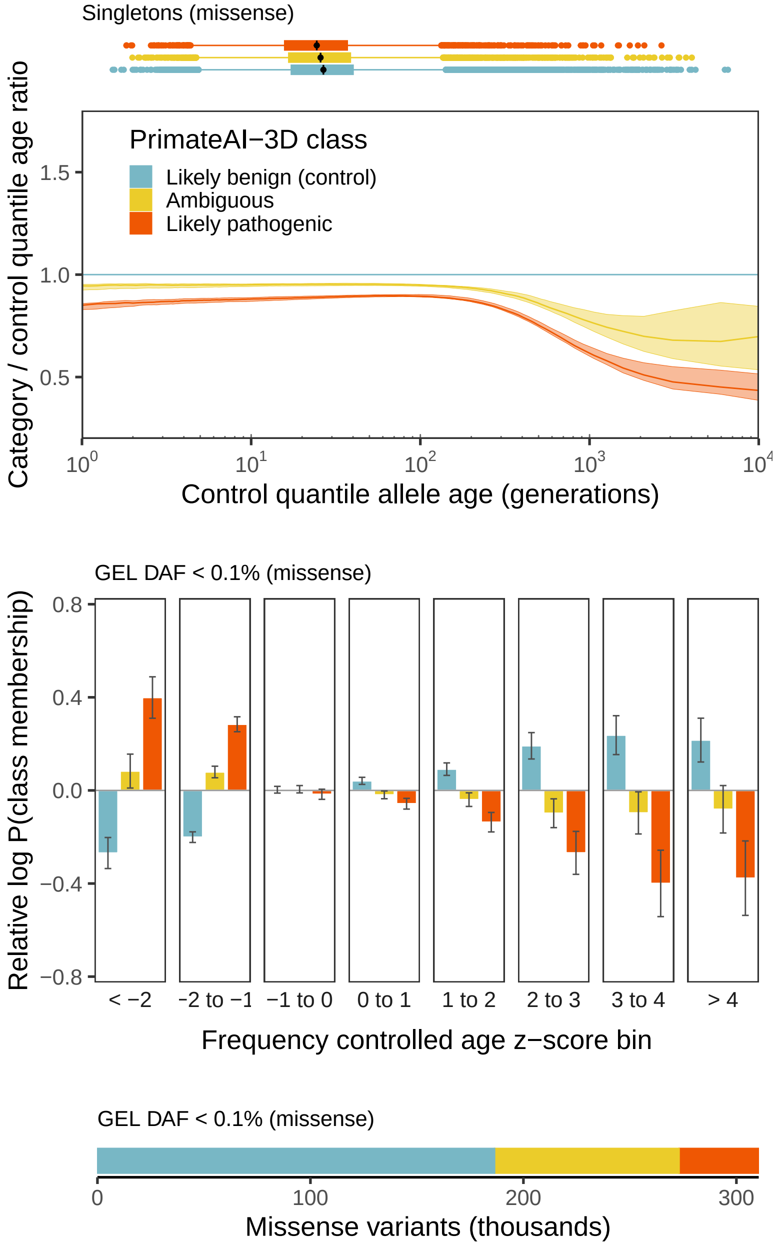

Figure S23: Allele age patterns associated with PrimateAI-3D classifications for ultra-rare missense variants. (i) Quantile–quantile analysis of singleton missense variants (DAC=1) comparing *ambiguous* and *likely pathogenic* classes against *likely benign* (control), with bootstrapped 95% confidence bands; younger ages (left) indicate stronger purifying selection. (ii) For ultra-rare missense variants (GEL DAF $< 0.1\%$), the relative probability (odds ratios) of a variant being classified as *likely pathogenic* or *ambiguous* across frequency-controlled allele-age z-score bins (youngest < -2 to oldest > 4), estimated via logistic regression with 95% confidence intervals. (iii) Counts of ultra-rare missense variants by PrimateAI-3D class.

bioRxiv preprint doi: https://doi.org/10.64898/2026.01.07.698223; this version posted January 12, 2026. The copyright holder for this preprint (which was not certified by peer review) is the author/funder, who has granted bioRxiv a license to display the preprint in perpetuity. It is made available under aCC-BY 4.0 International license.

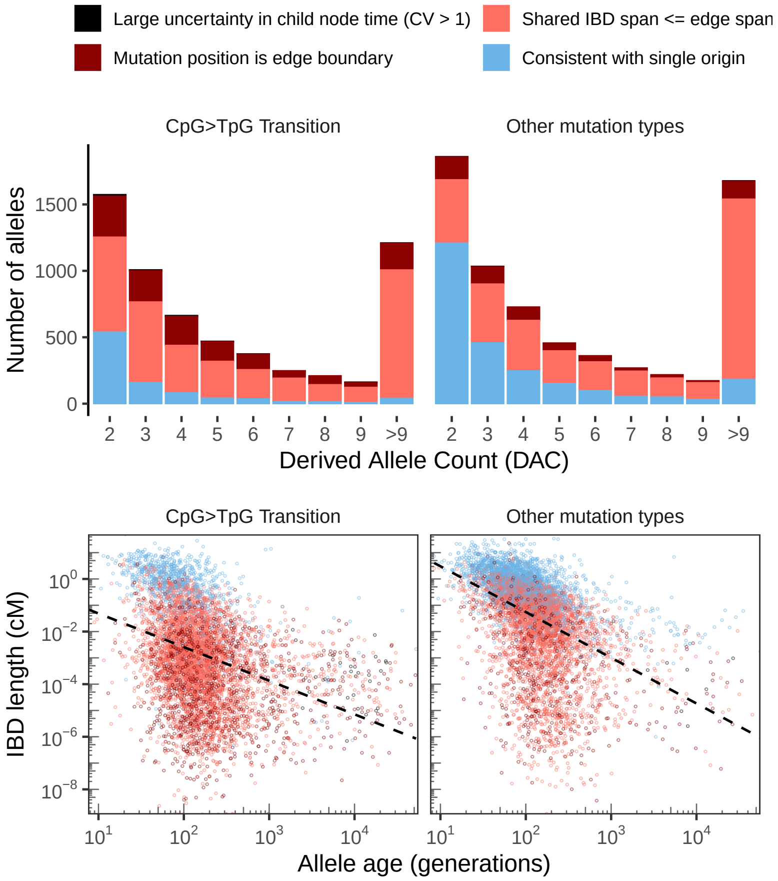

Figure S24: ARG-informed heuristics identify likely recurrent mutations and reveal expected relationships with allele age and IBD span. **Top:** Stacked bar plots showing the number of clinically classified variants (DAC > 1, DAF $< 0.1\%$) stratified by derived-allele-count (DAC) bin and mutation type (left: CpG$\rightarrow$TpG transitions; right: other mutation types). Colours indicate the outcome of the recurrence heuristic: large uncertainty in child-node time (CV > 1), mutation at edge boundary, shared IBD span $\leq$ edge span, or consistent with a single origin. CpG$\rightarrow$TpG transitions show a higher fraction flagged as likely recurrent and the proportion of flagged variants increases with DAC, consistent with an elevated probability of independent mutation events at higher allele counts. **Bottom:** Relationship between derived allele age and IBD length (cM) on a log–log scale for CpG$\rightarrow$TpG transitions (left) and other mutation types (right).

bioRxiv preprint doi: https://doi.org/10.64898/2026.01.07.698223; this version posted January 12, 2026. The copyright holder for this preprint (which was not certified by peer review) is the author/funder, who has granted bioRxiv a license to display the preprint in perpetuity. It is made available under aCC-BY 4.0 International license.

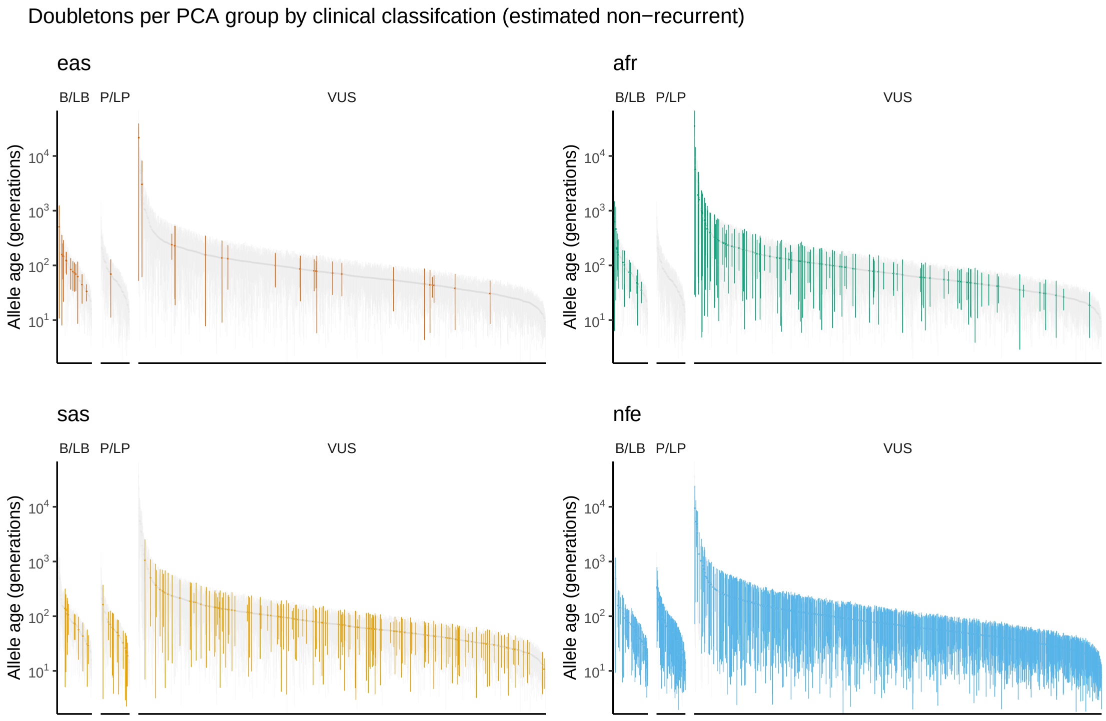

Figure S25: Allele ages for doubleton variants coloured by PCA group (AFR, EAS, SAS, NFE) and clinical classification in the GEL dataset. Points mark the estimated mutation age (generations ago); vertical intervals show the bracket between the ages of the ancestral and descendant nodes immediately above and below the mutation in the ARG. For comparison, the same variants are shown in all panels, with only those found in the corresponding PCA group colored. For each PCA group, panels correspond to the classes benign or likely benign (B/LB), pathogenic or likely pathogenic (P/LP), and variants of uncertain significance (VUS), as defined by the internal 100,000 Genomes Project rare disease clinical scientist questionnaires or ClinVar (22). Only doubletons inferred as non-recurrent (23) are shown.

bioRxiv preprint doi: https://doi.org/10.64898/2026.01.07.698223; this version posted January 12, 2026. The copyright holder for this preprint (which was not certified by peer review) is the author/funder, who has granted bioRxiv a license to display the preprint in perpetuity. It is made available under aCC-BY 4.0 International license.

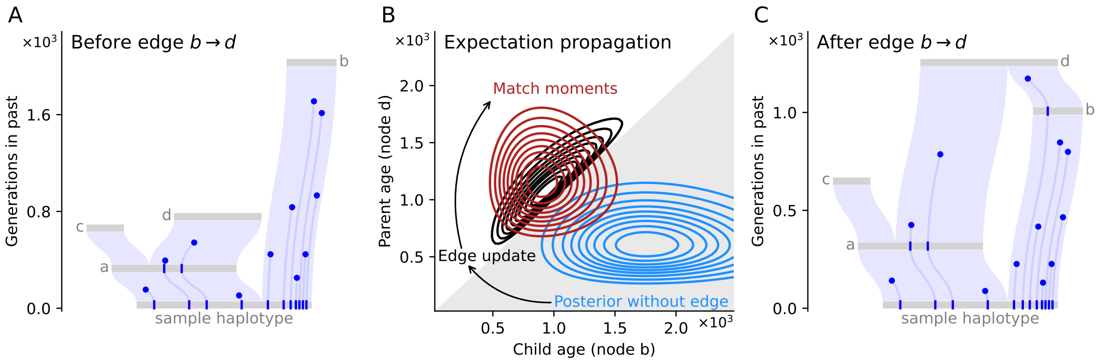

Figure S26: An example of an expectation propagation (EP) update, shown for edge $b \to d$ in Fig. 1. From left to right: (A) The dated ARG after iterating over all edges *except* $b \to d$, before the update that incorporates information from this edge into the variational posteriors of nodes b and d. (B) EP maintains an approximating factor per edge that contributes to the variational posteriors of the attached parent and child nodes. Each factor is updated by first removing it from the variational approximation; multiplying the downdated approximation by the exact (Poisson) mutational likelihood for the edge (the "surrogate"); and then reparametrizing the held-out factor such that the updated approximation has the same moments as the surrogate. Incorporating edge $b \to d$ dramatically alters the posteriors of parent (d) and child (b) as the former must be older than the latter. (C) Iterating this moment matching condition propagates information across nodes, and results in variational posteriors that reflect both mutational density on edges and the topological constraints intrinsic to the ARG.

bioRxiv preprint doi: https://doi.org/10.64898/2026.01.07.698223; this version posted January 12, 2026. The copyright holder for this preprint (which was not certified by peer review) is the author/funder, who has granted bioRxiv a license to display the preprint in perpetuity. It is made available under aCC-BY 4.0 International license.

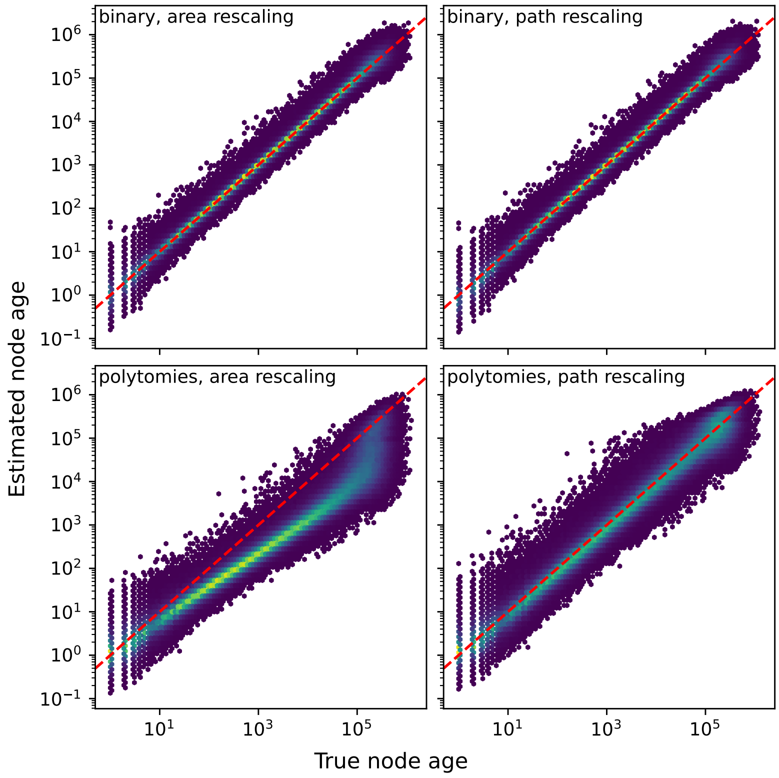

Figure S27: ARGs with artefactual polytomies are biased when rescaled by mutational area. Top row: When an ARG containing only binary trees are rescaled by either mutational area (left) or root-to-leaf path length (right), both approaches result in similarily well-calibrated time scales. Bottom row: The same ARG but with edges unsupported by mutations collapsed into polytomies, which introduces spurious mutational area. In this case, rescaling by mutational area will introduce bias and noise (left), but rescaling by root-to-leaf path length (right) will mitigate the bias. The ARG was generated via a discrete-time Wright-Fisher simulation of a randomly mating population of size 40,000 and 20,000 diploid samples; with 100Mb of sequence given a recombination rate of 10^{-8} and a mutation rate of 1.29×10^{-8}.

bioRxiv preprint doi: https://doi.org/10.64898/2026.01.07.698223; this version posted January 12, 2026. The copyright holder for this preprint (which was not certified by peer review) is the author/funder, who has granted bioRxiv a license to display the preprint in perpetuity. It is made available under aCC-BY 4.0 International license.

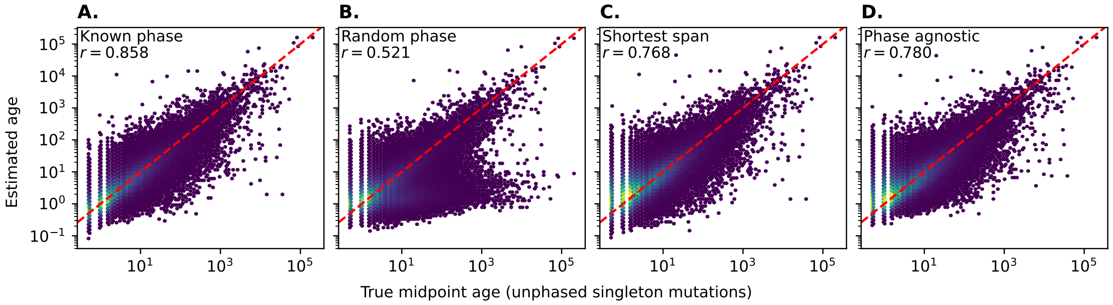

Figure S28: Phase-agnostic dating of singleton mutations. From left to right: (A) singletons dated with known phase result in unbiased age estimates (of the midpoint of the branch to which they are mapped). (B) If the phase is randomly flipped, some proportion of singletons will be mapped to longer or shorter branches and have erroneous ages. As a result, many older singletons will appear to be young. (C) Choosing singleton phase based on shorter edge span mitigates this error to a large extent. (D) The phase-agnostic dating algorithm described in SI §5 adapts this heuristic into a principled model, and propagates the uncertainty from the choice of phase into the posteriors for nodes and mutations. The ARG was generated via a discrete-time Wright-Fisher simulation of a randomly mating population of size 40,000 and 20,000 diploid samples; with 100Mb of sequence given a recombination rate of 10^{-8} and a mutation rate of 1.29×10^{-8}, and subsequently inferred via tsinfer.
